# Hydrogels: current biomedical applications and future directions

**DOI:** 10.1186/s43556-026-00525-1

**Published:** 2026-08-03

**Authors:** Haoming Wu, Guanghao Piao, Qiuqi Chen, Wuzheng Luo, Jiayu Liu, Yixuan Lan, Yuling Wang, Jixin Zhou, Kaiwen Yang, Jingjin Xiao, Qingyu Lv, Shuhao Yang, Wanyue Feng, Gaohui Zhu, Shuai Tan, Xulin Hu

**Affiliations:** 1 School of Preclinical Medicine of Chengdu University, Chengdu University, Chengdu, Sichuan 610106 China; 2 Department of Orthopaedic Surgery, Chongqing Municipal Health Commission Key Laboratory of Musculoskeletal Regeneration and Translational Medicine/Orthopaedic, Chongqing, 400010 China; 3 Department of Endocrinology Children’s Hospital of Chongqing Medical University, National Clinical Research Center for Child Health and Disorders, Ministry of Education Key Laboratory of Child Development and Disorders, China International Science and Technology Cooperation base of Child development and Critical Disorders, Chongqing Key Laboratory of Child Rare Diseases in Infection and Immunity, Chongqing, 400014 China; 4 Department of Traditional Chinese Medicine Children’s Hospital of Chongqing Medical University, National Clinical Research Center for Child Health and Disorders, Ministry of Education Key Laboratory of Child Development and Disorders, China International Science and Technology Cooperation base of Child development and Critical Disorders, Chongqing Key Laboratory of Child Rare Diseases in Infection and Immunity, Chongqing, 400014 China; 5 Clinical Medical College and Affiliated Hospital of Chengdu University, Chengdu University, Chengdu, Sichuan 610081 China; 6 Department of Biotherapy, Cancer Center and State Key Laboratory of Biotherapy, West China Hospital, Sichuan University, No.17, Section 3, Renmin South Road, Chengdu, Sichuan 610041 China

**Keywords:** Hydrogels, Biomaterials, Biomedical applications, Drug delivery systems, Tissue engineering, Regenerative medicine

## Abstract

Hydrogels are highly hydrated three-dimensional polymeric network materials that have attracted considerable attention in medical and biomedical fields owing to their favorable chemical modifiability, physical tunability, biocompatibility, and capacity to mimic key features of native extracellular matrices. With advances in polymer chemistry, crosslinking strategies, stimuli-responsive design, and emerging fabrication technologies, hydrogels have evolved from simple soft materials into multifunctional biomedical systems capable of integrating controlled delivery, tissue support, microenvironmental regulation, biosensing, and disease modeling. These properties make hydrogels particularly relevant for addressing a broad spectrum of human diseases in which conventional therapeutic strategies are often limited by insufficient targeting efficiency, limited therapeutic windows, pronounced systemic side effects, and inadequate restoration of damaged tissue structure and function. In this review, we systematically summarize the design principles and material engineering strategies of hydrogels, including structural construction approaches and functional regulation concepts. We then provide a comprehensive overview of their current biomedical applications, encompassing drug delivery systems, tissue engineering and regenerative medicine, biosensing and diagnostic platforms, as well as cell culture and organoid systems. Building on this foundation, we place particular emphasis on recent progress in hydrogel-based therapeutic strategies across diverse human disease contexts, including wound healing, musculoskeletal system repair, cancer, neurological diseases, cardiovascular diseases, autoimmune disorders, and reproductive system diseases. Finally, we discuss the developmental potential of hydrogels in biomedicine and present an integrated perspective on their clinical translation prospects, with the aim of offering references and insights for the further application of hydrogel materials in future human disease treatment.

## Introduction

**Fig. 1:**
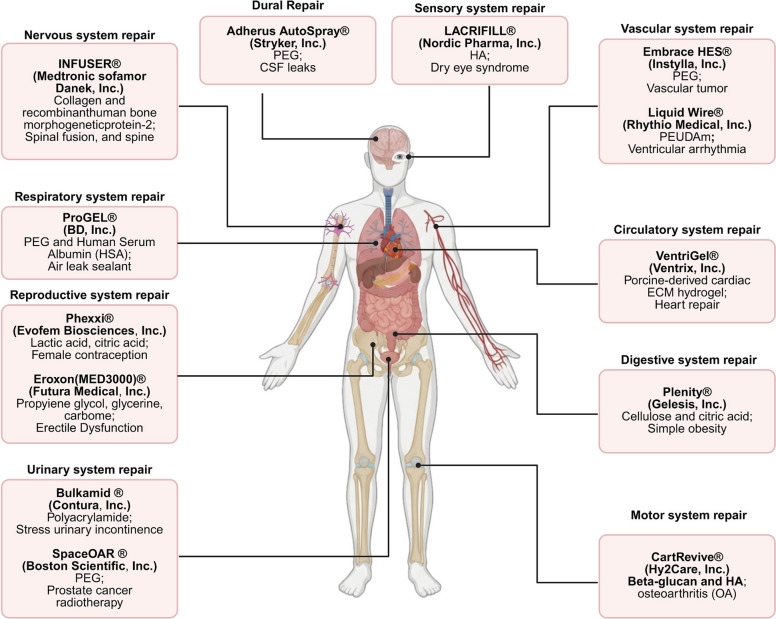
Schematic overview of representative hydrogel products approved by the U.S. Food and Drug Administration for the treatment of diseases affecting different human organ systems (data source: https://www.accessdata.fda.gov/scripts/cder/ob/index.cfm). Created using BioRender

Hydrogels are highly hydrophilic, three-dimensional crosslinked polymer networks capable of absorbing and retaining large amounts of water while maintaining structural integrity. Owing to their tissue-like softness, permeability, and ability to mimic certain features of the native extracellular matrix, hydrogels have become important biomaterial systems in tissue repair, cell survival support, and local microenvironmental regulation [1, 2]. With continuous advances in polymer chemistry, supramolecular self-assembly, and fabrication techniques, modern hydrogels have achieved a high degree of tunability and customizability, enabling their physicochemical and biological properties to be tailored for diverse biomedical applications [3, 4]. Hydrogels can function not only as drug delivery systems, allowing spatially and temporally controlled release at specific sites, but also as tissue engineering scaffolds that provide structural support and facilitate the repair of tissues such as cartilage, myocardium, and neural tissue [5, 6]. In addition, hydrogels can be integrated into biosensing and diagnostic systems to enable real-time monitoring of changes in local pathological microenvironments, thereby further enhancing therapeutic precision [7, 8]. Moreover, the rapid development of technologies such as bioprinting, microfluidics, and organoid engineering has continued to expand the role of hydrogels in disease modeling and disease treatment [9, 10]. To date, several hundred hydrogel-based medical products have received approval from the U.S. Food and Drug Administration (FDA) and have been applied across a broad spectrum of clinical settings, ranging from drug delivery and tissue repair to disease diagnosis (Fig. 1) [11, 12].

**Fig. 2:**
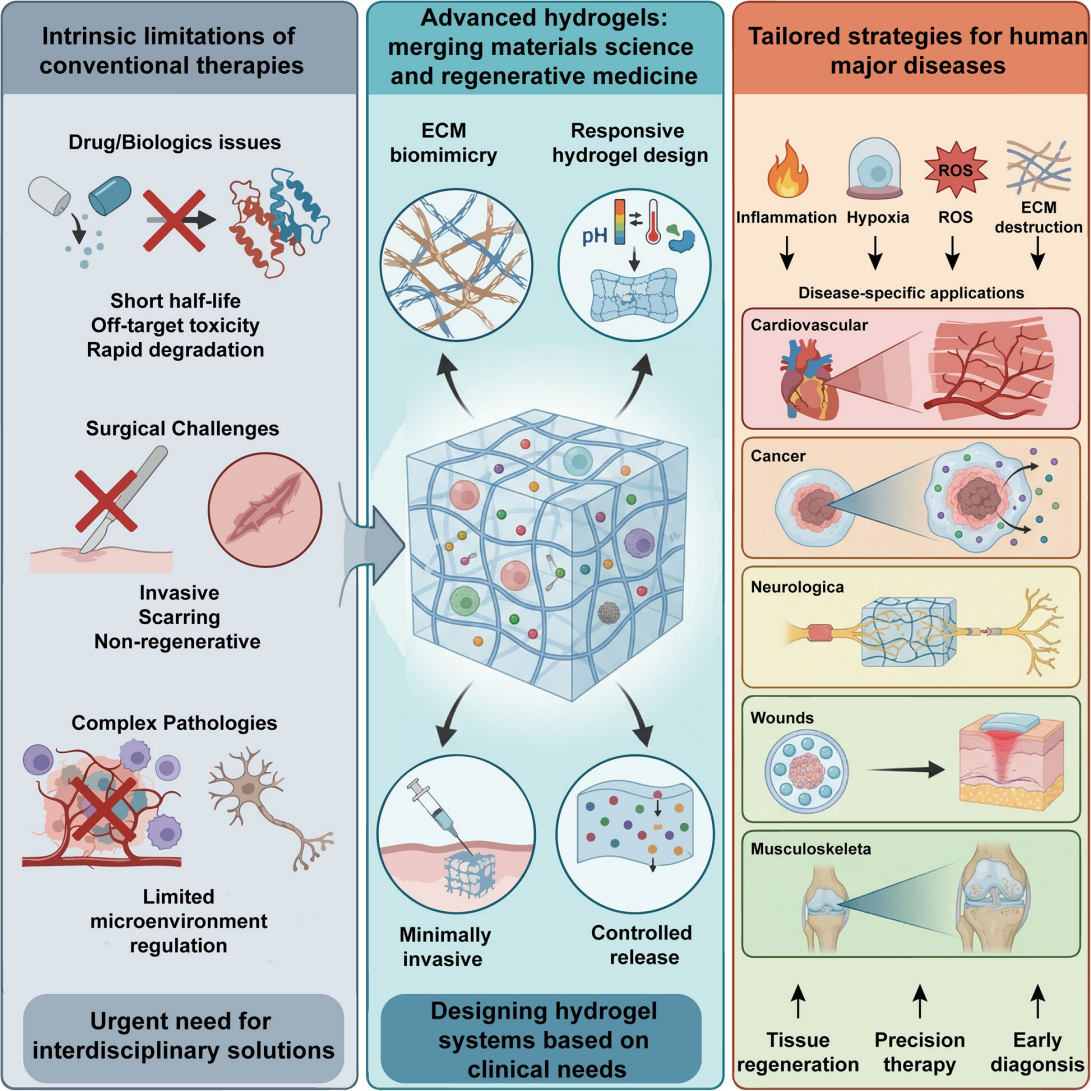
Schematic overview of the rationale and biomedical design logic of advanced hydrogel systems. The figure summarizes the intrinsic limitations of conventional therapeutic strategies, including poor stability and rapid degradation of drugs or biologics, off-target toxicity, surgical invasiveness, scarring, non-regenerative outcomes, and limited regulation of complex pathological microenvironments. Advanced hydrogels integrate key material features such as extracellular matrix biomimicry, stimuli-responsive design, minimally invasive administration, and controlled release, enabling hydrogel systems to be designed according to specific clinical needs. Representative disease-oriented applications of hydrogels, including cardiovascular diseases, cancer, neurological disorders, wound repair, and musculoskeletal diseases, are also illustrated, highlighting their potential roles in tissue regeneration, precision therapy, and early diagnosis. Created using Adobe Illustrator

The growing biomedical interest in hydrogels is closely related to the persistent limitations of conventional therapeutic strategies for diverse human diseases. Throughout the course of human history, acute and chronic diseases have continuously threatened human survival and quality of life. Traumatic injuries, cancer, metabolic disorders, and autoimmune diseases have all exerted profound impacts on global public health and socioeconomic development [13, 14]. Notably, ongoing advances in modern medicine have led to progressively deeper understanding of disease mechanisms and therapeutic strategies, with corresponding improvements in human life expectancy and health outcomes [15, 16]. For example, surgical techniques have become safer and more precise, pharmacological therapies increasingly emphasize targeted intervention, and certain modern diagnostic approaches are able to identify pathological changes associated with disease at earlier stages and with greater accuracy. Despite these advances, modern medicine continues to face substantial challenges in the treatment of many clinically relevant diseases. Many conditions remain difficult to cure, are associated with high recurrence rates, and exhibit therapeutic outcomes that are often constrained by the limitations of existing treatment paradigms. At the same time, with the acceleration of global population aging and the rising prevalence of lifestyle-related chronic diseases, there is growing demand for therapeutic approaches that offer higher levels of efficacy and precision (Fig. 2) [17, 18].

More specifically, when addressing diseases that involve complex tissue repair and functional reconstruction, conventional therapeutic strategies have exhibited clear limitations. Pharmacological treatments are often constrained by short half-life, off-target distribution, systemic toxicity, and insufficient accumulation within diseased tissues, making it difficult to maintain effective concentrations at sites where they are most needed [19, 20]. Although surgical interventions can effectively remove lesions or repair damage, they are typically highly invasive and may introduce secondary injury, infection risk, scar formation, or incomplete functional recovery [21, 22]. In the treatment of refractory cancers, radiotherapy and chemotherapy can achieve a certain degree of therapeutic benefit; however, they are frequently accompanied by severe side effects, the development of drug resistance, and limited capacity to modulate the tumor microenvironment [23, 24]. Similarly, for chronic inflammatory, neurodegenerative, and metabolic diseases, existing treatments often focus on symptom alleviation rather than regenerative repair, and therefore struggle to restore tissue architecture or correct underlying biological imbalances [25, 26]. In addition, despite rapid progress in advanced therapies such as growth factors, nucleic acids, and stem cells, their inherent biological instability often necessitates protective and controllable delivery systems to preserve bioactivity (Fig. 2) [27, 28]. At present, as our understanding at the cellular and molecular levels continues to deepen, therapeutic strategies are gradually shifting from single-modality approaches toward integrated treatment paradigms in order to address increasingly complex pathological microenvironments.

In this context, hydrogels provide a materials-based strategy that links biomedical engineering with disease-oriented therapeutic needs. Their highly hydrated network structure enables local retention and controlled release of therapeutic agents, while their tunable mechanical properties and degradation behavior allow them to better match the requirements of different tissues and stages of repair. In addition, stimuli-responsive and functionalized hydrogels can sense pathological cues such as pH changes, oxidative stress, enzymatic activity, or inflammatory signals, and translate these cues into controlled drug release, structural remodeling, or microenvironmental regulation. These features make hydrogels particularly suitable for disease settings characterized by chronic inflammation, fibrosis, oxidative stress, immune dysregulation, impaired vascularization, or limited regenerative capacity. Therefore, hydrogels are increasingly being explored not only as passive carriers or fillers, but also as interactive biomedical systems capable of participating in tissue repair, disease modulation, and precision treatment.

This review aims to provide a comprehensive overview of recent advances and clinical applications of hydrogels across diverse human disease contexts (Fig. 2). We first summarize the design principles and material engineering of hydrogels, including polymer chemistry, crosslinking strategies, tunable mechanical and degradation properties, stimuli-responsive design, biofunctionalization strategies, and emerging hydrogel-based fabrication approaches such as three-dimensional printing and microfluidic technologies. We then review the current biomedical applications of hydrogels, covering drug delivery systems, tissue engineering scaffolds, biosensing and diagnostic platforms, as well as cell culture and organoid systems. Subsequently, we focus on recent progress in hydrogel-based strategies for a wide range of clinically relevant diseases, including wound healing, musculoskeletal disorders, cancer, neurological diseases, cardiovascular diseases, autoimmune disorders, and reproductive system diseases. Finally, we discuss future perspectives on hydrogel strategies in biomedicine and provide a comprehensive outlook on their clinical translation, with the goal of offering a concise snapshot of how hydrogels may contribute to advances in human healthcare.

## Design principles and material engineering of hydrogels

**Fig. 3:**
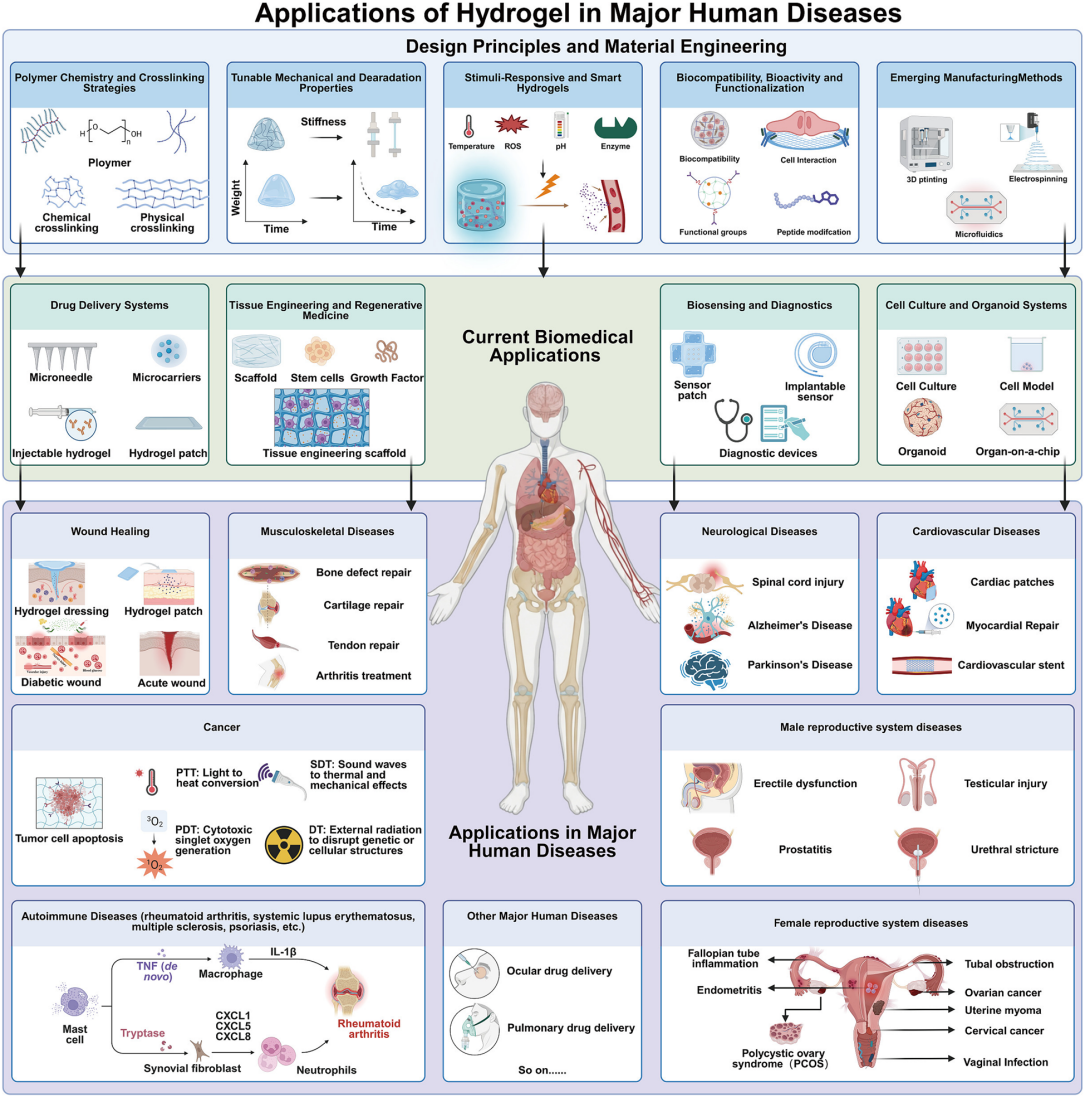
Schematic overview of hydrogel design principles, biomedical applications, and disease-oriented therapeutic strategies. The figure summarizes key aspects of hydrogel material engineering, including crosslinking strategies, tunable mechanical and degradation properties, stimuli-responsive functionalities, biocompatibility, and emerging fabrication methods, as well as their current applications in drug delivery, tissue engineering, biosensing, and cell culture. Representative applications of hydrogels in diverse disease contexts, such as wound healing, musculoskeletal disorders, cancer, neurological, cardiovascular, autoimmune, and reproductive system diseases, are also illustrated. Created using BioRender

The biomedical performance of hydrogels is determined not only by their hydrated network structure, but more importantly by the coordinated regulation of polymer composition, crosslinking chemistry, mechanical behavior, degradation kinetics, stimulus responsiveness, bioactivity, and fabrication methods. These parameters collectively define how hydrogels interact with cells, tissues, therapeutic agents, and pathological microenvironments. Therefore, this section focuses on the material-level design logic underlying hydrogel function, rather than reintroducing their general biomedical applications. We first discuss polymer chemistry and crosslinking strategies as the structural foundation of hydrogel systems, followed by mechanical and degradation tuning, stimuli-responsive behavior, biofunctionalization, and emerging manufacturing methods. This framework provides the material basis for the biomedical application platforms and disease-oriented strategies discussed in subsequent sections (Fig. 3).

### Polymer chemistry and crosslinking strategies

#### Polymers constituting hydrogels

The structure and function of hydrogels essentially originate from their underlying polymer chemistry, while polymer composition and crosslinking modes jointly determine the microscopic network architecture, macroscopic mechanical behavior, degradation profile, and interaction patterns with biological systems. From a materials construction perspective, polymers used for hydrogel fabrication can generally be classified into natural polymers and synthetic polymers, which exhibit complementary advantages in terms of bioactivity and design controllability [29, 30].

Natural polymer-based hydrogels, including chitosan, gelatin, alginate, and hyaluronic acid, retain abundant chemical motifs that resemble components of the native extracellular matrix (ECM). These polymers can participate in the regulation of cellular behaviors through receptor-ligand interactions, adhesion-related signaling, and matrix-associated biochemical cues, and have therefore been widely applied in regenerative medicine and tissue engineering research [5, 31]. For example, hyaluronic acid-based hydrogel systems can regulate cell migration, proliferation, and differentiation through interactions with receptors such as CD44, highlighting the advantages of natural polymers in presenting endogenous bioactive signals [32]. However, natural polymers often exhibit complex molecular compositions, batch-to-batch variability, and a relatively limited range of tunable mechanical properties, which may restrict their use in highly engineered and reproducible manufacturing scenarios [33].

Synthetic polymer-based hydrogels, such as those based on polyethylene glycol (PEG), polyvinyl alcohol (PVA), polyacrylic acid (PAA), and polylactic acid (PLA), provide greater control over molecular architecture, chemical composition, mechanical strength, and degradation kinetics [34]. PEG-based hydrogels, for instance, are widely used as model systems for constructing defined cellular microenvironments because their network parameters can be systematically regulated to study cell–matrix interactions across spatial and temporal scales [35]. Nevertheless, synthetic polymers generally lack intrinsic biological recognition motifs, and therefore often require additional functionalization or hybridization with bioactive components to support appropriate cell adhesion, signaling, or tissue integration [36].

In this context, natural-synthetic composite hydrogel systems have become a major research direction. Through copolymerization, block copolymer design, interpenetrating polymer networks, or physical–chemical hybridization, composite hydrogels can integrate the bioactivity of natural polymers with the structural reproducibility and mechanical tunability of synthetic systems [37]. Overall, hydrogel polymer systems are evolving from single-source materials toward multi-component platforms that balance biological activity, physicochemical stability, and engineering controllability.

#### Chemical modification of hydrogels

After the polymer backbone is selected, chemical modification provides a key route for introducing bioactivity, stimulus responsiveness, and interfacial functionality into hydrogel networks. One established strategy involves incorporating cell-adhesive ligands, such as RGD and related peptide sequences, into hydrogel matrices to mimic ECM-integrin interactions and reconstruct signaling at the material-cell interface [38]. The density, spatial distribution, and presentation mode of these ligands can strongly influence cell adhesion, migration, proliferation, and differentiation, making ligand engineering an important foundation for hydrogel biointerface design [39].

Further chemical modification strategies introduce reactive groups, dynamic bonds, degradable motifs, or nanofunctional units into hydrogel networks, enabling materials to integrate mechanical reinforcement, stimulus responsiveness, and therapeutic activity within a single system [40, 41]. These modular chemistry approaches allow hydrogels to move beyond passive scaffolds or simple carriers and become instructive biomaterial platforms capable of linking molecular design with biological outcomes.

#### Crosslinking strategies of hydrogels

Crosslinking is a central determinant of hydrogel network topology and dynamic behavior, and it directly influences mechanical properties, permeability, degradation, and environmental adaptability [34]. Physically crosslinked hydrogels are formed through reversible noncovalent interactions, such as hydrogen bonding, ionic interactions, hydrophobic interactions, host–guest interactions, and polymer chain entanglements. These networks usually form under mild conditions and can exhibit dynamic properties such as injectability, shear-thinning behavior, stress relaxation, and self-healing, which are particularly useful for applications requiring minimally invasive administration or adaptive tissue interaction [30]. Recent studies have also highlighted that topological entanglements between polymer chains can provide important mechanical constraints, offering new strategies for improving hydrogel toughness without relying solely on additional crosslinking agents [42].

Chemical crosslinking forms stable networks through covalent bonds, allowing hydrogels to maintain structural integrity under physiological conditions and long-term mechanical loading. This strategy is widely used in applications requiring durable implantation or sustained functional performance. However, conventional chemical crosslinking can involve harsh reaction conditions, potential side reactions, or network brittleness. The development of bioorthogonal and click chemistries, including strain-promoted azide-alkyne cycloaddition, inverse electron-demand Diels–Alder reactions, and thiol-ene reactions, has improved the precision, selectivity, and biocompatibility of covalently crosslinked hydrogel fabrication [43].

Dynamic covalent crosslinking provides a balance between structural stability and network reversibility. Reversible covalent bonds, such as imine bonds, disulfide bonds, hydrazone bonds, and boronate ester linkages, enable hydrogels to exhibit self-healing, stress relaxation, reconfigurability, and stimulus-responsive remodeling while maintaining overall network cohesion [44]. These properties are highly relevant to injectable hydrogels, adaptive scaffolds, and responsive drug delivery platforms. Overall, physical, chemical, and dynamic covalent crosslinking strategies each have distinct strengths and limitations, and future hydrogel design is likely to rely increasingly on hybrid crosslinking systems that integrate mechanical robustness, reversible remodeling, degradation control, and functional programmability [29, 40].

### Tunable mechanical properties and degradation behavior

#### Tunable mechanical properties of hydrogels

The tunable mechanical properties of hydrogels constitute one of the fundamental reasons for their broad biomedical application. Their mechanical behavior is determined by multiscale structural factors, including polymer chain composition, molecular weight, chain flexibility, crosslinking density, network topology, water content, and microstructural organization [5, 29, 30]. Natural polymers such as alginate, hyaluronic acid, and gelatin provide ECM-related bioactivity and favorable biocompatibility, but their relatively flexible chains and limited crosslinking stability can restrict mechanical durability [31–33]. In contrast, synthetic polymer systems such as PEG and polyacrylamide offer highly controllable molecular structures and predictable synthesis, allowing mechanical properties to be tuned over a broader range [5, 30, 34]. Accordingly, polymer selection and network design must be considered together when tailoring hydrogels for specific biomedical contexts.

Under a fixed polymer composition, crosslinking strategy is a key determinant of mechanical behavior. Chemically crosslinked networks provide relatively high structural stability and are suitable for long-term implantation or load-bearing environments, whereas physically crosslinked networks rely on reversible interactions and therefore provide dynamic features such as self-healing, stress relaxation, and adaptive deformation [5, 34, ,37 36 ]. To overcome the mechanical limitations of conventional single-network hydrogels, double-network and multinetwork strategies have been developed. By combining a highly crosslinked brittle network with a ductile and extensible network, these systems can markedly increase toughness and fracture energy, expanding the use of hydrogels in mechanically demanding biomedical scenarios [40, 41].

Microstructural and nanostructural engineering further broadens the mechanical design space of hydrogels. Nanoreinforcement using high-modulus components such as nanoclays, graphene oxide, or nanocellulose can create multilevel energy dissipation pathways and improve strength and toughness while maintaining a high water content [41, 42]. Biomimetic fibrillar structures fabricated by directional freezing, electrospinning, or microfluidic fiber assembly can generate anisotropic networks that resemble tissue-specific structural textures and mechanical gradients [45–47]. With the development of three-dimensional bioprinting, spatial photopolymerization, and multiscale manufacturing technologies, continuous mechanical gradients can now be constructed within a single hydrogel system to mimic complex tissue interfaces, such as cartilage-bone and tendon-bone transition regions [48, 49]. These strategies indicate that hydrogel mechanics should be understood not only as bulk stiffness, but also as a combination of strength, toughness, viscoelasticity, anisotropy, stress relaxation, and spatial organization.

#### Degradation properties of hydrogels

Degradation behavior directly determines the in vivo residence time of hydrogels and the duration of their biological or therapeutic functions. Hydrogel degradation usually results from interactions between the polymer network and the biological environment and may involve hydrolysis, enzymatic cleavage, swelling-induced disintegration, or physically triggered network dissociation [50]. Networks containing hydrolytically labile bonds, such as ester or amide linkages, often exhibit relatively predictable degradation kinetics, whereas enzyme-degradable hydrogels can respond to local tissue remodeling or disease-associated enzymatic activity. Physically crosslinked systems may undergo faster disassembly in response to changes in ionic strength, pH, or competitive binding, making them suitable for short-term delivery or injectable applications [36, 37].

Crosslinking density is a central structural parameter that simultaneously regulates permeability, diffusion, mechanical support, and degradation rate. Higher crosslinking density generally improves mechanical stability but may restrict water penetration, enzyme access, and tissue ingrowth, whereas lower crosslinking density accelerates degradation but may compromise early structural support [50]. Therefore, mechanical stability, degradation time window, and tissue remodeling rate need to be coordinated rather than optimized independently. In tissue engineering, this balance is particularly critical. If degradation occurs too rapidly, newly deposited ECM may not provide sufficient support; if degradation is too slow, the material may hinder tissue ingrowth or induce chronic inflammation [51, 52].

To better synchronize hydrogel degradation with tissue repair, stepwise degradable networks, spatially heterogeneous crosslinking architectures, enzyme-sensitive peptide linkers, and programmable degradable bonds have been developed [53, 54]. These approaches enable hydrogels to exhibit stage-specific degradation behavior and functional compartmentalization during tissue repair. From a broader perspective, degradable hydrogels are no longer regarded as passive scaffolds that simply disappear over time, but as dynamic materials that participate in tissue remodeling.

### Stimuli-responsive and smart hydrogels

With advances in materials science, molecular engineering, and biomedical research, hydrogel design has gradually shifted from inert support toward functional systems with environmental sensing, dynamic regulation, and feedback capability. Early hydrogels mainly relied on hydration, softness, and network storage capacity for tissue substitution or sustained release. More recent systems incorporate responsive or reconfigurable motifs into polymer networks, allowing hydrogels to recognize disease-associated microenvironmental cues and generate programmable structural or functional responses [55–57]. Although the terms “stimuli-responsive hydrogels” and “smart hydrogels” are sometimes used separately, they largely overlap in material composition, response mechanisms, and application objectives. A more coherent interpretation is to regard them as functional hydrogels designed for dynamic regulation of biological or pathological microenvironments, with differences mainly reflecting the degree of response complexity and system integration [57, 58].

The onset and progression of many diseases are accompanied by characteristic changes in local tissue microenvironments, including mild acidity in tumors and inflamed tissues, ROS accumulation caused by disrupted redox homeostasis, hypoxia in ischemic or degenerative tissues, abnormal enzyme expression in infection or cancer, and altered ionic or metabolic conditions [59, 60]. These disease-associated cues provide intrinsic triggers for hydrogel design. By incorporating responsive bonds, ionizable groups, degradable motifs, or functional nanocomponents, hydrogels can translate microenvironmental changes into material outputs such as swelling, network degradation, conformational change, altered permeability, mechanical remodeling, or controlled drug release [61]. This design logic provides the material basis for lesion-localized therapy, but detailed disease-specific applications are discussed in later sections to avoid conceptual repetition.

Among widely used stimuli, temperature, pH, redox state, light, and enzymatic activity are the most representative [62]. Thermo-responsive hydrogels can undergo sol–gel transitions near physiological temperature and are widely explored for injectable delivery and local therapy [63, 64]. pH-responsive hydrogels, often constructed by protonatable groups or acid-labile dynamic bonds, can respond to acidic tumor or inflammatory microenvironments [65]. Redox-responsive systems exploit abnormal glutathione or ROS levels through linkages such as disulfide and diselenide bonds [66, 67]. Photo-responsive hydrogels use external light to regulate network structure, trigger photothermal or photodynamic effects, or control spatially defined release [68, 69]. Enzyme-responsive hydrogels incorporate cleavable peptide or polymer motifs that respond to disease-associated enzymes, enabling lesion-dependent degradation or functional activation [70]. These mechanisms have become foundational design tools for drug delivery, cancer therapy, wound repair, immune modulation, and diagnostic systems. Because real disease microenvironments contain multiple overlapping signals, single-stimulus systems may show insufficient selectivity or unstable responses in vivo. Multi-stimuli-responsive hydrogels have therefore emerged as an important research direction [71]. By integrating response modules such as pH/ROS, temperature/light, or enzyme/ROS combinations, hydrogels can achieve more selective activation and reduce nonspecific release through logic-like response behavior [72]. In parallel, dynamic networks with self-healing, shear-thinning, adaptive mechanics, and structural recovery have expanded the functional scope of responsive hydrogels [37, 40]. Reversible crosslinking based on dynamic covalent bonds, metal–ligand coordination, or multiple hydrogen-bonding interactions can restore material integrity after mechanical damage, which is particularly useful for long-term implantable materials and flexible biomedical devices [73]. Mechanically adaptive hydrogels that modulate stiffness or structure in response to cellular traction forces or external stress provide additional opportunities for modeling tumor stiffening, fibrosis progression, and mechanobiological regulation [74, 75].

Higher-level functional hydrogels increasingly integrate sensing and therapeutic modules within a single system [76, 77]. By incorporating pH-, ROS-, enzyme-, temperature-, or inflammation-sensitive sensing units, hydrogels can monitor local lesion states and trigger drug release, photothermal activation, or immunomodulatory responses when specific thresholds are reached [78, 79]. These systems represent an early step toward closed-loop therapeutic materials. However, their clinical translation remains limited by heterogeneity of disease signals, possible off-target activation, incomplete understanding of long-term biosafety, and difficulties in manufacturing reproducibility and scale-up [45, 60]. Looking ahead, multiscale computational modeling, artificial intelligence-assisted material design, programmable biofabrication, and synthetic biology may help improve the predictability, selectivity, and reliability of responsive hydrogels [80].

### Biocompatibility, bioactivity, and functionalization strategies

As hydrogel design has shifted from structural support toward microenvironment regulation and biological function construction, biocompatibility, bioactivity, and functionalization have become key determinants of biomedical applicability and clinical feasibility [81, 82].

Biocompatibility and bioactivity strategies of hydrogels. Biocompatibility is not limited to the absence of obvious cytotoxicity or inflammation. In a broader sense, it refers to the ability of a material to maintain physiological homeostasis, avoid disruptive tissue responses, coordinate with degradation and remodeling processes, and support appropriate interactions with cells, proteins, immune components, and surrounding ECM [83, 84]. The high water content and soft mechanical nature of hydrogels make them more similar to native ECM than many rigid materials, thereby reducing mechanical mismatch at tissue interfaces [85, 86]. Nevertheless, long-term biocompatibility is also affected by degradation products, residual initiators or crosslinkers, nonspecific protein adsorption, immune-cell responses, and the stability of bioactive components [87–89]. Therefore, hydrogel biocompatibility should be regarded as a cross-scale biointerface engineering issue rather than a single static material property.

Bioactivity is the attribute that enables hydrogels to move from being compatible materials to instructive platforms. Natural polymer systems have inherent advantages because they contain ECM-related recognition motifs, such as glycosaminoglycan-associated sulfated groups, RGD-containing sequences in gelatin, and ionically crosslinked domains in alginate, which can support cell adhesion, migration, differentiation, and matrix remodeling [90]. However, natural materials often exhibit structural heterogeneity, limited control over degradation, and a narrow engineering window [91]. Synthetic polymer systems provide more controllable structural backgrounds for reconstructing bioactivity. For example, ECM-derived peptide motifs such as RGD, IKVAV, and YIGSR can be introduced into otherwise inert networks, and their density, spatial distribution, and presentation mode can be adjusted to regulate cell behavior [92]. In addition, bioorthogonal chemistry enables growth factors, glycosylated structures, immunomodulatory molecules, or degradable linkers to be covalently or reversibly tethered to polymer chains under mild and selective conditions, allowing spatially localized and sustained signal presentation [43, 89].

Functionalization strategies of hydrogels With the increasing understanding of disease-associated microenvironments, hydrogel functionalization has expanded from single biological signal presentation toward complex intervention logic. In tumor therapy, hydrogels may need to coordinate with acidic pH, ROS accumulation, enzyme overexpression, metabolic abnormalities, and immunosuppressive signals to achieve localized drug release or immune microenvironment regulation [93]. In regenerative medicine, hydrogels are expected to support cell adhesion and proliferation while also guiding tissue reconstruction through spatiotemporal presentation of mechanical, chemical, and biological cues, such as ECM-mimetic structures, mechanically defined differentiation signals, degradable domains, or immunomodulatory factors [94]. These requirements have driven hydrogels to evolve from static constructs into programmable functional systems.

The concept of dynamic bioactivity emphasizes that hydrogels should not only contain bioactive components, but should also adjust the mode, timing, and location of signal presentation in response to microenvironmental changes [95, 96]. Representative strategies include enzyme-responsive ECM mimics based on cleavable peptide sequences, ROS-sensitive release of anti-inflammatory or regenerative factors, and pH-responsive tumor immunomodulatory systems [97, 98]. In such designs, hydrogels can function as information-processing materials that convert pathological cues into structural or therapeutic outputs [99, 100].

In addition to chemical functionalization, physical architecture and topological design are increasingly recognized as important sources of bioactivity. Nanoscale topography can regulate focal adhesion, cytoskeletal tension, and stem cell fate; dynamic networks can modulate cellular traction and matrix remodeling through stress relaxation; and aligned fibers or mechanical gradients can guide axonal extension, angiogenesis, or interfacial tissue integration [101, 102]. These findings indicate that hydrogel bioactivity emerges from the integration of chemical cues, physical structure, mechanical properties, and biological signals. By further incorporating nanocarriers, degradable microparticles, gene delivery modules, or immunoactive components, hydrogels can function as multifunctional platforms for drug delivery, immune regulation, cell behavior control, and microenvironmental homeostasis [103, 104].

Overall, the biocompatibility, bioactivity, and functional design of hydrogels are undergoing a transition from ECM mimicry toward microenvironment reconstruction, from single-signal presentation toward multisignal coordination, and from static functionality toward dynamic and programmable regulation. With continued integration of polymer chemistry, crosslinking strategies, mechanical tunability, programmable degradation, and biointerface engineering, future hydrogel materials are expected to achieve more precise interactions with disease progression, cellular states, and immune responses [58, 80].

### Emerging manufacturing methods

As the biomedical role of hydrogels has expanded from simple three-dimensional scaffolds to platforms for microenvironment modulation, disease modeling, and therapeutic delivery, their fabrication strategies have also undergone substantial transformation [105]. Traditional bulk gelation and casting methods are useful for constructing basic hydrogel networks, but they are often insufficient for reproducing tissue microarchitecture, generating spatially organized biological cues, or producing personalized therapeutic systems. Cellular behaviors are regulated by multiple parameters, including ECM composition, mechanical stress, topological features, dynamic signaling, and cell–cell interactions [106]. Therefore, hydrogel manufacturing is increasingly shifting from material shaping toward biologically informed microenvironment construction.

#### 3D Bioprinting

Three-dimensional bioprinting endows hydrogels with tissue-scale spatial organization and enables them to function as bioinks for constructing cellularized and heterogeneous tissue-like structures. Hydrogels with shear-thinning behavior, self-healing capability, and rapid crosslinking are particularly suitable for printing because they allow spatial positioning of cells, multilayer assembly, and integration of structural, mechanical, and biochemical cues [107]. Photocuring-based techniques such as digital light processing use digitally controlled light fields to generate complex three-dimensional structures at relatively high throughput, while voxel-level regulation of exposure dose can create spatial variations in crosslinking density and mechanical properties [107, 108]. Two-photon polymerization provides cellular or subcellular resolution and is useful for fabricating fine guidance architectures or interfacial microstructures [109]. Extrusion-based bioprinting is more suitable for larger constructs with higher mechanical requirements, including myocardial patches, cartilage scaffolds, and tumor microenvironment models [110]. Increasingly, bioprinted hydrogels are also designed to exhibit dynamic behavior by incorporating reconfigurable networks, stress relaxation, and temporally controlled crosslinking, thereby supporting dynamic tissue models and evolvable therapeutic carriers [50, 96].

#### Microscale manufacturing technologies

Microfluidics-driven microscale manufacturing focuses on producing programmable functional microenvironment units rather than bulk tissue structures. Under controlled hydrodynamic conditions, microfluidics can generate monodisperse microgels, core–shell particles, Janus structures, and multicompartment modules with tunable size, morphology, internal gradients, and degradation profiles [111, 112]. These microgels can serve as injectable cell carriers, localized drug reservoirs, immunoregulatory units, or modular building blocks for assembling larger hydrogel systems [111–113]. Microfluidic technologies also facilitate organ-on-a-chip platforms, in which dynamic fluid stimulation, compartmentalized tissue organization, and controllable biochemical inputs allow reconstruction of organ-level disease processes such as inflammation, barrier dysfunction, tumor invasion, and metastasis [114–116]. In these systems, hydrogels function not as isolated materials, but as integral components of engineered biological models.

#### Micro- and nanofabrication technologies

Micro- and nanofabrication techniques provide hydrogels with higher structural precision across length scales ranging from single-cell microenvironments to tissue-scale architectures. Electrospun fibrous networks can mimic ECM fibrillar structures and guide neuronal axon extension, endothelial alignment, or muscle contraction [117, 118]. Nanolithography and template-assisted fabrication can generate ordered nanotopographical features such as nanogrooves and nanopillars, which regulate focal adhesion maturation, cytoskeletal organization, nuclear deformation, and downstream transcriptional pathways [119]. The combination of micro/nanotopography with dynamic crosslinking and mechanically tunable hydrogels further enables adaptive microenvironments in which structural features and cellular forces interact over time [120, 121]. Such fabrication strategies are particularly useful for studying cell–matrix interactions and for designing hydrogels with tissue-specific guidance functions.

#### Programmable manufacturing

Programmable manufacturing advances hydrogel fabrication from spatial construction toward spatiotemporal control. Through grayscale light fields, programmable photopolymerization, digital patterning, and multicomponent gradient encoding, researchers can introduce locally degradable domains, mechanical gradients, bioactive patterns, and stress-distribution features within hydrogel constructs [122]. When combined with 4D printing, hydrogel structures can be designed to evolve over time through swelling, degradation, shape transformation, or external stimulus-triggered remodeling. These methods provide new opportunities for mimicking developmental morphogenesis, constructing dynamic tissue interfaces, and designing stage-specific release systems [123]. In parallel, computational design and data-driven optimization are promoting a shift toward inverse design, in which desired biological functions or disease microenvironmental requirements are first defined and then translated into material formulations, printing conditions, or exposure parameters [124]. This direction is particularly relevant to improving reproducibility and scalability of complex hydrogel constructs.

#### Biohybrid manufacturing

Biohybrid manufacturing incorporates living cells, microorganisms, biological actuators, or synthetic biology modules into hydrogel fabrication processes, allowing materials to acquire active functions derived from living systems. By coupling cell contractility with programmed hydrogel architectures, biohybrid systems capable of active deformation or contraction can be constructed for dynamic tissue models and biomedical devices [125, 126]. The ability of cells to secrete ECM can also be used to gradually biologize artificial hydrogels after fabrication, supporting the transition from synthetic scaffolds to tissue-like structures. In addition, the integration of engineered immune cells or programmable cellular modules into hydrogels may enable internal biological response units with immunoregulatory and feedback functions, allowing hydrogel systems to participate more actively in tissue development and disease modulation [127].

Overall, emerging hydrogel manufacturing methods address key limitations of conventional fabrication approaches in structural resolution, functional complexity, microenvironmental mimicry, and personalized design. These methods operate in close synergy with polymer chemistry, crosslinking design, mechanical tuning, degradation control, and functionalization strategies, enabling hydrogels to construct complex biological systems, recapitulate disease models, and support precision therapeutic interventions. At the same time, manufacturing-related challenges, including batch reproducibility, scale-up, sterilization, storage stability, regulatory classification, and cost-effectiveness, remain major issues for clinical translation. With continued development of digital, intelligent, and biointegrated manufacturing paradigms, hydrogels are expected to evolve from simple materials toward integrated biomedical systems capable of coordinating cellular behavior, biomechanical cues, immune responses, and disease progression.

## Current biomedical applications

Building on the design principles and material engineering strategies discussed above, the biomedical use of hydrogels can be organized into several major application platforms rather than isolated material examples. As summarized in Fig. 3, hydrogel functions are closely linked to their polymer chemistry, crosslinking mechanisms, mechanical and degradation properties, stimuli-responsive behavior, bioactivity, and fabrication methods. These design features enable hydrogels to support diverse biomedical applications, including drug delivery, tissue engineering and regenerative medicine, biosensing and diagnostics, and cell culture and organoid systems. More importantly, these platforms provide the material basis for disease-oriented therapeutic strategies in diverse clinical contexts, such as wound repair, musculoskeletal disorders, cancer, neurological diseases, cardiovascular diseases, autoimmune disorders, and reproductive system diseases. Therefore, this section summarizes the major biomedical application platforms of hydrogels, with an emphasis on how general material design principles are translated into functional biomedical systems.

### Hydrogel-based drug delivery systems

Current therapeutic strategies are progressively shifting from systemic administration toward tissue-specific delivery, lesion-site microenvironmental intervention, and multimodal precision therapy. Accordingly, the role of hydrogels in drug delivery has evolved from simple drug carriers into designable and controllable local therapeutic systems [128]. In hydrogel-based drug delivery, therapeutic outcomes are determined not only by the pharmacological activity of the loaded agents, but also by how the material platform controls drug retention, diffusion, protection, release kinetics, and interaction with diseased tissues. Therefore, hydrogel drug delivery should be understood as a materials construction process driven by medical requirements, in which polymer composition, crosslinking mode, mesh size, degradability, and functional modification collectively determine delivery performance [128–131].

From a materials perspective, hydrogels provide a highly adaptable network for loading different classes of therapeutics. Hydrophilic small molecules can be physically entrapped within the hydrated network, whereas hydrophobic drugs often require hydrophobic microdomains, nanoparticle hybridization, or supramolecular interactions for stable incorporation. Proteins, peptides, nucleic acids, and extracellular vesicles can be protected through electrostatic interactions, host–guest recognition, affinity binding, or dynamic covalent interactions, reducing premature deactivation and improving local retention [132, 133]. Crosslinking structure further regulates release behavior: stable covalent networks are suitable for long-term sustained delivery, physically crosslinked systems support injectability and reversible release, and dynamic covalent networks allow release profiles to be adjusted in response to environmental changes [134, 135]. By tuning polymer concentration, crosslinking density, or photopolymerization conditions, hydrogel mesh size and permeability can be controlled, thereby making diffusion kinetics more predictable and supporting temporally programmed drug delivery [136].

Stimulus-responsive release represents one of the most important advantages of hydrogel-based delivery systems. Many diseased tissues are characterized by abnormal acidity, ROS accumulation, enzyme overexpression, altered ion concentrations, or other pathological cues [137, 138]. Based on the responsive design strategies introduced in Sect. 2, hydrogels can incorporate pH-sensitive linkages, redox-sensitive bonds, matrix metalloproteinase-sensitive peptide sequences, or metal-ion coordination motifs to translate pathological signals into localized drug release [139, 140]. For example, hydrazone-crosslinked networks can degrade in acidic tumor microenvironments; ROS-responsive boronate ester or disulfide-containing hydrogels can release anti-inflammatory or antioxidant agents in oxidative lesions; and MMP-sensitive hydrogels can selectively remodel at invasive tumor fronts or tissue remodeling sites [141]. In this way, hydrogels enable drug release to be spatially confined and temporally coordinated with disease activity, rather than relying solely on passive diffusion.

In addition to regulating release kinetics, hydrogels can actively participate in the therapeutic microenvironment. By incorporating ECM-mimetic motifs such as RGD or IKVAV, immunomodulatory molecules, or cell-interactive components, hydrogel delivery systems can regulate cell adhesion, immune responses, matrix remodeling, and tissue repair while simultaneously releasing therapeutic agents [142, 143]. This feature is particularly valuable in complex disease settings such as chronic wounds, bone defects, myocardial infarction, cancer, and neural injury, where local therapy requires not only drug exposure but also microenvironmental reconstruction [144–147]. Overall, hydrogel-based drug delivery has evolved from passive drug storage toward integrated therapeutic platforms that combine controlled release, lesion responsiveness, biological protection, and microenvironmental regulation [61, 128, ,130 ].

### Tissue engineering and regenerative medicine

In tissue engineering and regenerative medicine, hydrogels have progressed from soft fillers or supportive scaffolds to key platforms for constructing regenerative microenvironments. The goal of regenerative medicine is not merely to replace damaged tissue, but to restore tissue structure, biological signaling, functional coordination, and integration with surrounding host tissue [148, 149]. Hydrogels are well suited for this purpose because their hydrated and tunable networks can mimic selected features of native ECM, provide three-dimensional support for cells, and integrate biochemical and biophysical cues required for tissue repair [150, 151].

A major function of hydrogels in tissue engineering is to provide a three-dimensional microenvironment that supports cell adhesion, survival, migration, proliferation, and differentiation [152, 153]. Through regulation of polymer composition, crosslinking density, swelling behavior, and bioactive ligand presentation, hydrogels can be adapted to different tissue requirements. Soft hydrogels can support neural or myocardial tissues, whereas tougher and mechanically reinforced systems are more suitable for cartilage, tendon, or osteochondral repair [154]. Dynamic crosslinking and stress-relaxing networks further allow hydrogels to respond to cell-generated forces and matrix remodeling, making them more similar to native ECM than static elastic scaffolds [155].

These properties are particularly important for stem cell engineering. Stem cell fate is regulated not only by soluble biochemical factors but also by mechanical cues such as stiffness, viscoelasticity, and stress relaxation [156, 157]. Hydrogels with defined mechanical properties can therefore guide lineage commitment and tissue-specific differentiation. For example, rapidly relaxing networks may favor chondrogenic matrix deposition, whereas stiffer or slower-relaxing matrices may support osteogenic differentiation in appropriate contexts [154, 158]. In this sense, hydrogels serve as instructive matrices that regulate cell behavior through both biochemical and mechanical signals.

As regenerative medicine moves toward complex and disease-associated tissue damage, hydrogel design has shifted from tissue replacement toward microenvironment reconstruction. In myocardial infarction, hydrogels can provide mechanical support while modulating inflammation, angiogenesis, metabolism, and electrical conduction-related repair processes [146, 159]. In fibrosis and tumor desmoplasia models, mechanically tunable hydrogels can reproduce ECM stiffening and abnormal cell–matrix interactions, providing platforms for disease modeling and drug screening [160]. In organoid-related regenerative systems, hydrogel matrices regulate stem cell self-organization, tissue compartmentalization, and developmental trajectories, highlighting their role as active regulators of tissue formation rather than passive culture substrates [161]. Therefore, hydrogel-based tissue engineering platforms provide a general material foundation for tissue repair strategies further discussed in disease-specific sections.

### Biosensing and diagnostic systems

The development of modern diagnostic technologies is moving toward real-time monitoring, minimal invasiveness, multiparameter analysis, and improved ability to capture disease microenvironment changes. Hydrogels have become important materials for biosensing and diagnostic systems because their hydrated networks, tunable mechanics, environmental responsiveness, and tissue compatibility allow them to interface effectively with biological systems [162–164]. Compared with rigid sensing materials, hydrogel-based sensors can better match soft tissues, reduce mechanical irritation, and support long-term or continuous monitoring [165].

Hydrogels can integrate recognition elements, signal transduction modules, and functional nanomaterials within a single soft network [166, 167]. Their water-rich structure provides physiologically relevant diffusion pathways for ions, metabolites, biomacromolecules, and signaling molecules, while the programmability of polymer architecture, crosslinking density, mesh size, and interfacial chemistry allows control over sensitivity, selectivity, stability, and signal amplification [168, 169]. Therefore, hydrogels can function not merely as soft carriers for sensing units, but also as active components of bioanalytical systems.

At the signal recognition level, hydrogels can convert disease-related cues such as pH, ionic strength, redox state, enzyme activity, and metabolites into changes in swelling, degradation, conductivity, fluorescence, color, or mechanical properties [170]. These material responses can be coupled with optical, electrochemical, resistive, or mechanical readouts to quantify target analytes. When combined with conductive polymers, metal nanoparticles, graphene, carbon nanotubes, or other functional fillers, hydrogel sensors can achieve enhanced signal transduction for molecules such as glucose, lactate, inflammatory markers, neurotransmitters, and other disease-related biomarkers [171, 172].

Mechanical compatibility is equally important in biosensing. Many biological tissues, including skin, blood vessels, myocardium, cartilage, and brain tissue, undergo continuous deformation or micromotion. Rigid sensors may cause tissue damage, interfacial mismatch, and signal drift, whereas hydrogels with tunable modulus and adaptive deformation can maintain more stable contact with soft tissues [165, 173]. Dynamic crosslinked hydrogels with stress relaxation or self-healing behavior can further reduce interfacial failure during long-term monitoring [174, 175]. By embedding piezoresistive, piezoelectric, capacitive, or ionic conductive elements within hydrogel networks, mechanically responsive hydrogel sensors can monitor vascular wall tension, muscle contraction, tissue repair, respiration, heartbeat, and other physiological activities [176–178].

Beyond direct biosensing, hydrogels can also support functional diagnostics through disease microenvironment reconstruction. ECM-mimetic and mechanically tunable hydrogels can model tumor stroma, inflammatory tissues, bone marrow niches, ischemic states, or infected microenvironments, thereby enabling functional evaluation of disease behavior and therapeutic response [179]. Such hydrogel-based diagnostic platforms shift the focus from single-biomarker detection toward functional state profiling, which may provide more biologically relevant and clinically predictive information [161, 179, 180].

### Cell culture and organoid models

With the rapid development of organoids, advanced cell culture systems, and disease models, hydrogels have become important three-dimensional platforms for reconstructing artificial microenvironments. In these systems, hydrogels are not simply scaffolds for cell growth, but materials that regulate cell fate, tissue self-organization, morphogenesis, and disease phenotype through polymer composition, crosslinking structure, mechanical properties, degradation behavior, ligand presentation, and mass transport [181–184]. Organoid formation depends on stem cell self-organization, cell–cell interactions, polarity establishment, lumen formation, tissue compartmentalization, and morphogen gradients, all of which are strongly influenced by the surrounding hydrogel microenvironment [185].

The mechanical properties of hydrogels are particularly important for organoid development. Developing tissues do not grow in static elastic environments; rather, they experience viscoelasticity, stress relaxation, shear softening, and spatial heterogeneity [186]. By adjusting polymer chain flexibility, crosslinking density, mesh size, or dynamic bond chemistry, hydrogels can provide time-dependent mechanical responses that allow tissue expansion, cell rearrangement, and local network remodeling [102, 187]. Hydrogels with rapid stress relaxation can promote organoid growth, branching morphogenesis, and lineage specification by reducing mechanical resistance at the cell–matrix interface [186]. In addition, stiffness gradients, patterned microstructures, curved architectures, and programmable topologies can influence symmetry, compartmentalization, and tissue patterning during organoid formation [188].

Biochemical signal presentation is another key determinant of hydrogel-based organoid systems. Organoid development depends on integrin signaling, cell–matrix adhesion, cell–cell feedback, and localized morphogen cues. Therefore, the type, density, and distribution of ligands such as RGD, IKVAV, and YIGSR, as well as the incorporation of degradable peptide motifs, directly influence stem cell dynamics and tissue architecture [189, 190]. In intestinal organoids, appropriate matrix degradability supports stem cell niche expansion and crypt-like structure formation, whereas in brain organoids, reduced matrix adhesion and enhanced cell aggregation can facilitate neuroectodermal organization and regional development [185, 191–193]. Hydrogels can also be engineered to present gradients of morphogens such as Wnt, BMP, and FGF, allowing spatial signaling patterns to be reconstructed in vitro [194].

Mass transport within hydrogels further affects organoid viability and maturation. Pore size, interconnectivity, diffusivity, and molecular sieving properties influence the distribution of nutrients, oxygen, metabolites, and paracrine factors [195]. Excessively dense networks may restrict diffusion and induce hypoxia or necrosis, whereas overly loose networks may fail to retain local signals. By tuning polymer concentration, crosslinking conditions, photopolymerization intensity, and microstructural design, hydrogel matrices can be optimized to support more physiologically relevant transport and signaling environments [195]. Microgel assembly and microfluidic compartmentalization further enable the construction of multi-domain hydrogels for complex organoid systems, including hepato-biliary organoids, renal filtration models, and blood–brain barrier models [196].

Overall, the central value of hydrogels in cell culture and organoid systems lies in their ability to reconstruct key microenvironmental parameters that govern tissue development, disease progression, and therapeutic response. Through the integration of polymer engineering, dynamic crosslinking chemistry, micro- and nanofabrication, and biological systems design, hydrogel-based organoid platforms are evolving from simple culture matrices toward engineered developmental systems [197, 198]. These platforms provide important tools for disease modeling, drug screening, regenerative medicine, and precision medicine, and they also complement the disease-oriented therapeutic strategies discussed in the following section.

## Hydrogel-based strategies for disease treatment and tissue repair

Hydrogels have evolved from passive hydrated matrices into multifunctional biomedical systems capable of integrating local delivery, tissue support, immune modulation, microenvironmental regulation, and disease-responsive therapeutic functions. Their high water content, tissue-mimetic properties, tunable mechanical behavior, programmable degradation, and compatibility with diverse bioactive components enable them to be tailored for different clinical requirements. In disease treatment and tissue repair, these material features are particularly valuable because many pathological conditions share common therapeutic challenges, including persistent inflammation, oxidative stress, fibrosis, immune dysregulation, impaired vascularization, insufficient tissue regeneration, and complex local microenvironments. Therefore, hydrogel-based strategies are increasingly being designed not merely as drug carriers or structural fillers, but as interactive therapeutic platforms that can respond to disease-specific cues and coordinate multiple biological processes. In the following sections, we discuss representative hydrogel-based strategies for disease treatment and tissue repair, with an emphasis on how hydrogel design principles are adapted to distinct pathological environments, therapeutic objectives, and clinical application scenarios.

### Wound repair and hemostatic management

The skin is the largest organ of the human body and serves essential physiological functions, including sensing external stimuli, regulating body temperature, and protecting against environmental insults [199]. Because of its direct exposure to the external environment, the skin is also highly susceptible to injury. Although many common skin wounds can undergo apparent closure over time, adult skin rarely achieves complete functional regeneration comparable to that observed during early developmental stages [200]. Normal cutaneous wound healing is a highly orchestrated and dynamically regulated process involving four overlapping phases: hemostasis, inflammation, proliferation, and remodeling [201]. In clinical practice, however, this process is frequently disrupted by intrinsic and extrinsic factors, such as excessive inflammation [202], extensive tissue loss caused by large-area defects [203], bacterial infection [204], and metabolic disorders such as diabetes [205], leading to delayed or chronic non-healing wounds. These conditions impose substantial physical and psychological burdens on patients and markedly increase healthcare costs.

Wound dressings are important adjuncts in wound management because they can cover wound surfaces, provide temporary protection against contamination and infection [206], and serve as instructive templates that guide cell reorganization, host tissue infiltration, and subsequent tissue integration. Conventional dressings, such as gauze and bandages, are widely used because of their absorbency and simplicity, but they require frequent replacement, may cause pain during dressing changes, and often show limited adhesiveness and poor conformability to irregular wound surfaces [207]. Therefore, an ideal wound dressing should combine good tissue compatibility, appropriate moisture retention, sufficient mechanical integrity, resistance to external bacterial invasion, and surface or biochemical cues that support cell adhesion, proliferation, and differentiation [208]. Hydrogels, owing to their high hydrophilicity, favorable biocompatibility, ECM-like porous structures, and tunable physicochemical properties, have emerged as competitive candidates for advanced wound dressing materials [209].

A wide range of natural polymers, including chitosan, gelatin, hyaluronic acid, alginate, and dextran [210], as well as synthetic polymers such as poly(ethylene glycol) (PEG), poloxamers, poly(vinyl alcohol), polyacrylamide, poly(acrylic acid), and polypeptides [211], can be used to construct hydrogel networks through chemical or physical crosslinking. Engineering strategies such as dynamic covalent bonding, photo-crosslinked in situ polymerization, double-network architectures, semi-interpenetrating networks, and three-dimensional printing have further expanded the functional design space of hydrogel dressings [212]. Accordingly, hydrogel-based wound repair strategies have evolved from simple moist dressings toward multifunctional systems that integrate antibacterial activity, immune and oxidative stress regulation, angiogenesis promotion, tissue adhesion, hemostasis, and responsive drug release (Table 1).

**Table 1:** Representative hydrogel-based strategies for wound repair and hemostatic management

Diseases	Biomaterial	Active substances	Advantages	References
Bacteria-infected burn wounds	Pluronic F-127	Cu^2+^, Zn^2+^	First integration of PZDT and CDT; Cu/Zn doping enhances piezoelectricity and catalysis; Self-powered immunomodulation (motion-induced voltage + Zn^2+^); Triple-action: sterilization, anti-inflammation, and tissue regeneration	[213]
MRSA-infected wound	F127 hydrogel	Cu_1.5_Mn_1.5_O_4_ nanospheres	Enhanced multi-enzyme antibacterial activity; Synergistic photothermal sterilization; Blocks bacterial protein and nucleotide metabolism; Thermo-responsive rapid hemostasis; Sustained Cu/Mn release promotes healing	[214]
	DNA hydrogel (composed of linear DNA strands (Linker) and Y-shaped DNA building units)	Ginseng-derived exosomes (G-Exos), L-Arginine (L-Arg, a nitric oxide precursor), aggregation-induced emission luminogen (AIEgen, specifically TPA-COOH)	ROS-responsive, light-triggered gas therapy, synergistic antibacterial, immunomodulatory	[215]
	Carboxymethyl chitosan; Oxidized sodium alginate	Artificial multienzyme nanoflowers (composed of glucose oxidase and hemoglobin) and hydroxyurea (as a nitric oxide donor)	Glucose-responsive nitric oxide release and multifaceted microenvironment regulation	[216]
	Carboxymethyl chitosan/oxidized sodium alginate composite network	Glucose oxidase-hemoglobin artificial multienzyme nanoflower + hydroxyurea (nitric oxide donor)	Glucose-triggered nitric oxide release for integrated antibacterial and pro-healing	[217]
	Silk protein methacryloyl hydrogel	Broccoli-derived exosomes, epigallocatechin gallate-gold nanoparticles	Photothermal antibacterial, anti-inflammatory and anti-scarring, pro-angiogenic	[218]
Bacterium-infected diabetic wounds	DNA/PPy hydrogels	DNA-templated silver nanoclusters; Diclofenac sodium; Vascular Endothelial Growth Factor	Triboelectro-responsive DNA hydrogel integrated with TENG remodels wound electric field for electrically controlled release of antibacterial, anti-inflammatory, and pro-angiogenic agents, synergistically accelerating healing of diabetic infected wounds	[219]
	PAM–(Alg + VEGF)–PDA–Ag hydrogel	pH-responsive microcarriers	pH-triggered sequential release of AgNPs and VEGF accelerates infected wound healing with 83% closure at day 7	[220]
Cavitary traumatic wound	DNA hydrogel (DNAgel) prepared by covalently cross-linking salmon sperm DNA with poly(ethylene glycol) diacrylate (PEGDA)	/	Rapid swelling, strong wet tissue adhesion, biomimetic coagulation, superior hemostasis to gelatin sponge, and promotion of wound healing	[221]
	HSA-4aPEG-OPA hydrogel	bFGF-liposomes	Cutaneous trauma treated with HSA-4aPEG-OPA hydrogel carrying bFGF-liposomes: suture-free, strong adhesion, fast healing, minimal scar	[222]
	Sodium alginate; Carboxymethyl chitosan; Ethyl lauroyl arginate hydrochloride	Norepinephrine	This injectable foam hydrogel is characterized by convenient application, rapid hemostasis (effective even in cases of coagulopathy), and safe post-use removal via urea dissolution and negative pressure drainage	[223]
	GelMA/HA-CD-BP composite network	Resveratrol (RES)	Blood-absorbing weight gain for self-compression hemostasis and inflammation relief	[224]
Diabetic ulcers	Dopamine-modified gelatin, phenylboronic acid-modified hyaluronic acid	Copper-loaded polydopamine nanoparticles, metformin	Photothermal antibacterial, microenvironment-responsive, pro-angiogenic	[225]
	Ellagic acid-copper metal–organic-framework nanozyme	Loaded agent: ellagic acid-copper ion self-assembled complex	Antibacterial, anti-inflammatory, pro-repair	[226]
	Polyvinyl alcohol crosslinked with phenylboronic ester	Dimeric copper peptide	Self-healing ROS-scavenging	[227]
Chronic non-healing full-thickness diabetic skin wounds	Biosynthesized silk sericin from transgenic silkworm	Epidermal growth factor and platelet-derived growth factor-BB	Dual-GF synergy heals in 12 days with low inflammation, rich vascularization, and ordered collagen	[228]
Methicillin-resistant *Staphylococcus aureus*-infected hypertrophic scar	Polyvinyl alcohol-agarose double network	Hyperbranched polylysine and tannic acid	Antibacterial anti-scar in one	[229]

#### Infected and diabetic wounds

Infected wounds, including burn-associated infections, post-traumatic infections, and diabetic foot ulcers, represent a major challenge in clinical wound management. Bacterial colonization can establish a pathogenic wound microenvironment, promote biofilm formation, and trigger persistent inflammation, thereby preventing wounds from progressing from the inflammatory phase to the proliferative and remodeling phases. Although antibiotics remain important in infection control, their long-term efficacy is increasingly limited by antibiotic resistance, especially in infections involving methicillin-resistant *Staphylococcus aureus* (MRSA) [230, 231]. Therefore, hydrogel-based strategies for infected wounds are increasingly designed not only to inhibit bacterial growth, but also to reshape the local microenvironment to support subsequent tissue repair.

A key design logic for infected wound hydrogels is the regulation of the inflammatory and oxidative microenvironment. Pathological wounds are often characterized by sustained release of pro-inflammatory cytokines, aberrant activation of immune cells, and excessive ROS accumulation [232, 233]. Although moderate ROS levels contribute to bacterial killing and wound signaling, persistent ROS accumulation aggravates oxidative stress, induces cell apoptosis, and impairs fibroblast, keratinocyte, and endothelial cell function. Accordingly, several hydrogel systems have been designed to regulate ROS generation or scavenging in a spatiotemporally controlled manner. For example, Roy et al. developed a piezoelectric dynamic therapy hydrogel in which Cu/Zn co-doping and atomic-order engineering enhanced ultrasound-induced polarization and ROS production. This system disrupted drug-resistant bacteria and biofilms, reduced inflammatory cytokine levels, suppressed neutrophil infiltration, promoted macrophage polarization from the M1 to M2 phenotype, and enhanced angiogenesis and collagen deposition in infected wound models [213]. Similarly, Ye et al. constructed a ROS-responsive DNA hydrogel incorporating aggregation-induced emission luminogens, ginseng-derived exosomes, and L-arginine. Under laser irradiation, this system generated superoxide radicals and nitric oxide, producing synergistic antibacterial and pro-angiogenic effects while reducing inflammatory gene expression in *S. aureus*-infected wounds [215]. Shi et al. further designed a thermosensitive Cu_1.5_Mn_1.5_O_4_/F127 hydrogel with multienzyme-like and photothermal activities, which combined ROS generation, glutathione depletion, ion release, and bacterial metabolic interference to achieve antibacterial activity and microenvironment regulation [214].

Beyond ROS regulation, wound repair is also influenced by local bioelectrical signals and angiogenic cues. Bacterial infection can disrupt endogenous electric fields and thereby impair cell migration and tissue regeneration. To address this issue, Guo et al. developed a triboelectric-responsive DNA hydrogel that combined conductive polymers, functional DNA sequences, and triboelectric stimulation to regulate bioelectrical behavior, enhance antibacterial activity, and promote collagen deposition, angiogenesis, and hair follicle regeneration in infected diabetic wounds [219]. In addition, because impaired angiogenesis is a major barrier to infected chronic wound repair, Xie et al. developed a pH-responsive VEGF-loaded calcium alginate microcarrier system with a polydopamine coating and immobilized AgNPs. This design improved tissue adhesiveness, controlled VEGF release, provided antibacterial activity, upregulated CD31 expression, and reduced IL-6 expression, thereby integrating infection control with angiogenesis promotion [220].

Another important direction is the development of non-antibiotic antibacterial hydrogels that simultaneously modulate immune responses. Macrophage phenotypic transition plays a central role in wound healing: excessive M1 activation promotes TNF-α and IL-6 secretion and aggravates tissue damage, whereas timely M2 polarization supports resolution of inflammation and tissue regeneration through mediators such as TGF-β and IL-10 [234, 235]. Under persistent inflammation driven by drug-resistant bacterial infection, precise regulation of macrophage polarization has therefore become an important therapeutic target [236, 237]. Representative systems include a silk fibroin-based hydrogel incorporating broccoli-derived exosomes and EGCG-gold nanoparticles, which combined anti-infective and immunoregenerative effects to promote scarless healing in MRSA-infected wounds [218]. Liu et al. developed a high-strength PVA/agarose double-network hydrogel containing hyperbranched polylysine and tannic acid, thereby integrating mechanical robustness, antibacterial activity, antioxidant effects, and anti-scarring performance in MRSA-infected wound and hypertrophic scar models [229]. In addition, Zhang et al. reported an in situ-forming antibiotic-free OPTH hydrogel based on octa-arm PEG amine and tetrakis(hydroxymethyl)phosphonium chloride, a cationic phosphonium compound with broad antimicrobial activity and degradability [238]. This hydrogel showed rapid gelation, injectability, broad-spectrum antibacterial activity against *E. coli*, *S. aureus*, and MRSA, and preserved the structural integrity of iPSC-derived three-dimensional human skin organoids while reducing pro-inflammatory cytokine expression [238].

Diabetic wounds represent a particularly complex subtype of chronic infected wounds. Diabetes-associated vascular injury, neuropathy, and immune dysfunction reduce the regenerative capacity of skin and soft tissues and increase susceptibility to secondary infection by pathogens such as *S. aureus* and *Pseudomonas aeruginosa* [239]. The diabetic wound microenvironment is typically characterized by hyperglycemia, elevated pH, hypoxia, persistent inflammation, oxidative stress imbalance, and insufficient angiogenesis. Because glucose oxidase can catalyze glucose oxidation to generate gluconic acid and H_2_O_2_, GOx-based or GOx-like hydrogel systems have been developed to regulate local glucose levels and pH [240, 241]. To improve enzymatic stability, Ma et al. constructed an artificial multienzyme nanoflower-based composite hydrogel composed of GOx, hemoglobin, and a carboxymethyl chitosan/oxidized sodium alginate matrix. This system exhibited antibacterial activity, inhibited dense biofilm formation, promoted endothelial cell migration, and accelerated diabetic wound closure [216].

In addition to glucose and pH regulation, diabetic wound healing requires coordinated control of inflammation, oxidative stress, immune cell behavior, and angiogenesis [242, 243]. Zhang et al. developed a dynamically crosslinked hydrogel film based on four-arm PEG, tannic acid, and a β-peptide polymer, integrating antibacterial activity, ROS scavenging, exudate management, mechanical strength, and hemostatic capability. In an MRSA-infected diabetic wound model, this film reduced bacterial burden, controlled exudate accumulation, and accelerated wound healing [217]. Zhu et al. further reported a pH- and glucose-responsive hydrogel loaded with metformin and CuPDA nanoparticles, which was constructed through dynamic phenylboronate ester crosslinking among dopamine-modified gelatin, CuPDA nanoparticles, and phenylboronic acid-modified hyaluronic acid. This hydrogel combined antibacterial activity, inflammation suppression, angiogenesis promotion, and ECM/collagen deposition to improve infected diabetic wound repair [225]. More recently, Cong et al. developed a ROS-responsive hydrogel incorporating a dimeric copper peptide. This platform provided anti-inflammatory, antioxidant, pro-angiogenic, and protease-resistant activities, while allowing controlled release in the oxidative wound microenvironment. In a diabetic mouse model, the hydrogel markedly reduced residual wound area and promoted regeneration of skin appendages, highlighting the value of multifunctional hydrogels for complex diabetic wound repair [227, 244, 245].

Overall, infected and diabetic wounds illustrate how hydrogel design has shifted from passive wound coverage to active microenvironment regulation. By integrating antibacterial activity, ROS modulation, immune regulation, bioelectrical stimulation, angiogenesis promotion, and responsive drug release, hydrogel systems can address multiple pathological features of chronic wounds within a single local therapeutic platform.

#### Traumatic wounds and hemostasis

Traumatic wounds caused by surgery, accidents, firearms, weapons, or explosions can lead to severe bleeding, particularly when critical organs or large vascular networks are involved. Without timely intervention, uncontrolled hemorrhage may rapidly progress to hemorrhagic shock, organ failure, or death [246]. Conventional hemostatic methods, including suturing, tourniquet application, and compression, are often limited by poor accessibility, large or irregular wound areas, and difficulty in directly compressing deep bleeding sites [247]. Therefore, hydrogel-based hemostatic materials have attracted attention because they can combine rapid fluid absorption, tissue adhesion, injectability, mechanical reinforcement, procoagulant activity, and support for subsequent tissue repair.

For external traumatic wounds, effective materials should rapidly stop bleeding while supporting later wound healing and cosmetic recovery [248, 249]. One representative approach is to use hydrogel networks that physically enrich blood cells and platelets to accelerate clot formation. Ye et al. constructed a natural DNA hydrogel using DNA extracted from salmon sperm. The resulting DNAgel showed favorable liquid absorption, tissue adhesion, and the ability to absorb plasma and blood cells. In multiple bleeding models, including rat tail amputation, femoral artery injury, and liver puncture injury, DNAgel markedly reduced blood loss compared with controls, indicating the potential of DNA-based hydrogel networks for rapid hemostasis [221]. In addition to blood absorption and clot stabilization, growth factor delivery can further support tissue repair after traumatic injury. Geng et al. developed a hydrogel tissue adhesive formed by an irreversible OPA-amine condensation reaction between human serum albumin and ortho-phthalaldehyde-functionalized four-arm PEG. By incorporating bFGF-loaded liposomes, this system enabled sustained growth factor release, promoted cell proliferation, collagen deposition, and angiogenesis, and achieved suture-free wound closure in rat and porcine full-thickness incision models [222].

For large-area bleeding wounds, hydrogel dressings can also integrate mechanical compression, self-reinforcement, and anti-inflammatory functions. Tan et al. developed a composite HG-CB@R hydrogel dressing based on GelMA, bisphosphonate, cyclodextrin-modified hyaluronic acid, and resveratrol. After application, the hydrogel enriched local blood cells and platelets through electrostatic interactions, absorbed fluid and generated local compression, and underwent iron ion-mediated self-reinforcement to reduce the risk of gel rupture and secondary bleeding. The sustained release of resveratrol further suppressed inflammation, promoted fibroblast migration, and enhanced collagen deposition, thereby improving both hemostatic efficiency and healing quality in acute bleeding wounds [224].

Intra-cavity traumatic wounds, such as abdominal or thoracic injuries, present additional challenges because bleeding sites are often difficult to visualize or compress directly, and conventional suturing or bandaging is frequently impractical. Deep abdominal injuries therefore require hemostatic systems that are rapid, convenient, injectable, and adaptable to irregular wound geometries [250]. Injectable foam systems are particularly attractive because they can expand after administration, provide physical tamponade, enrich coagulation factors, and conform to deep or irregular wound spaces [251]. Based on this concept, Guo et al. developed an injectable hemostatic foam in which reversible hydrogen bonding, norepinephrine-mediated vasoconstriction, and a porous foam architecture acted synergistically to control intra-abdominal bleeding. In rat, rabbit, and pig hemorrhage models, this system achieved rapid hemostasis even under heparin-induced coagulopathy, demonstrating its potential for emergency hemorrhage control [223].

Other hydrogel systems for intra-cavity hemostasis rely on strong tissue adhesion and rapid in situ gelation. Biomass-based supramolecular hydrogels, for example, exhibit favorable biocompatibility, tissue adhesiveness, and mechanical performance [252]. Wang et al. fabricated an injectable alginate supramolecular hydrogel through nonsolvent-induced self-assembly of alginate nanofibers. This hydrogel formed a continuous nanofibrous network, adhered strongly to porcine skin under deformation, and reduced blood loss to approximately 0.1 g within 1 min in a rabbit liver injury model, with bleeding largely controlled within 5 min [253]. For emergency use, hydrogel sealants should also combine rapid gelation, mechanical stability, procoagulant activity, and practical handling properties [254]. Shi et al. developed an injectable and dissolvable hydrogel sealant based on Schiff base chemistry between a four-arm PEG aldehyde crosslinker containing thioester linkages and mixed amine components. This system rapidly formed adhesive gels in vivo, promoted local hemostasis without direct manual compression, and could be dissolved on demand through thiol-thioester exchange. In femoral artery puncture and liver injury models, the hydrogel significantly reduced blood loss, supporting its translational potential for intra-cavity traumatic hemostasis [255].

Taken together, hydrogel-based strategies for traumatic wounds have evolved beyond simple wound coverage toward multifunctional hemostatic platforms. For external wounds, hydrogels mainly act by absorbing blood, concentrating platelets, adhering to tissue, delivering repair-promoting factors, and improving later tissue reconstruction. For intra-cavity injuries, injectable foams, supramolecular hydrogels, and dissolvable sealants provide rapid space filling, tissue adhesion, physical tamponade, and controlled removal when needed. These features make hydrogels attractive candidates for trauma management.

Overall, hydrogels demonstrate distinctive advantages in wound repair and hemostatic management owing to their high water content, biocompatibility, tunable mechanical properties, tissue adhesiveness, and flexible functionalization capacity. In infected and diabetic wounds, hydrogels can combine antibacterial activity, immune modulation, ROS regulation, angiogenesis promotion, and responsive drug release to intervene in complex chronic wound microenvironments. In traumatic wounds, hydrogels can integrate rapid hemostasis, tissue sealing, mechanical support, and subsequent repair-promoting functions. By coordinating local microenvironment regulation with structural and therapeutic support, hydrogel-based systems provide versatile material platforms for improving wound healing outcomes across diverse clinical scenarios.

### Musculoskeletal tissue repair

Musculoskeletal disorders are commonly associated with persistent pain, restricted mobility, and functional impairment, and their progression is often accompanied by structural tissue damage, mechanical microenvironment imbalance, chronic inflammation, insufficient vascularization, and limited intrinsic regenerative capacity [256, 257]. These pathological features make musculoskeletal repair a highly dynamic and multistage process, particularly in tissues such as bone, cartilage, meniscus, and tendon, where restoration of both structural integrity and mechanical function is required.

Given the load-bearing and mechanically active nature of the musculoskeletal system, successful repair requires coordinated regulation of mechanical support, cellular recruitment, vascularization, immune responses, ECM deposition, and tissue remodeling [258]. Conventional inert implants or single-function materials often provide structural support but show limited capacity to regulate the temporal sequence of repair. Therefore, hydrogel-based strategies for musculoskeletal repair are increasingly designed to match stage-specific therapeutic needs, including defect stabilization, osteogenic or chondrogenic induction, inflammation modulation, vascular ingrowth, and interface reconstruction [259]. The following sections summarize representative hydrogel-based strategies for bone defects, cartilage injury and osteoarthritis, and tendon repair (Table 2).

**Table 2:** Representative hydrogel-based strategies for musculoskeletal tissue repair

Diseases	Biomaterial	Active substances	Advantages	References
Meniscus repair	Gelatin phenylboronic acid; Polyvinyl alcohol; Chondroitin sulfate methacryloyl	Naproxen; Transforming growth factor-beta3; Connective tissue growth factor	Temporally regulating immune microenvironment	[134]
	Injectable decellularized Wharton’s jelly hydrogel	CD56 umbilical cord mesenchymal stem cell-derived exosomes	Sustained release promotes repair	[260]
	Gelatin methacryloyl	No drug loaded, studying the cells themselves	Mimicking physiological tensile loading to maintain homeostasis	[261]
	Gelatin Methacryloyl/Hyaluronic Acid Methacryloyl hydrogel	Transforming Growth Factor-β3; Connective Tissue Growth Factor	Zone-mimetic anisotropy	[262]
Osteoarthritis	Composed of electrospun poly-L-lactic acid short nanofibers and collagen matrix	/	Injectable, biodegradable, self-powered, non-invasive activation	[263]
	Hyaluronic acid-hydrazide/hyaluronic acid–aldehyde dynamic crosslinked network	ε-polylysine-modified manganese cobalt oxide nanozyme	Oxygenating bone protection	[264]
	Poly(organophosphazene)	Triamcinolone acetonide	Sustained release, localized treatment, good biocompatibility	[265]
Osteochondral defects	Gelatin methacryloyl and peptide-based rigid nanorods	/	Injectable, high strength and toughness, compatible with additive manufacturing	[266]
	Methacrylated gelatin, hyaluronic acid-graft-dopamine, oxidized hyaluronic acid	Human adipose-derived mesenchymal stem cell-derived exosomes	Bilayer biomimetic microenvironment, sustained exosome release	[267]
	Silk fibroin-deoxyribonucleic acid dual-network hydrogel microsphere	Peptide-arginine-glycine-aspartic acid-phenylalanine-lysine-alanine-cysteine	Boosts cartilage organoid formation	[268]
Tendon repair	Ultralong hydroxyapatite nanowires/gelatin-hyaluronic acid composite hydrogel	Quercetin	Synergistic anti-aging and structural guidance promote repair	[269]
	GelMA/oxidized alginate interpenetrating-network hydrogel	Urea-extracted tendon ECM	Transcriptome-optimized, precise tenogenesis	[270]
	Thiolated gelatin hydrogel	/	Provides a stratified microenvironment supporting fibrochondrogenic differentiation	[271]
Bone injury repair	Gelatin methacryloyl	Bisphosphonate; Magnesium ions	Active capture and sustained release, promotes osteogenesis and inhibits osteoclasts	[272]
	Gelatin-calcium phosphate cement-laponite composite hydrogel	Silicon, magnesium, lithium ions released from degrading laponite	Degradation matches bone regeneration	[273]

#### Bone defect repair and osteogenic reconstruction

Bone defects, particularly critical-sized defects in load-bearing regions caused by severe trauma, tumor resection, or infection, represent one of the most challenging conditions in musculoskeletal repair. Unlike simple fractures, the repair of large bone defects depends on a coordinated process involving defect stabilization, inflammatory microenvironment regulation, angiogenesis, osteogenic differentiation, and subsequent bone remodeling [274, 275]. Therefore, hydrogel-based strategies for bone repair must simultaneously address mechanical support, osteogenic induction, vascularization, and degradation matching.

Mechanical performance remains a central challenge for hydrogels in bone defect repair. Although the high water content of hydrogels facilitates ECM mimicry, it also results in relatively low elastic modulus and fatigue resistance compared with native bone, especially in load-bearing or irregular defect sites where stress concentration may cause material collapse or structural failure [256]. Early approaches mainly attempted to strengthen hydrogels by increasing crosslinking density or incorporating rigid components, but excessive stiffening may compromise mechanical compatibility with surrounding tissues. More recent designs have therefore shifted toward synergistically improving strength, toughness, and shape adaptability. Double-network or multinetwork hydrogels, formed by combining a brittle energy-dissipating network with a ductile extensible network, can improve fracture resistance and structural stability while maintaining softness and injectability [276, 277]. Nanoreinforcement further expands this strategy. For example, peptide-based rigid nanorod-reinforced GelMA hydrogels can dissipate mechanical energy through reversible deformation and orientation rearrangement of nanostructures under loading, thereby maintaining structural integrity under repetitive stress while preserving injectability and in situ photocrosslinking capacity [266].

However, mechanical stability alone is insufficient for effective bone regeneration. Without osteoinductive signals, the repair process may remain fibrotic or result in incomplete mineralization. Accordingly, hydrogel-based bone repair has increasingly moved from single growth factor delivery toward multimodal osteoinductive regulation. Stem cell-derived exosomes have attracted particular attention because they contain multiple osteogenic signals and can be locally retained and released through adhesive and degradable hydrogel matrices [278]. Such systems can enhance new bone formation and mineralization quality while reducing risks associated with high-dose growth factor administration, including ectopic ossification and excessive inflammatory responses [279].

Vascularization is another critical bottleneck in large-volume bone defect repair. Bone regeneration requires adequate blood supply to support oxygen and nutrient transport and to remove metabolic waste [280, 281]. Porous structures alone are often insufficient to establish functional vascular networks at early stages; thus, hydrogel designs have increasingly emphasized coordinated osteogenesis and angiogenesis. Pro-angiogenic factors or angiogenesis-related extracellular vesicles can be incorporated into hydrogels to promote endothelial migration and tubulogenesis, while the mechanical microenvironment, degradation kinetics, and immunomodulatory properties of hydrogels also influence vascular ingrowth [282, 283]. Injectable hydrogels capable of co-delivering stem cell-derived extracellular vesicles and thymosin beta 4 further demonstrate the feasibility of coordinating bone, vascular, and neural regeneration, thereby improving the overall quality of complex bone defect repair [284].

The degradation behavior of hydrogels also strongly affects bone repair outcomes. Excessively rapid degradation may lead to premature loss of mechanical support, whereas overly slow degradation can hinder new bone ingrowth and induce chronic inflammation [256]. By introducing hydrolytically labile linkages, dynamic covalent bonds, or enzyme-sensitive peptide sequences, programmable degradation strategies allow hydrogels to adjust their degradation profiles in response to the local microenvironment. When combined with spatially heterogeneous or layered structures, these systems can provide different functional roles at different repair stages, achieving a dynamic balance among structural support, biological guidance, and tissue replacement [285].

#### Cartilage injury and osteoarthritis

Articular cartilage is a highly specialized load-bearing tissue characterized by low cellularity, absence of vasculature, and slow matrix turnover, resulting in a limited intrinsic capacity for self-repair after injury [286]. Both traumatic cartilage defects and degeneration-associated lesions face challenges such as insufficient cell sources, limited matrix synthesis, poor long-term stability under mechanical loading, and difficulty in restoring the cartilage–subchondral bone unit [287, 288]. Osteoarthritis (OA), meanwhile, is now widely regarded as a whole-joint disease rather than isolated cartilage wear, involving progressive cartilage degeneration, synovial inflammation, subchondral bone remodeling, and long-term imbalance of the joint microenvironment [289, 290]. These features make cartilage injury and OA closely related clinical scenarios in which hydrogel systems are expected not only to fill defects or deliver drugs, but also to reconstruct a dynamic and mechanically responsive joint microenvironment.

For cartilage repair, one major design requirement is mechanical matching with native cartilage. Natural articular cartilage exhibits pronounced viscoelasticity, stress relaxation, and loading history-dependent behavior, whereas hydrogels with a single static modulus often fail to maintain a stable cellular mechanical environment under repeated compression and shear [291]. Increasing evidence suggests that stress relaxation behavior may be more important than initial stiffness in regulating chondrocyte function. By incorporating dynamic and reversible crosslinking motifs, hydrogels with time-dependent mechanical responses can undergo network remodeling under sustained mechanical stimulation, reduce mechanical resistance at the cell–matrix interface, and support ECM deposition [74]. Strategies emphasizing mechanical confinement have also been shown to guide chondrocytes toward ECM architectures that more closely resemble native cartilage, indicating that successful cartilage repair materials should integrate cushioning capacity with structural remodelability [292].

Biochemical biomimicry is equally important for cartilage repair. Chondrocytes are highly sensitive to matrix composition, adhesion ligand density, and degradation behavior, whereas bioinert hydrogels often fail to sustain a stable chondrogenic phenotype over time [287]. Incorporating hyaluronic acid, chondroitin sulfate, or polysaccharide–protein composite structures into hydrogel networks can recapitulate aspects of the molecular composition and hydration characteristics of native cartilage ECM [32, 293]. At the same time, adhesion cues must be precisely regulated, as moderate integrin engagement supports cell survival and matrix synthesis, whereas excessive adhesion may promote dedifferentiation [292]. Cell-mediated degradability further allows hydrogels to gradually yield to newly deposited matrix under protease activity, thereby matching material degradation with cartilage formation and improving tissue integration [294].

In clinically relevant cartilage repair, material biomimicry alone is often insufficient, and hydrogels are increasingly used as platforms for coordinated delivery of cells, exosomes, and bioactive factors. Injectable and in situ gelling cell-laden hydrogels can enhance transplanted cell survival and modulate paracrine activity through their mechanical and degradation profiles [295, 296]. With the growing interest in cell-free strategies, exosomes have become attractive because of their low immunogenicity and relative stability, but their rapid diffusion and clearance make hydrogel carriers important for prolonging local activity [297]. For growth factor delivery, the main challenge is not simple incorporation, but safe, sustained, and localized presentation. Immobilizing or locally anchoring chondrogenic factors such as TGF-β3 within degradable hydrogel networks can provide more physiologically relevant signal exposure and promote higher-quality cartilage-like tissue formation [298]. In addition, immunomodulatory hydrogels can regulate macrophage polarization and improve the inflammatory microenvironment, thereby supporting cartilage regeneration [299, 300]. For osteochondral defects, multilayered or gradient hydrogels can introduce continuous transitions in mechanical and biochemical cues, providing a low-modulus and highly hydrated environment on the cartilage side while gradually transitioning toward stiffer or mineralization-prone properties near the subchondral bone region [301, 302]. When combined with three-dimensional bioprinting, these approaches offer more anatomically and mechanically relevant strategies for osteochondral unit regeneration [303, 304].

In OA treatment, the value of hydrogels lies not only in drug loading, but also in their ability to intervene in the joint microenvironment through structural and functional design. Compared with conventional intra-articular injection formulations, injectable hydrogels can undergo in situ gelation within the joint cavity, prolong drug retention, provide mechanical cushioning, and participate in joint microenvironment modulation [305, 306]. Because freely injected drugs are readily diluted by synovial fluid and cleared from the joint cavity, shear-thinning and self-recovering hydrogels have been developed to maintain injectability during administration and rapidly restore stable networks after injection, thereby improving local residence under cyclic motion [307, 308]. Hyaluronic acid-based injectable anti-inflammatory hydrogels, for example, can form local depots and provide sustained release to reduce inflammation-driven cartilage matrix degradation [307].

OA progression is driven by inflammation, oxidative stress, ECM degradation, abnormal chondrocyte phenotype, lubrication failure, and pathological remodeling of the osteochondral interface. Therefore, multi-factor delivery and on-demand release have become important design principles for hydrogel-based OA intervention. Qiu et al. developed an injectable multifunctional microsphere system using sequential release to rapidly alleviate inflammatory pain and subsequently shift the immune microenvironment toward a reparative phenotype, thereby improving both synovial inflammation and cartilage degeneration [309]. ROS-responsive hydrogels further convert oxidative stress into a release trigger, enabling therapeutic agents to act preferentially in diseased joint regions [310, 311]. In addition to biochemical regulation, reconstruction of joint lubrication has gained increasing attention. Han et al. developed GelMA@DMA-MPC hydrogel microspheres that reduced the friction coefficient by forming a stable hydration layer while enabling controlled drug release, demonstrating that the intrinsic lubricating and cushioning properties of hydrogels can contribute directly to OA therapy [312]. Next-generation systems have also expanded from the joint cavity to the osteochondral unit, such as osteochondral interface-targeted glucosamine-based hydrogels for localized teriparatide delivery and hydrogels capable of suppressing aberrant neurovascularization at the osteochondral interface [313, 314]. Furthermore, piezoelectric hydrogels can generate biologically relevant electrical signals under mechanical stimulation, providing a physical-field-assisted strategy for modulating inflammation and cartilage repair [263], while hydrogel-based extracellular vesicle delivery offers a cell-free approach for early OA intervention through immunomodulation and cartilage protection [315].

#### Tendon repair and tendon–bone interface regeneration

Tendons are highly ordered load-bearing tissues composed of parallel collagen fibers and are responsible for transmitting muscle-generated forces to the skeleton. Despite their essential biomechanical function, tendons have limited intrinsic regenerative capacity. Clinically, acute ruptures, such as Achilles tendon or rotator cuff injuries, and chronic overuse-related tendon damage often heal slowly and are characterized by scar tissue formation, disorganized collagen deposition, and incomplete recovery of mechanical performance [316]. After surgical repair, re-rupture and persistent dysfunction remain major concerns due to excessive inflammation, insufficient mechanical strength, and poor collagen alignment in newly formed tissue [317].

The failure of tendon repair is closely associated with mismatched mechanical environments, dysregulated cell fate, fibrotic healing, and insufficient regeneration at the tendon–bone interface. Conventional suturing or rigid patch materials can restore structural continuity in the short term, but they generally do not provide an anisotropic mechanical microenvironment comparable to native tendon tissue, nor do they effectively guide matrix remodeling along the characteristic fiber orientation of tendons [317, 318]. Hydrogels have therefore been introduced as functional adjuncts to surgical repair or as platforms for constructing regenerative microenvironments [318].

A major challenge in tendon repair is balancing the softness of hydrogels with the high tensile requirements of tendon tissue. Native tendons exhibit nonlinear mechanical behavior and strong anisotropy, whereas conventional isotropic hydrogels are often insufficient in tensile strength and fatigue resistance. Recent studies have therefore shifted from simply increasing overall modulus toward structure-guided mechanical reinforcement. Fibrillar architectures, double-network systems, and nanoreinforcing components can provide hydrogels with tendon-like tensile behavior while maintaining high water content and cytocompatibility [319]. Inspired by the aligned collagen architecture of tendons, oriented fibers or traction-induced anisotropic networks can guide cells to align along the principal stress direction under mechanical loading, promoting ordered collagen deposition and improved mechanical function in vivo [320]. These strategies suggest that hydrogels are not intended to replace tendons directly, but rather to provide a mechanically perceptible and directionally instructive environment for tendon regeneration.

Another important challenge is the tendency toward scar-mediated healing. After tendon injury, fibroblasts can shift toward a highly proliferative and contractile phenotype, leading to excessive deposition of disorganized collagen, increased tissue stiffness, restricted gliding function, and compromised mechanical performance [317]. Hydrogel systems can help regulate this process by tuning stiffness, stress relaxation, degradation kinetics, and local biochemical signaling. Through these parameters, hydrogels may attenuate excessive fibrotic responses and promote the transition of resident cells toward tendon progenitor-like or mature tenocyte-like phenotypes [321]. In addition, the sustained release of anti-inflammatory molecules, growth factors, or microRNAs from hydrogels can reduce early inflammatory intensity, improve collagen organization, and support more favorable long-term functional outcomes [322].

Tendon–bone interface regeneration represents a particularly important application scenario for hydrogels. The native enthesis exhibits a continuous gradient from soft tendon to mineralized bone, whereas conventional repair often results in mechanically and biologically discontinuous interfaces, contributing to re-rupture and postoperative pain [318]. Multilayered or gradient hydrogel systems have therefore been developed to spatially regulate mechanical properties, mineralization tendency, and presentation of biological cues, creating a progressive transition from a tendon-like to a bone-like microenvironment [323]. These hydrogels can serve not only as carriers for cells and bioactive factors, but also as instructive matrices that promote in situ formation of interface tissues resembling physiological enthesis architecture, thereby providing a material basis for functional tendon–bone interface regeneration.

Overall, hydrogel-based strategies for musculoskeletal tissue repair are moving from simple filling or delivery systems toward mechanically adaptive, bioactive, and stage-responsive regenerative platforms. In bone defects, hydrogels mainly address mechanical stabilization, osteogenic induction, vascularization, and degradation matching. In cartilage injury and OA, they provide dynamic mechanical microenvironments, ECM-mimetic biochemical cues, local retention, lubrication, and inflammation-responsive therapeutic release. In tendon repair, hydrogels support anisotropic mechanical guidance, fibrotic microenvironment regulation, and gradient reconstruction of the tendon–bone interface. Across these applications, the key translational challenge remains the integration of sufficient mechanical robustness, biological activity, long-term durability, and clinically practical fabrication or delivery methods.

### Hydrogel-mediated local cancer therapy

Cancer treatment remains constrained by several long-standing challenges, including insufficient tumor-site drug accumulation, poor water solubility and narrow therapeutic windows of many anticancer agents, systemic toxicity, drug resistance, and difficulty in fully eliminating residual tumor cells after local intervention. With the continued development of precision medicine and targeted therapy, how to maintain antitumor efficacy while reducing systemic adverse effects has become a central issue in cancer treatment. To address this challenge, polymer-based drug delivery systems have been developed in diverse forms, including nanoparticles, micelles, hydrogels, microspheres, and drug–polymer conjugates. These systems can prolong drug residence time in vivo, enhance drug stability, reduce nonspecific damage to healthy tissues to some extent, and improve drug accumulation at tumor sites [21, 324]. Among these platforms, hydrogels demonstrate distinctive advantages owing to their high water content, favorable biocompatibility, tunable structure and function, and capacity for in situ formation, sustained release, and local microenvironmental regulation [325, 326].

In cancer therapy, the value of hydrogels is not limited to their ability to serve as local drug depots. More importantly, hydrogel networks can be engineered to respond to tumor-associated cues, including acidity, hypoxia, oxidative stress, enzyme activity, and external physical stimulation, thereby enabling localized release of therapeutic agents and coordinated activation of multiple antitumor mechanisms. In addition, hydrogels can be designed to retain photothermal agents, photosensitizers, sonosensitizers, immunomodulators, chemotherapeutics, or radiosensitizers at tumor sites, improving local therapeutic intensity while reducing systemic exposure. Therefore, hydrogel-mediated cancer therapy has gradually evolved from simple local chemotherapy toward integrated therapeutic systems that combine drug delivery, immune microenvironment modulation, physical-field-assisted tumor ablation, postoperative recurrence prevention, and tissue protection. Representative localized therapeutic modalities include photothermal therapy (PTT), photodynamic therapy (PDT), sonodynamic therapy (SDT), and radiotherapy (RT), which induce tumor cell death through local heat generation, ROS production, ultrasound-triggered effects, or radiation-induced damage [326]. The following sections summarize these hydrogel-assisted strategies from the perspective of material design logic and therapeutic function rather than as isolated disease-specific applications (Table 3).

**Table 3:** Representative hydrogel-based strategies for local cancer therapy

Diseases	Biomaterial	Active substances	Advantages	References
Radiation therapy	Sodium alginate	Elesclomol-copper complex, galactose	Sustained local release synergizes with radiotherapy	[327]
	PVA-TSPBA hydrogel	Stimulator of interferon gene agonist ADU-S100 and adeno-associated virus serotype 9-mediated soluble programmed cell death protein-1	Long-lasting release, radio-immuno-sensitizer	[328]
	Agarose-based thermo-sensitive hydrogel	Tetraphenylethylene-benzobisthiadiazole and glutaminase inhibitor CB-839	Molecular motion regulation enhances mild photothermal synergy	[329]
	Smac-TLR7/8 peptide	Toll-like receptor 7/8 agonist	Overcomes radioresistance	[330]
Photodynamic therapy	Polyacrylic acid-chitosan interpenetrating network dry hydrogel	5-aminolevulinic acid	Strong mucosal adhesion improves patient comfort	[331]
	Sodium alginate	Aggregation-induced-emission photosensitizer CQu, Ca^2+^	Prevents relapse, abscopal effect	[332]
	Acrylamide, poly(ethylene glycol) diacrylate, 2-(hydroxyethyl) acrylate	Poly(3-hexylthiophene) semiconducting polymer nanoparticles	Dual-function photoinitiator and photosensitizer, no toxic co-initiator needed	[333]
Photothermal therapy	Low-melting point agarose	Honeycomb-like copper manganese oxide nanozyme, copper peroxide nanoflower, photothermal agent IR820	Multifunctional synergistic therapy, microenvironment-responsive	[334]
	CD47 antibody hydrogel	R848 nanoparticles, IR820	Prevents recurrence and metastasis	[335]
	Agarose	Aggregation-induced emission photothermal agent, thioridazine	Photothermal-triggered drug release, targets cancer stem cells	[336]
	Silk fibroin/perfluorocarbon nanoemulsion	Doxorubicin, oxygen	Prevents recurrence, promotes healing	[337]
Sonodynamic therapy	Gelatin methacryloyl (GelMA)	Hematoporphyrin (HP), Erlotinib (Er), Perfluorooctyl bromide (PFOB)	Intelligent targeting, hypoxia relief, synergistic therapy	[338]
	Chitosan/β-glycerophosphate thermosensitive hydrogel	Curcumin	Clears residue, trackable	[339]
	Hyaluronic acid (HA), Pluronic F-127 (F127)	Ti-MOF-Au, PEG-TK-DOX, pirfenidone (PFD)	Deep penetration, sonodynamic therapy, immune activation, anti-metastasis	[340]
	NapFFSGP self-assembling peptide	Ironporphyrin	Anti-metastasis, immune boost	[341]

#### Light-responsive hydrogel strategies: photothermal and photodynamic therapy

Light-responsive hydrogel systems represent an important class of localized cancer treatment platforms because they can couple spatially controlled irradiation with tumor-site retention of therapeutic agents. PTT converts light energy into heat through photothermal nanomaterials, such as gold nanoparticles, carbon-based nanomaterials, and transition metal sulfides, leading to local temperature elevation and thermal damage to tumor cells [342, 343]. PDT, by contrast, relies on photosensitizers, light irradiation, and molecular oxygen to generate ROS, such as singlet oxygen, which can oxidize intracellular lipids, proteins, and DNA and induce apoptosis or necrosis [344]. Although both strategies offer spatial selectivity and relatively limited systemic exposure, their efficacy is often restricted by insufficient local accumulation of therapeutic agents, heterogeneous intratumoral distribution, limited light penetration, and hypoxia in solid tumors [345–347]. Hydrogel systems provide a materials-based solution to these limitations by enhancing local retention of phototherapeutic agents, improving their stability, enabling controlled release, and modulating the tumor microenvironment.

For PTT, temperature-responsive hydrogels are particularly attractive because the heat generated under near-infrared irradiation can simultaneously mediate tumor ablation and trigger drug release. During light-induced heating, hydrogel networks may undergo phase transition, swelling/deswelling behavior, or structural rearrangement, thereby facilitating synchronized release of chemotherapeutic or immunomodulatory agents and producing synergistic antitumor effects [348, 349]. This strategy has been widely explored in tumor models such as breast cancer, melanoma, and osteosarcoma. In breast cancer, where surgery remains a primary therapeutic option, residual tumor cells after resection are a major source of recurrence and metastasis, while postoperative adjuvant therapies are often limited by toxicity and drug resistance [350, 351]. Hu et al. developed an injectable thermosensitive CuMnOx@CuO_2_@IR820 hydrogel integrating CuMnOx nanozymes, CuO_2_ nanoflowers, and the photosensitizer IR820. Under near-infrared irradiation, this system combined low-temperature PTT with PDT-like ROS generation to ablate tumor cells and bacteria, while oxygen generation and copper ion release contributed to postoperative wound healing and angiogenesis. In a breast cancer model, the hydrogel delayed tumor recurrence and reduced recurrent tumor volume, indicating the value of hydrogel-based phototherapy for postoperative tumor control and wound management [334].

Beyond direct tumor ablation, hydrogel-assisted PTT can be used to remodel the tumor immune microenvironment. Sheng et al. constructed a hydrogel system combining IR820-conjugated apoptotic body-based nanoparticles loaded with R848 and a hydrogel depot containing anti-CD47 antibodies. The apoptotic body-based nanoparticles enhanced tumor targeting and penetration through a macrophage-mediated delivery mechanism, whereas the hydrogel matrix enabled sustained local release. Upon near-infrared irradiation, this platform induced photothermal tumor cell death and immunogenic cell death, activated antigen-presenting cells, promoted tumor-associated macrophage repolarization from an immunosuppressive M2 phenotype toward an antitumor M1 phenotype, and blocked the CD47–SIRPα axis to enhance macrophage phagocytosis. In a 4T1 breast tumor model, the combination treatment produced stronger tumor suppression than single-function controls, demonstrating how hydrogels can integrate photothermal therapy with local immunomodulation [335]. In another example, Zhang et al. designed an intelligent temperature-responsive nanohydrogel system co-loading an aggregation-induced emission photothermal agent and thioridazine. Mild photothermal heating triggered gel–sol transition and promoted intratumoral penetration of the released agents, while thioridazine suppressed dopamine receptor-related signaling and helped eliminate cancer stem cell subpopulations, thereby reducing recurrence and metastasis in a 4T1 breast cancer model [336].

PDT-assisted hydrogel systems follow a related design principle, but their core therapeutic output is ROS-mediated cytotoxicity rather than heat. In oral potentially malignant disorders and early oral cancer, local delivery is particularly important because conventional treatment may impair oral functions such as mastication, speech, swallowing, and taste, while conventional PDT can be associated with photosensitivity and other adverse effects [352–354]. Wang et al. developed a dry-state poly(acrylic acid)–chitosan–5-aminolevulinic acid interpenetrating network hydrogel patch with strong adhesion to moist oral mucosa. This patch provided stable local ALA delivery for PDT, suppressed lesion progression in a premalignant hamster cheek pouch model, and improved patient convenience and satisfaction in a clinical study involving OPMD volunteers [331]. This example illustrates how hydrogel design can address not only therapeutic efficacy, but also local retention, patient compliance, and functional preservation.

For deeper or more immunosuppressive tumors, PDT hydrogels have increasingly been combined with additional therapies to amplify antitumor immunity. In colorectal cancer, therapeutic outcomes are often influenced by an immunosuppressive tumor microenvironment that limits antitumor immune responses and contributes to recurrence and metastasis [355, 356]. Lyu et al. developed a PDT-enhanced alginate hydrogel platform integrating flower-like calcium carbonate nanoparticles and an aggregation-induced emission luminogen. Under acidic tumor conditions, released Ca^2+^ induced in situ alginate gelation, forming a stable three-dimensional network that retained the photosensitizer at the tumor site. When combined with PDT and FLASH radiotherapy, this hydrogel system enhanced immunogenic cell death, increased CD8^+^ T-cell infiltration, and significantly suppressed tumor growth in a CT26 colon cancer model [332]. In glioblastoma, where residual tumor cells after surgery are difficult to eradicate and resistance to conventional therapies remains a major challenge [357], Abalos et al. developed a light-responsive hydrogel system containing poly(3-hexylthiophene) semiconductor polymer nanoparticles. These nanoparticles functioned as visible-light photoinitiators to generate smart hydrogels with defined architectures and, upon light irradiation, induced local ROS production that reduced glioma cell viability while also exhibiting antibacterial activity against S. aureus [333]. Together, these studies demonstrate that hydrogel-assisted PDT can extend from simple photosensitizer retention to integrated platforms for tissue-specific delivery, immune activation, and postoperative local control.

#### Hydrogel-assisted deep-tissue and radiation-based therapies: sonodynamic therapy and radiotherapy

Although light-responsive therapies offer strong spatial control, their efficacy can be restricted in deep tumors by limited tissue penetration. SDT has therefore attracted increasing interest because low-intensity ultrasound can penetrate deeper tissues and activate sonosensitizers to generate ROS, thereby inducing tumor cell death [358, 359]. Compared with PDT, SDT offers advantages in penetration depth and spatial localization, although its therapeutic efficiency, long-term safety, and in vivo mechanisms still require further validation [359, 360]. A major limitation of SDT is the heterogeneous distribution and rapid clearance of sonosensitizers, which reduces local ROS generation. Hydrogels can address this issue by retaining sonosensitizers at tumor sites, coordinating their release with ultrasound stimulation, and incorporating additional components that regulate hypoxia, drug resistance, or immune suppression.

In non-small cell lung cancer, acquired resistance to EGFR tyrosine kinase inhibitors is associated with hypoxia, abnormal downstream signaling, and insufficient drug delivery [361, 362]. Peixia Zhang and colleagues developed a self-assembled multifunctional nanocomposite based on erlotinib-modified chitosan for combined SDT and targeted molecular therapy. This system co-delivered hematoporphyrin as a sonosensitizer and perfluorooctyl bromide as an oxygen carrier, thereby improving local oxygenation, enhancing drug penetration, and promoting ultrasound-triggered ROS generation. Mechanistically, the combined treatment downregulated EGFR, phosphorylated EGFR, and HIF-1α, suggesting that hydrogel-like self-assembled platforms can be used to coordinate microenvironment modulation with targeted therapy in refractory lung cancer [338].

For renal cell carcinoma, local recurrence after incomplete thermal ablation remains a clinically relevant problem, especially in patients who are not suitable candidates for surgery [363]. Cuixian Li and colleagues developed a thermosensitive hydrogel encapsulating curcumin-loaded hybrid mesoporous organosilica nanoparticles containing tetrasulfide bonds. This system exhibited in situ gelation and prolonged tumor-site retention, while ultrasound stimulation triggered curcumin-mediated ROS generation. The tetrasulfide-containing nanocarrier further responded to elevated intratumoral glutathione, enhancing nanocarrier degradation and drug release. In a renal cell carcinoma xenograft model, Cur@HMON@gel combined with ultrasound suppressed residual tumor growth and induced tumor necrosis, indicating the utility of hydrogel-assisted SDT for residual tumor management [339].

In highly invasive and metastatic tumors, hydrogel-assisted SDT can also be integrated with chemotherapy, ECM remodeling, ferroptosis, and immunotherapy. Zideng Dai and colleagues developed an ultrasound-mediated hydrogel platform co-encapsulating Ti-MOF-Au sonosensitizers, PEG-TK-DOX prodrug, and pirfenidone for aggressive malignancies such as pancreatic cancer and triple-negative breast cancer. This platform enabled ultrasound- and H_2_O_2_-responsive drug release, improved deep-tissue penetration, remodeled the tumor immune microenvironment, and suppressed tumor growth and pulmonary metastasis [340]. From the perspective of intrinsic material functionality, Hongxia Zhang and colleagues designed self-assembling peptide hydrogels with inherent anti-metastatic activity and further incorporated iron porphyrin to construct a minimalist platform combining SDT and ferroptosis. Upon ultrasound irradiation, the system generated ROS and amplified lipid peroxidation, inducing apoptosis, ferroptosis, and immunogenic cell death, while enhancing antitumor immune responses in a 4T1 breast cancer model [341]. These studies highlight the capacity of supramolecular and injectable hydrogels to transform ultrasound stimulation into localized, multimodal antitumor responses.

Radiotherapy is another major local cancer treatment modality that uses high-energy radiation, including X-rays, γ-rays, or proton beams, to damage tumor cell DNA and inhibit tumor growth [364]. Although RT provides spatially defined tumor control, collateral injury to surrounding normal tissues can lead to fibrosis, chronic inflammation, vascular damage, radiation-induced skin injury, and refractory wounds, thereby limiting dose escalation and therapeutic efficacy [365]. Hydrogels have been explored in RT-related applications from two complementary perspectives: enhancing local radiosensitivity at tumor sites and protecting or repairing normal tissues damaged by radiation.

For radiosensitization, hydrogel systems can locally retain metabolic modulators, immune agonists, or radiosensitizing agents, reducing systemic exposure while enhancing tumor response to irradiation. Shen et al. developed an injectable hydrogel incorporating Cu^2+^, Elesclomol, and galactose to achieve sustained release and metabolically enhanced cuproptosis. This platform increased tumor susceptibility to radiotherapy-induced damage and reduced PD-L1 expression, thereby improving both radiosensitivity and responsiveness to immune checkpoint blockade. In a CT26 colorectal cancer model, the hydrogel combined with radiotherapy significantly suppressed tumor growth and prolonged survival [327]. In glioblastoma, Sun et al. designed a bioresponsive in situ-forming hydrogel scaffold integrating a STING agonist and an AAV-mediated local PD-1 secretion system. During radiotherapy, elevated ROS triggered drug release from the hydrogel at the tumor resection site, activating innate immune responses, enhancing dendritic cell maturation and CD8^+^ T-cell infiltration, and reducing immunosuppressive signaling in a GBM resection model [328].

For radiation-induced tissue injury, hydrogels can provide antioxidant, antibacterial, adhesive, and repair-promoting functions. Radiation-associated skin damage is driven largely by sustained oxidative stress, DNA damage, apoptosis, impaired barrier function, secondary infection, and persistent inflammation [366]. Zhang et al. developed a bioactive PolyLA hydrogel composed of α-lipoic acid, arginine, and silk fibroin, integrating antioxidant, antibacterial, and tissue-repair functions. In models of radiation-induced skin injury, this hydrogel reduced inflammation, promoted hair follicle regeneration, improved histological reconstruction, and regulated collagen deposition; it also accelerated closure in MRSA-infected full-thickness skin wounds [367]. The same research group further proposed a dual-system strategy for postoperative breast cancer care, combining an implantable silk fibroin/perfluorocarbon hydrogel loaded with doxorubicin for tumor-site oxygen delivery and radiosensitization with a topical antioxidant bioadhesive patch for skin protection. This system improved tumor control while reducing radiation-induced skin injury, supporting the concept that hydrogels can simultaneously enhance antitumor therapy and protect normal tissues during radiotherapy [337].

Overall, hydrogel-mediated local cancer therapy has evolved from simple drug retention toward integrated therapeutic platforms that combine controlled release, tumor microenvironment regulation, physical-field responsiveness, immune activation, and tissue protection. In light-responsive strategies, hydrogels improve the local stability and retention of photothermal agents or photosensitizers, while enabling heat- or ROS-triggered drug release and immune modulation. In SDT, hydrogels prolong sonosensitizer retention, improve oxygenation or penetration, and support multimodal mechanisms such as chemotherapy, ferroptosis, and immunogenic cell death. In RT-related applications, hydrogels can function as local radiosensitizing depots, immune-modulating scaffolds, or protective dressings for radiation-induced tissue injury. Across these modalities, the central advantage of hydrogels lies in their ability to spatially confine potent therapeutic effects while coordinating multiple biological processes within the tumor or postoperative microenvironment.

### Neurological disease modeling and neural repair

Neurological disorders, including central nervous system (CNS) injuries, peripheral nerve injuries, and chronic neurodegenerative diseases, remain difficult to treat because neural tissues possess highly complex anatomical organization and limited intrinsic regenerative capacity. Physical trauma, ischemic injury, and degenerative pathological processes can disrupt neuronal cell bodies, axons, myelin structures, and supporting glial networks, often leading to long-term sensory, motor, or cognitive dysfunction. These outcomes are further aggravated by sustained inflammation, impaired vascular support, oxidative stress, glial scar formation, and growth-inhibitory signals, all of which restrict axonal regeneration and neural circuit reconstruction [337, 368]. Therefore, effective neurological repair requires not only neuroprotection, but also coordinated regulation of inflammation, scar formation, axonal guidance, myelin repair, vascular support, and functional circuit reconstruction.

In this context, hydrogel-based strategies are increasingly being explored as local platforms for neural protection, therapeutic delivery, microenvironment regulation, and disease modeling [369]. Rather than serving only as structural fillers, these systems are designed to address specific neurological challenges, including neuronal survival, remyelination, axonal extension, glial scar modulation, and long-term integration with host neural tissue. The following sections summarize representative hydrogel-based approaches for neurodegenerative disease-related diagnosis and intervention, as well as CNS and peripheral nerve injury repair (Table 4).

**Table 4:** Representative hydrogel-based strategies for neurological disease modeling and neural repair

Diseases	Biomaterial	Active substances	Advantages	References
Neuropathic diseases	Gelatin methacrylamide microbeads; Cystamine-modified gold nanoyarn balls; Conductive Microporous Hydrogel	/	Microporous structure enhances cell infiltration, conductivity promotes neural repair	[370]
	Agarose/Gelatin/Polypyrrole	/	Conductive, injectable, scar-suppressing	[371]
	Thermo-sensitive polymer electroactive hydrogel based on poly(ethylene glycol)-co-polyvaline grafted with tetraniline, mPEG-PLV-TA	Nerve Growth Factor	Electroactive hydrogel synergizes with electrical stimulation to promote endogenous neurogenesis	[372]
	Hyaluronic acid-graft-dopamine/designer peptide HGF-(RADA)4-DGDRGDS	Neurotrophin-3, curcumin	Remodels scar to guide axons	[373]
	Hyaluronic Acid-Pamidronate-Magnesium nanocomposite hydrogel, HA-Pam-Mg hydrogel	Mg^2+^	Sustained magnesium ion release promotes nerve regeneration, combined with 3D-printed conduit	[374]
	Bisphosphonated-alginate/silk fibroin interpenetrating network hydrogel	Mg^2+^	Cell-adaptive boosts regeneration	[375]
Neurodegenerative diseases	polyethylene (glycol) diacrylate	Catalytic hairpin assembly probes	High sensitivity, enzyme-free amplification, portable detection	[376]
	Polyacrylamide-based microgel containing polydopamine and imidazole groups	Superoxide dismutase, copper ions	Antioxidant, anti-inflammatory, pro-neurogenic	[377]
	poly(OEGMA-co-AEMA)-g-caffeic acid; Dialdehyde Poloxamer 407	Fibroblast growth factor 21; Edaravon; Caffeic acid	Asynchronous release, multi-mechanism, injectable self-healing	[378]
	Carboxymethyl chitosan/tannic acid-modified dialdehyde polyurethane nano-crosslinker	/	Targets RIPK1 to suppress inflammation	[379]

#### Neurodegenerative diseases and functional diagnostic or therapeutic platforms

Neurodegenerative diseases can be broadly categorized according to the predominant clinical functions affected, including cognitive impairment-dominant disorders, motor dysfunction-dominant disorders, and mixed forms that involve both cognitive and motor decline. Despite differences in etiology and pathology, these diseases share a fundamental limitation: once neural circuits are progressively damaged, the CNS has limited capacity to restore signal transmission and functional connectivity [380, 381]. Therefore, hydrogel-based strategies in this field mainly focus on two directions. One is to construct diagnostic platforms that improve the detection of disease-related biomarkers, especially for early-stage or dynamically progressing conditions. The other is to deliver neuroprotective or regenerative agents and regulate oxidative stress, neuroinflammation, and neuronal survival in local pathological environments.

Alzheimer’s disease (AD) is a representative cognitive impairment-dominant neurodegenerative disorder characterized by progressive memory loss and cognitive decline. Current diagnostic approaches often rely on positron emission tomography imaging or the detection of amyloid-β and amyloid precursor protein in cerebrospinal fluid, but these methods are limited by invasiveness, procedural complexity, and high cost [382]. Circulating blood biomarkers, particularly microRNAs transported by exosomes across the blood–brain barrier, have therefore attracted attention as potential indicators for early AD diagnosis [383, 384]. Based on this concept, Jaewoo Lim and colleagues developed a hydrogel-based miRNA detection platform for sensitive analysis of AD-related miRNAs. In this system, lipoplex nanoparticles were used to load catalyzed hairpin assembly probes and were physically anchored within a hydrogel network, thereby reducing nonspecific hybridization and improving detection specificity. Upon sample introduction, target miRNAs triggered the catalyzed hairpin assembly reaction and generated amplified fluorescence signals. The platform was validated in hippocampal tissue and plasma samples from AD model mice, as well as plasma samples from AD patients, demonstrating the potential of hydrogel-assisted bioanalysis for blood-based AD diagnosis [376]. This example also illustrates that hydrogels in neurodegenerative diseases are not limited to therapeutic delivery, but can also function as diagnostic microenvironments that stabilize molecular probes and amplify disease-relevant signals.

Parkinson’s disease (PD), a representative motor dysfunction-dominant neurodegenerative disease, is characterized by selective loss of dopaminergic neurons in the nigrostriatal pathway and clinical manifestations such as tremor, bradykinesia, rigidity, and postural instability. Its progression is closely associated with oxidative stress, neuroinflammation, mitochondrial dysfunction, and pathological protein accumulation. Although pharmacological therapy and deep brain stimulation can alleviate symptoms to a certain extent, they have limited ability to reverse neuronal degeneration and may involve long-term safety or invasiveness concerns [385–387]. Hydrogels and microgels with antioxidative, anti-inflammatory, and drug delivery functions have therefore been developed as local or brain-targeted intervention systems.

One representative strategy is to construct catalytic microgel systems capable of scavenging ROS and modulating neuroinflammation. Lin Jiang and colleagues designed a superoxide dismutase/copper ion-integrated microgel system with both SOD-like and catalase-like activities. Through acetylcholine modification, the microgels achieved enhanced brain targeting and blood–brain barrier accumulation, while PDA and imidazole coordination stabilized copper ions to support cascade catalytic ROS degradation. The ester-bond-crosslinked structure further endowed the microgels with controllable degradability, which supported dopamine release and neural regeneration. In PD mouse models, intracerebral administration of mCu-PDA/SOD microgels improved behavioral outcomes, attenuated neuroinflammation, and reduced pathological protein accumulation through regulation of the CX3CL1/CX3CR1–NF-κB–NLRP3 axis [377]. From the perspective of multistage and multidrug intervention, Junpeng Xu and colleagues developed an antioxidant self-healing hydrogel with micro–nano structural features capable of co-loading FGF21 and edaravone. This triple drug delivery system enabled asynchronous release according to PD pathological progression, providing early neuroprotection while sustaining ROS clearance, neuroinflammation modulation, and neural repair during later stages [378]. In addition, Xu and colleagues reported an antioxidant injectable chitosan hydrogel crosslinked by tannic acid-modified dialdehyde polyurethane nanoparticles, with receptor-interacting protein kinase 1 identified as a key target. This system scavenged ROS, promoted neural stem cell proliferation, induced macrophage polarization toward an anti-inflammatory phenotype, and improved motor function in a 6-OHDA-induced PD rat model [379]. Collectively, these studies show that hydrogel-based platforms can integrate redox regulation, immune modulation, drug release, and neural stem cell support for neurodegenerative disease intervention.

#### CNS and peripheral nerve injury repair

Neural injuries are generally divided into CNS injuries and peripheral nervous system injuries, and may result from traffic or sports accidents, war-related trauma, violent injuries, tumor resection, iatrogenic damage, or stroke. These injuries often lead to structural disruption of neural tissues and long-term sensory, motor, autonomic, or cognitive dysfunction, imposing substantial physical, psychological, and socioeconomic burdens on patients and their families [388]. Conventional pharmacological treatments and surgical interventions mainly aim to reduce secondary injury, stabilize the lesion, or relieve symptoms, but they remain limited in promoting neural regeneration, reconstructing neural circuits, and restoring neurological function [389]. In this context, hydrogels are increasingly explored as injectable fillers, cell or factor delivery platforms, conductive scaffolds, adhesive matrices, and nerve guidance materials.

CNS injuries are typically initiated by primary mechanical damage, followed by secondary pathological cascades involving inflammation, oxidative stress, excitotoxicity, edema, neuronal apoptosis, and tissue destruction [390]. In addition, glial scar formation and growth-inhibitory molecules at the lesion site restrict axonal regeneration and neural circuit reconstruction, making CNS injury a major cause of long-term disability [391]. Traumatic brain injury (TBI) often results in cavitary tissue defects after local brain necrosis, leaving insufficient structural support for cell migration and tissue regeneration. Meanwhile, microglia, macrophages, and activated astrocytes sustain inflammation, promote glial scar formation, and suppress angiogenesis and neurogenesis [392]. To address these bottlenecks, Ru-Siou Hsu and colleagues developed a conductive microporous hydrogel that integrates structural guidance with remote electrical stimulation. The system consists of electromagnetized gold yarn balls and negatively charged injectable microbeads, which self-assemble into a conductive porous scaffold. After injection, the hydrogel provides an interconnected architecture for cell infiltration, and under an external high-frequency magnetic field, the gold yarn balls generate localized electrical stimulation that enhances brain-derived neurotrophic factor expression and calcium ion permeability. In a mouse TBI model, this system promoted axonal infiltration and reduced lesion cavity volume, indicating the potential of conductive injectable hydrogels for post-traumatic neural repair [370].

Spinal cord injury (SCI) is another severe form of CNS injury, often resulting in irreversible impairment of motor, sensory, and autonomic functions [393]. After SCI, acute mechanical trauma initiates edema, hypoxia–ischemia, inflammation, apoptosis, and necrosis, producing a cytotoxic microenvironment and irregular cystic cavities that further impede spontaneous repair [394]. Injectable hydrogels can conformally fill these cavities, provide structural support, and create a more favorable microenvironment for neural regeneration. Biao Yang and colleagues developed an injectable composite hydrogel composed of polypyrrole, gelatin, and agarose. Polypyrrole introduced electrical conductivity, gelatin supported cell adhesion and differentiation, and agarose improved mechanical strength and thermal stability. In a rat spinal cord hemisection model, this hydrogel reduced lesion cavity size and promoted tissue healing [371]. To further incorporate electrical regulation, Wei Liu and colleagues constructed a thermosensitive electroactive hydrogel based on a polyaniline-related peptide polymer system capable of loading nerve growth factor. When combined with exogenous electrical stimulation, this hydrogel promoted neuronal differentiation, inhibited astrocytic differentiation, enhanced endogenous neural regeneration, and improved motor recovery in SCI rats [372]. In another strategy, Zan Tan and co-workers designed an adhesive biomimetic hydrogel consisting of hyaluronic acid-dopamine and a functional peptide. This material matched the mechanical properties of spinal cord tissue, adhered strongly to lesions, promoted parallel infiltration of PDGFRβ^+^ cells, remodeled dense scar tissue into an aligned fibrous matrix, and, when loaded with NT3 and curcumin, enhanced axonal regeneration and functional recovery in a canine SCI model [373].

Peripheral nerve injury (PNI) involves disruption of nerves outside the brain and spinal cord, impairing motor, sensory, and autonomic functions. Clinical repair relies mainly on nerve grafting and nerve guidance conduits, with autologous nerve grafting regarded as the gold standard for long-segment defects. However, donor nerve availability and donor-site complications, including sensory loss and neuropathic pain, limit its broad application [395, 396]. Hydrogel-based strategies for PNI are therefore mainly designed to provide biochemical cues, mechanical adaptability, topographical guidance, and electrical coupling within nerve conduits or at neural interfaces.

Bioactive ion delivery represents one such approach. Mg^2+^ can promote axonal growth and functional recovery, but its rapid diffusion and poorly controlled release limit therapeutic efficacy. Zhi Yao and colleagues developed a bisphosphonate-modified injectable hydrogel for sustained Mg^2+^ release. When incorporated into a PCL nerve conduit in a rat sciatic nerve defect model, this system enhanced axonal regeneration, remyelination, reinnervation, and functional recovery through mechanisms involving PI3K/Akt signaling and Sema5b upregulation [374]. Building on the need to recapitulate the dynamic biochemical and mechanical microenvironment of nerve regeneration, Yisheng Gao and co-workers developed an adaptive hydrogel nerve conduit based on dynamic coordination between bisphosphonate-modified alginate and Mg^2+^, combined with a silk fibroin interpenetrating network. This system balanced structural stability with mechanical plasticity and promoted Schwann cell migration, axonal extension, target muscle protection, and functional recovery in a rat sciatic nerve defect model [375].

Topographical guidance is another key requirement for long-segment PNI repair. Conventional hollow nerve guidance conduits often fail to provide sufficient structural and biochemical support for axonal regeneration beyond critical defect lengths. To address this limitation, Shuhui Yang and colleagues constructed an interpenetrating nanofibrous hydrogel composed of aligned fibrinogen and functionalized self-assembling peptides by combining electrospinning and molecular self-assembly. The aligned fibrous architecture provided spatial guidance for axonal extension, while peptide-based biochemical cues supported Schwann cell behavior. In a 15 mm sciatic nerve defect model, this hydrogel bridged the nerve gap, promoted remyelination and muscle reconstruction, and achieved motor recovery comparable to autologous nerve grafting [397].

Because neural tissues are sensitive to electrical signals, conductive hydrogel systems have also become important for PNI repair and neural interface engineering. Hongyun Xuan and colleagues designed an injectable, self-healing, conductive hyaluronic acid-based hydrogel containing degradable disulfide crosslinks and in situ polymerized pyrrole structures. This hydrogel combined mechanical robustness, self-healing behavior, degradability, and electrical conductivity. It effectively transmitted electrical signals in an in vitro sciatic nerve–gastrocnemius muscle model and supported peripheral nerve regeneration and functional recovery in subsequent experiments [398]. Conductive hydrogels have also been extended from regenerative scaffolds to neural interface materials. Ming Yang and colleagues integrated a conductive bioadhesive hydrogel with cuff electrodes to improve electrical coupling with small peripheral nerves. The hydrogel filled microscopic gaps between the electrode and nerve tissue, provided injectability, conductivity, adhesion, and anti-swelling properties, and enabled stable electrical stimulation during chronic neuromodulation in a rat vagus nerve model after myocardial infarction [399]. This work illustrates how hydrogel materials can bridge biological tissues and bioelectronic devices, expanding their role from nerve repair to long-term neural regulation.

Overall, hydrogel-based strategies for neurological diseases have shifted from simple scaffold or delivery concepts toward multifunctional platforms that integrate ECM mimicry, mechanical matching, controlled release, electrical modulation, immune regulation, and tissue-specific structural guidance. In neurodegenerative diseases, hydrogels support biomarker detection, antioxidative therapy, neuroinflammation modulation, and staged drug delivery. In CNS injury, injectable, conductive, and adhesive hydrogels can fill lesion cavities, regulate hostile microenvironments, guide axonal regeneration, and support circuit reconstruction. In PNI, hydrogels provide ion delivery, topographical guidance, adaptive mechanics, and electrical coupling within nerve conduits or neural interfaces. Despite these advances, clinical translation remains challenged by the complexity of neural anatomy, the need for long-term functional integration, safety concerns related to intracranial or intrathecal delivery, and the difficulty of matching material degradation with the slow pace of neural repair.

### Cardiovascular repair, interfaces, and monitoring

Cardiovascular diseases (CVDs) remain a major clinical challenge because of their high incidence, high disability rates, and high mortality. Their onset and progression are commonly accompanied by extensive cardiomyocyte loss, endothelial dysfunction, persistent inflammatory responses, vascular injury, and long-term imbalance of the mechanical environments of the heart and vasculature [400, 401]. In clinical practice, the treatment and long-term management of CVDs are limited by the extremely low regenerative capacity of adult cardiomyocytes, impaired re-endothelialization after vascular injury, highly dynamic and stress-concentrated mechanical conditions within diseased cardiovascular tissues, and disease progression that depends strongly on long-term physiological and biochemical signaling changes [402–404]. These features make single-time-point or single-pathway interventions insufficient for addressing the complex and evolving pathological processes of cardiovascular diseases.

Hydrogels have increasingly been explored as multifunctional cardiovascular materials because they can combine tissue-like softness, high water content, tunable mechanical behavior, bioactive delivery capacity, and interfacial compatibility with dynamic tissues. After myocardial injury, hydrogels can provide localized mechanical support and ECM-mimetic cues to attenuate adverse ventricular remodeling; in vascular intervention, hydrogel coatings can regulate stent–vessel interface responses, promote re-endothelialization, and reduce the risk of thrombosis; and in flexible bioelectronics, conductive or adhesive hydrogels can conformally integrate with myocardial tissue to support long-term physiological signal acquisition and prognostic monitoring [165, 405, 406]. Therefore, hydrogel-based cardiovascular strategies are gradually moving beyond passive support or coating functions toward integrated systems that combine repair, interface regulation, sensing, and disease monitoring (Table 5).

**Table 5:** Representative hydrogel-based strategies for cardiovascular repair, interfaces, and monitoring

Diseases	Biomaterial	Active substances	Advantages	References
Myocardial infarction	Polyacrylamide	Curcumin	Synchronous repair and monitoring, high sensitivity, anti-inflammatory and angiogenic	[407]
	Hyaluronic acid methacrylate, poly(ethylene glycol) diacrylate	Containing cardiac troponin I, myoglobin, creatine kinase MB aptamers and their complementary DNA	Fast non-invasive early diagnosis	[408]
	Dopamine-functionalized Alginate, Thioketal-modified Polyethylene Glycol Diacrylate, Gelatin Methacrylate,	ROS-sensitive Thioketal Linkages	Injectable, Asymmetric Adhesion, Conductive, ROS Scavenging	[409]
Cardiovascular prognosis	Gelatin, dextran aldehyde	/	Paintable, high conductivity, good tissue adhesion	[410]
	Oxidized xanthan gum–gelatin–polypyrrole-grafted gelatin	/	Conductive-self-healing-antiarrhythmic	[411]

#### Myocardial repair and vascular interface engineering

Myocardial injury, particularly ischemic damage following myocardial infarction, is a central pathological basis for the development of heart failure. Its long-term outcomes are largely determined by irreversible cardiomyocyte loss, dysregulated inflammation, abnormal ECM remodeling, impaired vascularization, and progressive deterioration of ventricular wall mechanical integrity [402, 403]. Because adult cardiomyocytes have very limited regenerative capacity, the injured myocardium is typically replaced by fibrotic scar tissue. Although this scar tissue preserves structural continuity in the short term, it compromises contractile function and promotes progressive ventricular remodeling through redistribution of wall stress, eventually contributing to heart failure [404]. Current pharmacological and interventional therapies mainly restore perfusion, regulate neurohumoral pathways, or alleviate symptoms, but they are generally insufficient to reverse the transition from structural damage to functional decline at the tissue level.

For myocardial repair, hydrogel design must first address the highly dynamic mechanical microenvironment of the beating heart. Myocardial tissue undergoes rapid contraction and relaxation during each cardiac cycle, exhibits pronounced viscoelasticity and anisotropy, and relies on coordinated electromechanical coupling to maintain synchronous beating. Materials that are excessively stiff may restrict myocardial deformation and increase local stress concentration, whereas materials that are too soft or degrade too rapidly may fail to provide sufficient support; both situations may interfere with cardiac function and potentially increase arrhythmogenic risk [412, 413]. Recent hydrogel strategies have therefore emphasized mechanical matching, stress buffering, and reversible deformation. By regulating polymer chain flexibility, crosslinking density, and dynamic reversible bonds, hydrogels with myocardial-like moduli and stress relaxation behavior can provide temporary mechanical support within infarcted regions, attenuate ventricular wall thinning and dilation, and create a more favorable environment for functional recovery [5, 414].

In addition to mechanical support, local biochemical regulation is essential for myocardial repair because persistent inflammation and fibrotic remodeling strongly influence long-term outcomes. After myocardial infarction, inflammation contributes to clearance of necrotic tissue, but excessive or prolonged activation of fibroblasts and immune cells can accelerate scar formation and suppress functional regeneration [415]. Hydrogels can serve as localized and controllable delivery platforms for anti-inflammatory agents, antifibrotic factors, regulatory nucleic acids, or bioactive proteins, thereby prolonging their retention within injured myocardium and spatially confining their effects [416, 417]. Moreover, hydrogels incorporating inflammation- or ROS-responsive degradable linkages can dynamically adjust their degradation and release profiles according to the lesion microenvironment, allowing stage-specific regulation during different phases of myocardial repair. This lesion-responsive design provides a materials basis for improving the precision of myocardial regeneration therapies.

Hydrogels have also been widely used for the delivery of cells and cell-derived products. Direct intramyocardial injection of therapeutic cells is often limited by low retention, with most transplanted cells cleared within hours to days. Encapsulating cardiac stem or progenitor cells, mesenchymal stem cells, or induced pluripotent stem cell-derived cardiomyocyte-like cells within hydrogels can provide a three-dimensional supportive matrix and mechanical protection, thereby enhancing cell survival and paracrine activity [405, 418]. In addition, the tunable mechanical and biochemical properties of hydrogels can regulate cell fate and secretory profiles, supporting more sustained local release of pro-angiogenic, anti-apoptotic, and immunomodulatory factors. With the growing understanding that extracellular vesicles and exosomes mediate many benefits of cell therapies, hydrogel-based delivery systems have also been applied to cell-free cardiac repair strategies, which may provide cardioprotective effects while reducing some safety concerns associated with living cell transplantation.

Reconstruction of the vascular network is another key determinant of successful myocardial repair. In ischemic myocardium, even if cardiomyocytes or substitute cells survive initially, their long-term function is difficult to maintain without sufficient perfusion. Hydrogel systems are therefore frequently integrated with pro-angiogenic strategies, such as controlled release of VEGF or fibroblast growth factor, or modulation of endothelial cell behavior, to promote neovascular formation and maturation. By tuning pore size, interconnectivity, and degradation rate, hydrogels can provide space for vascular ingrowth while matching the temporal process of vascular maturation, thereby improving myocardial perfusion and supporting sustained functional recovery [414, 419].

Beyond myocardial repair, hydrogel-based materials also play an important role in vascular interface engineering, particularly in cardiovascular stent coatings. Cardiovascular stents are widely used for the treatment of coronary artery stenosis and occlusive vascular diseases, and their long-term performance depends not only on immediate blood flow restoration but also on vascular wall healing after implantation. Although bare-metal stents and drug-eluting stents have reduced acute restenosis, delayed endothelialization, persistent inflammation, thrombosis, and late restenosis remain major barriers to long-term safety and efficacy [415, 420]. These complications arise from unfavorable interactions among the stent surface, endothelial cells, circulating blood components, and immune responses.

Hydrogel coatings provide a promising strategy for constructing more tissue-compatible and functionally instructive stent interfaces. The vascular environment is continuously exposed to pulsatile blood flow and high shear stress; therefore, coatings must maintain structural stability while avoiding thrombosis caused by material delamination or surface roughness. Through covalent anchoring, interfacial polymerization, or dopamine-inspired bioadhesive strategies, hydrogels can be firmly immobilized on metallic or alloy stent surfaces and maintain structural integrity under blood flow, thereby reducing physical disturbance at the blood–material interface [5, 421]. In addition, hydrogel coatings can incorporate endothelial adhesion ligands, pro-endothelialization growth factors, or nitric oxide donors to preferentially promote endothelial cell adhesion, spreading, and maturation while limiting abnormal smooth muscle cell proliferation. By regulating molecular diffusion and release kinetics, such coatings may achieve a temporally programmed transition from early antiproliferative effects to later pro-endothelialization functions, more closely matching the natural sequence of vascular healing [422].

Inflammatory regulation is equally important for stent coatings. As foreign bodies, stents inevitably trigger local immune responses, and persistent inflammation can promote neointimal hyperplasia and restenosis [404, 415]. Hydrogel coatings may mitigate these effects through their soft and compliant physical properties and through the delivery of anti-inflammatory drugs, immunomodulatory molecules, or ROS-responsive components. Inflammation-responsive hydrogels, for example, can release anti-inflammatory agents more rapidly during the inflammatory phase while maintaining structural stability after inflammation decreases, thereby coordinating immune modulation with endothelial repair [423]. More recent design concepts have further shifted from simple drug release or anti-adhesion toward endothelial function biomimicry. By tuning surface charge, hydrophilicity, hydrophobicity, and nanoscale topology, hydrogel coatings can partially mimic features of the endothelial glycocalyx or basement membrane, supporting the formation of a regenerated endothelial layer that more closely resembles native vascular intima in structure and function [424].

#### Hydrogel-based cardiovascular sensing and prognostic monitoring

Long-term cardiovascular management increasingly depends on continuous monitoring of cardiac electrical activity, mechanical contraction, vascular dynamics, and local biochemical states. Conventional electrocardiography, wired electrodes, intermittent imaging, and short-term wearable devices can provide important information, but they are often limited by intermittent measurement, peripheral signal acquisition, motion artifacts, or insufficient ability to reflect localized myocardial pathology, especially in arrhythmia monitoring, myocardial ischemia assessment, postoperative remodeling, and long-term prognosis evaluation [163, 400, 425]. Myocardial sensor patches and hydrogel-based sensing interfaces have therefore attracted attention as flexible platforms that can directly attach to or integrate with cardiovascular tissues to achieve high spatiotemporal resolution and long-term monitoring.

A central challenge for myocardial sensor patches is interfacial mismatch. Myocardial tissue is highly hydrated, rhythmically beating, and continuously subjected to tensile, compressive, and shear deformation. Conventional rigid or low-hydration sensing materials are prone to interfacial debonding, stress concentration, signal drift, and foreign-body responses, which can compromise long-term signal reliability [163, 426]. Hydrogels can address this challenge by providing tissue-like softness, stretchability, stress relaxation, and conformal adhesion. By tuning elastic modulus, network dynamics, and adhesive chemistry, hydrogel interfaces can maintain stable contact with myocardium during repetitive cardiac cycles and reduce interfacial shear stress [426, 427]. For example, Deng et al. developed a stretchable and strongly adhesive conductive hydrogel patch that maintained stable contact under continuous cardiac motion, produced lower interfacial shear stress than conventional flexible electrodes, and enabled long-term low-noise electrocardiographic recording [427]. These findings indicate that mechanical compliance is a fundamental requirement for reliable myocardial sensing rather than merely an auxiliary material property.

Electrical coupling is another critical design factor. Cardiac electrical signals have low amplitudes and are highly susceptible to noise; therefore, signal quality depends strongly on tissue–electrode interfacial impedance. Dry electrodes or rigid conductive layers can undergo dehydration, impedance increase, and signal attenuation in wet physiological environments. Conductive hydrogels overcome this limitation by incorporating conductive polymers, ionic conductors, or carbon-based nanomaterials into hydrated polymer networks, thereby establishing continuous ion–electron coupling pathways and reducing interfacial impedance [428].

For long-term implantation or surface-adhered monitoring, biocompatibility and interfacial stability also determine clinical feasibility. Myocardial and vascular tissues are sensitive to chronic mechanical friction and foreign-body stimulation, and unstable interfaces may induce inflammation, fibrotic encapsulation, signal attenuation, or device failure [163]. Hydrogels can reduce mechanical irritation because of their low modulus and high water content; in addition, surface chemistry, degradation behavior, and incorporation of anti-inflammatory or antioxidant components can be adjusted to maintain a more stable biological interface. Dynamically crosslinked self-healing hydrogels can autonomously restore structural continuity after microcrack formation or fatigue damage, thereby extending the functional lifetime of sensor patches and reducing monitoring interruption caused by interfacial failure [41].

As cardiovascular care moves from acute event management toward long-term risk assessment and individualized prognosis, hydrogel-based monitoring systems are expected to capture not only electrical or mechanical signals, but also biochemical indicators associated with disease progression. Local inflammation, oxidative stress, endothelial dysfunction, and metabolic changes may occur before overt macroscopic abnormalities become apparent [429, 430]. By incorporating responsive motifs sensitive to pH, ROS, inflammation-associated enzymes, or metabolic markers, hydrogels can convert microenvironmental changes into detectable physical, electrical, or optical outputs, thereby extending cardiovascular monitoring from outcome-based indicators to process-oriented indicators [430]. Inflammation-responsive hydrogels may further integrate sensing with therapeutic regulation by releasing anti-inflammatory factors during periods of heightened inflammatory activity while maintaining structural stability after inflammation subsides, creating a synergistic relationship between monitoring and intervention [431].

Hydrogel-based cardiovascular sensing is also moving toward multimodal and data-driven platforms. By integrating strain-sensitive, temperature-responsive, biochemical recognition, and conductive modules within a single material system, hydrogels can support simultaneous monitoring of cardiac electrical activity, mechanical contraction, tissue deformation, and local metabolic or inflammatory states [30]. Soft hydrogel-based electronic systems developed by the Rogers group illustrate this direction, in which materials and devices are designed together to acquire multiple cardiac parameters stably over time [432]. In the broader context of prognostic management, hydrogels can function as front-end material nodes that bridge biological tissues, bioelectronic devices, and algorithmic analysis models, enabling continuous data acquisition for individualized cardiovascular risk assessment and clinical decision-making [433].

Overall, hydrogel-based cardiovascular strategies are evolving from localized repair materials toward integrated platforms for tissue reconstruction, vascular interface regulation, physiological sensing, and prognostic monitoring. In myocardial repair, hydrogels provide mechanical support, localized biochemical modulation, cell or extracellular vesicle delivery, and vascularization-promoting environments. In stent coatings, they regulate blood–material interactions, endothelialization, inflammation, and biomimetic vascular interface formation. In sensing and prognosis, hydrogels offer soft, wet, conductive, adhesive, and responsive interfaces capable of long-term signal acquisition and microenvironmental monitoring.

### Immune-mediated inflammatory disease modulation

Autoimmune and immune-mediated inflammatory diseases (IMIDs), including rheumatoid arthritis (RA), systemic lupus erythematosus (SLE), multiple sclerosis (MS), systemic sclerosis (SSc), and psoriasis, are characterized by impaired immune tolerance, persistent inflammatory activation, and progressive destruction or remodeling of target tissues [434, 435]. These disorders usually follow a chronic and relapsing course and are associated with highly complex lesion-specific microenvironments. Clinically, their management relies largely on long-term immunosuppressive therapies or biologic agents; however, systemic administration is frequently limited by adverse effects, rapid drug metabolism, high dosing requirements, and variable therapeutic efficacy. Moreover, target tissues such as articular cartilage, kidney, central nervous system tissue, fibrotic skin, and inflamed epidermis often exhibit structural fragility and limited regenerative capacity under chronic inflammatory stimulation, making pharmacological intervention alone insufficient to simultaneously achieve immune control and tissue repair [436]. Because gastrointestinal inflammatory diseases, such as ulcerative colitis and inflammatory bowel disease, also involve persistent mucosal inflammation, immune dysregulation, oxidative stress, and barrier dysfunction, representative hydrogel strategies for these diseases are included in Table 6 for comparative discussion.

**Table 6:** Representative hydrogel-based strategies for gastrointestinal and immune-mediated inflammatory diseases

Diseases	Biomaterial	Active substances	Advantages	References
Ulcerative colitis	Furfural-functionalized chitosan-mannose polymer; 3-maleimide hydroxypropyl-β-cyclodextrin, HPCD-AMI	Rhubarb-derived nanovesicles; Kaempferol	Colon-targeted, macrophage-targeted, synergistic therapy	[437]
	Methacrylate-grafted hyaluronic acid/o-nitrobenzyl alcohol-terminated tetra-arm polyethylene glycol	Curcumin-loaded poly(lactic-co-glycolic acid) microspheres	Ultra-fast gelation, long adhesion	[438]
	Sodium alginate; Chitosan	Curcumin	Nano-in-micro colon-targeting delivery	[439]
	Sodium alginate	Tumor necrosis factor alpha small interfering RNA	Efficient oral targeting	[440]
	Methacrylated hyaluronic acid	Polydopamine-coated selenium nanoparticles	Multifunctional colon-targeting oral delivery	[441]
	Diselenide-bridged arctigenin-chitosan conjugate	Arctigenin	ROS-responsive targeting	[442]
Inflammatory bowel disease	Zein and sodium alginate	Curcumin and zinc Prussian blue analog	Multi-mechanism synergistically regulates intestinal homeostasis	[443]
	Hyaluronic acid/gelatin hydrogel	Curcumin	Colon long-lasting	[444]
	Dopamine-modified hyaluronic acid	/	Oral self-crosslinking, strong adhesion, barrier repair	[445]
	Carboxylated chitosan-carboxymethyl cellulose sodium	Titanium carbide nanosheets	Cross-linker-free precise targeting	[446]
Rheumatoid arthritis	Deep Eutectic Solvent Hydrogel (main components: Arginine, Citric Acid, Carbomer 940)	Nanoceria (active substance), Methotrexate (loaded drug)	Noninvasive transdermal delivery, synergistic immuno-chemotherapy	[447]
	Tripolycerol monostearate	Psoralen, Calcium peroxide	Metabolic-driven, bio-responsive, multi-target	[448]
	Azide-functionalized hyaluronic acid, PLA-b-PEG-N3 polymeric aggregates	Dexamethasone	NO-responsive sequential dual-drug release	[449]
	Thiol-modified hyaluronic acid and dual-gas-mediated crosslinker	Methotrexate	Regulating gas microenvironment, synergistic therapy	[450]
Multiple sclerosis	Hyaluronic acid-thiol, Heparin-thiol, beta-cyclodextrin maleimide, 8-arm polyethylene glycol adamantane	HBD-RGD5 (Platelet-derived growth factor-AA and recombinantly designed bidomain peptide HBD-RGD5)	Enhances cell survival, promotes remyelination	[451]
	Chitosan-poly(lactic-co-glycolic acid)	Glatiramer acetate	Long-acting sustained release reduces injection reactions	[452]
	Poloxamer; Chitosan; Hyaluronic acid	Glatiramer acetate	Sustained release, reduced injection frequency	[453]
	Chitosan and derivatives	Oligodendrocyte progenitor cells	Nose-to-brain non-invasive remyelination	[454]
	Hyaluronic acid; 4-arm polyethylene glycolamine	/	Hydrogel material itself anti-inflammatory, modulates ECM	[455]
Systemic lupus erythematosus	Hyaluronic acid methacrylate	Monocyte chemotactic protein-1 and interleukin-4	Macrophage enrichment and polarization	[456]
	Generation 3 polylysine dendrimer–disulfide crosslinked nanogel	Dexamethasone	Renal targeting DNA scavenging	[457]
	Polylysine peptide dendrimer and alginate	Human mesenchymal stem cells	Cell encapsulation and immunomodulation	[458]
	Sodium alginate–poly-D-lysine	Human mesenchymal stem cells	Bowel-adherent long survival	[459]

Hydrogels offer a materials-based strategy for IMIDs because they can provide localized and sustained delivery, microenvironment-responsive release, immune-cell modulation, and tissue-supportive functions within diseased sites. Their injectability, conformability, adhesiveness, tunable degradation behavior, and responsiveness to inflammatory cues such as pH, ROS, nitric oxide (NO), hydrogen sulfide (H_2_S), enzymes, or cell-free DNA (cfDNA) allow therapeutic activity to be spatially confined and temporally regulated. Accordingly, hydrogel systems in autoimmune diseases are no longer designed merely as passive drug carriers, but increasingly as immunoregulatory platforms that integrate drug delivery, macrophage reprogramming, oxidative stress regulation, gasotransmitter balance, cell protection, and tissue repair. The following sections summarize representative hydrogel strategies in RA, SLE, MS, SSc, and psoriasis, with an emphasis on how material design is adapted to distinct immune microenvironments and tissue-damage patterns (Table 6).

#### Rheumatoid arthritis and synovial immune microenvironment regulation

RA is an autoimmune disease characterized by chronic synovial inflammation and immune-mediated destruction of articular cartilage and bone. Clinically, it manifests as joint swelling, pain, stiffness, and progressive functional loss. Its pathological hallmarks include immune activation involving T cells, B cells, macrophages, synovial fibroblasts, and inflammatory cytokines, which together drive synovial hyperplasia, cartilage degradation, and bone erosion [460]. Although conventional disease-modifying antirheumatic drugs (DMARDs), biologics, and other anti-inflammatory therapies can alleviate symptoms and slow disease progression, long-term systemic treatment is often associated with adverse effects and limited capacity for tissue repair [461]. Therefore, hydrogel-based strategies for RA have mainly focused on localized intervention within inflamed joints, prolonged retention of therapeutic agents, and remodeling of the synovial inflammatory microenvironment.

One major design direction is macrophage targeting and reprogramming. Pro-inflammatory macrophages are central effectors in RA, and their persistent activation amplifies cytokine release, synovial inflammation, and bone destruction. Hydrogels can improve the local enrichment of anti-inflammatory drugs, nucleic acids, or immunomodulatory agents while limiting systemic exposure. Siddiqui et al. designed a folate-decorated chitosan–chondroitin sulfate nanoparticle-loaded hydrogel carrying leflunomide, exploiting folate receptor-β overexpression on infiltrating macrophages in arthritic joints. This system enhanced transdermal penetration and joint accumulation, improved inflammatory cytokine profiles, and reduced systemic adverse effects compared with free leflunomide or non-targeted nanoparticles [462]. Li et al. further developed an intra-articular nano–microplex system, MTAsi@MG, in which TNF-α siRNA-loaded artesunate prodrug lipoplexes were immobilized on methacrylated hyaluronic acid microspheres. This HA microsphere hydrogel mimicked the synovial environment, protected the gene/drug complex from rapid clearance, lubricated the joint cavity, and suppressed TNF-α and IL-1β expression, macrophage infiltration, synovial hyperplasia, and histological damage in collagen-induced arthritis (CIA) rats [463].

Hydrogels can also exploit the acidic and hypoxic RA synovial microenvironment for responsive release and combination therapy. Wu et al. reported a pH-sensitive peptide hydrogel encapsulating methotrexate and bismuthene nanosheet/PEI, which collapsed under acidic synovial conditions to release methotrexate and simultaneously enabled photodynamic and photothermal elimination of hyperplastic fibroblast-like synoviocytes (FLS), thereby modulating both inflammatory macrophages and stromal pathological components [464]. In a subsequent design, the same group developed a microenvironment-responsive peptide hydrogel co-loading MTX-PEI/siRNA polyplexes and BiNS/MTX-PEI. This system released therapeutic components more rapidly at mildly acidic pH, silenced CD86, NF-κB, and p38 MAPK-related pathways in dendritic cells, macrophages, and synovial fibroblasts, and disrupted the inflammatory feedback loop between macrophages and FLS in CIA rats [465]. Complementing these ligand- and pathway-targeted systems, Song et al. constructed a double-crosslinked alginate hydrogel containing Zn^2+^-crosslinked alginate and hyperbranched poly(β-amino ester) nanospheres loaded with TNF-α siRNA. This formulation downregulated TNF-α and GLUT1, suppressed glycolysis in M1 macrophages, and used Zn^2+^ to promote PPARγ-associated fatty acid oxidation and M2 polarization, thereby correcting macrophage immunometabolism and attenuating synovitis and cartilage damage [466].

Another important therapeutic target in RA is the oxidative and hypoxic microenvironment at the synovium–bone interface. Conventional DMARDs and biologics are not well suited to directly correct the coexistence of excessive ROS and hypoxia, which contributes to cartilage degradation, bone erosion, impaired stem cell function, and osteoblast dysfunction [467]. Zhao et al. developed a hyaluronic acid-based nanozyme-reinforced self-protective hydrogel in which ε-poly-L-lysine-modified MnCoO nanozymes with catalase-like activity were anchored within a dynamically crosslinked HA network together with BMSCs. This hydrogel used excessive H_2_O_2_ in RA lesions as a substrate, reducing ROS levels while generating oxygen to relieve hypoxia. In an RA rabbit model combined with a 3D-printed porous titanium alloy scaffold, this system reduced inflammatory mediators, oxidative damage markers, and osteoclast-related signals while improving new bone formation, osseointegration, and mechanical fixation [264]. Li et al. developed a noninvasive transdermal strategy using a deep eutectic solvent-enhanced hydrogel containing mesoporous silica nanoparticles co-loaded with methotrexate and nano-CeO_2_. The Ce^3+^/Ce^4+^ redox cycle scavenged ROS, promoted macrophage polarization from an M1 phenotype toward an M2 phenotype, and synergized with methotrexate to reduce synovial inflammation and arthritis severity without obvious skin irritation or major organ toxicity [447].

Gasotransmitter regulation has further expanded the microenvironmental design logic of RA hydrogels. Excessive NO in inflamed synovium and periarticular bone promotes oxidative/nitrosative stress, cytokine amplification, and osteoclast activation, whereas systemic NO depletion may disrupt physiological vascular and immune functions [468]. Kim et al. designed an in situ-forming M-NO hydrogel based on an azide-functionalized HA network and an NO-cleavable crosslinker. This system scavenged excess NO, softened and swelled in an NO-dependent manner, and released dexamethasone on demand. In CIA models, intra-articular dexamethasone-loaded M-NO hydrogel reduced clinical arthritis scores, paw swelling, inflammatory cytokines, and bone erosion more effectively than free drug or non-responsive controls [449]. Geng et al. developed a self-healing HA-based DNRS hydrogel integrating NO scavenging with on-demand H_2_S delivery. In RA-like environments, this hydrogel consumed excessive NO, released H_2_S in response to cysteine, and accelerated methotrexate release. The system reprogrammed macrophages from CD86^+^ M1 toward CD206^+^ M2 phenotypes, suppressed NF-κB-related inflammatory signaling, enhanced osteoblast activity, inhibited osteoclastogenesis, restored NO/H_2_S balance, and protected bone in CIA rats [450].

Overall, current hydrogel-based RA strategies mainly act on downstream effector-phase pathology rather than directly correcting upstream immune tolerance failure. Macrophage-targeted systems, ROS-scavenging nanozyme or CeO_2_ hydrogels, and NO/H_2_S-responsive matrices can alleviate cytokine amplification, oxidative/nitrosative stress, synovial hyperplasia, and bone erosion, thereby improving local joint inflammation and structural damage.

#### Systemic autoimmune disease platforms: systemic lupus erythematosus and multiple sclerosis

SLE is a systemic autoimmune disease involving multiple organs and systems, with core pathological processes including loss of immune tolerance, autoantibody and immune-complex production, complement activation, and widespread inflammation [469]. Clinically, SLE can affect the skin, joints, kidneys, central nervous system, and cardiovascular system, presenting as cutaneous rashes, arthralgia, lupus nephritis, neuropsychiatric symptoms, and relapsing–remitting disease progression [470]. Standard treatments, including glucocorticoids, antimalarial agents, immunosuppressants, and biologics, are effective for controlling disease activity, but long-term systemic use increases the risk of infection, metabolic complications, and organ toxicities, while their capacity to reverse established tissue damage or achieve organ-specific intervention remains limited.

In SLE, hydrogel systems have been primarily developed as immunoregulatory platforms rather than simple drug carriers. One important strategy is to protect and potentiate mesenchymal stem cells (MSCs). MSC therapy is attractive because MSCs can regulate T cells, B cells, dendritic cells, NK cells, and macrophages by secreting immunomodulatory factors such as IL-10, TGF-β, TSG-6, PGE_2_, and HGF, and by promoting regulatory T-cell expansion. However, conventional systemic MSC administration is limited by rapid clearance, short persistence, phenotypic instability, and exposure to hostile inflammatory and humoral environments. Hydrogel-based microencapsulation provides MSCs with ECM-like niches that support nutrient exchange while protecting them from large immune components. Nie et al. designed adhesive porous alginate/poly-D-lysine microparticles for MSC encapsulation using microfluidic electrospray and sacrificial leaching. These microparticles prolonged MSC residence after intraperitoneal administration, enhanced expression of immunomodulatory genes, promoted anti-inflammatory macrophage polarization, reduced anti-dsDNA antibody titers and renal immune-complex deposition, and improved renal pathological outcomes in MRL/lpr lupus-prone mice [459]. Zhu et al. developed ECM-inspired peptide dendrimer/alginate microgels with tunable porosity, stiffness, and selective permeability. These ALG/G3K microgels excluded macromolecules such as BSA and IgG while permitting diffusion of small nutrients and signaling molecules, thereby supporting human UC-MSC survival and immunoregulatory function. Subcutaneous transplantation of MSC-laden microgels reduced mesangial proliferation, glomerular IgG/C3 deposition, anti-dsDNA antibodies, renal dysfunction markers, and systemic IL-6, with viable MSCs still detectable at the injection site one month later [458].

Macrophage recruitment and reprogramming provide another hydrogel-based strategy for restoring immune homeostasis in SLE. Zhu et al. developed HAMA hydrogel microparticles co-encapsulating monocyte chemoattractant protein-1 (MCP-1) and IL-4. The design aimed to recruit inflammatory macrophages and subsequently drive their transition from a pathogenic M1 phenotype toward a pro-resolving M2 phenotype. In vitro, MCP-1 promoted macrophage migration, while IL-4-containing formulations, particularly MCP-1/IL-4 dual-loaded particles, increased CD206^+^ macrophages and reduced inflammatory cytokines. In MRL/lpr mice, intraperitoneal administration of these microparticles prolonged cytokine retention, promoted local anti-inflammatory macrophage polarization, reduced splenomegaly, anti-dsDNA antibody levels, BUN, glomerular mesangial hyperproliferation, and IgG/C3 deposition, and improved systemic inflammatory cytokine profiles without obvious major-organ toxicity [456].

For lupus nephritis, excessive ROS and cfDNA act together to drive innate immune activation, TLR9 signaling, chronic inflammation, and renal damage. Zhu et al. developed a peptide dendrimer-based nanogel (G3DSP) formed by crosslinking third-generation poly-L-lysine dendrimers with a disulfide-containing linker. The cationic nanogel could bind anionic cfDNA, suppress CpG-induced TLR9 activation, and undergo ROS-responsive degradation. In inflammatory macrophages, G3DSP scavenged ROS, promoted M1-to-M2 phenotypic transition, reduced TNF-α and IL-6, and increased Arg-1 and IL-10. When loaded with dexamethasone, the resulting DXM@G3DSP nanogel achieved ROS-triggered drug release and preferential accumulation in inflamed kidneys. In MRL/lpr mice, weekly administration reduced serum cfDNA, anti-dsDNA antibodies, renal dysfunction markers, systemic cytokines, and renal immune-complex deposition, while improving lupus nephritis pathology without detectable hepatotoxicity or nephrotoxicity [457]. These studies suggest that hydrogels and nanogels can act as organ-targeted platforms for cell delivery, macrophage reprogramming, ROS/cfDNA clearance, and controlled immunosuppression in SLE.

MS represents another autoimmune disease context in which hydrogels have been explored for both therapeutic delivery and disease modeling. MS is a chronic inflammatory demyelinating disease of the CNS characterized by immune-mediated damage to myelin and oligodendrocytes, leading to lesions in the brain and spinal cord and impaired neural signal transmission [471, 472]. Although disease-modifying therapies such as interferon-beta, glatiramer acetate, and monoclonal antibodies can reduce relapse frequency or modulate immune responses, they are not always sufficient to halt progression or promote remyelination, and they can be associated with immune suppression, infection risk, liver toxicity, or poor treatment adherence [473, 474].

One hydrogel strategy in MS is to improve existing drug delivery. Glatiramer acetate has a short half-life and requires frequent subcutaneous injection, which can cause injection-site pain, inflammation, lipoatrophy, and reduced adherence. Shobeirean et al. developed a thermosensitive in situ gelling system based on Poloxamer 407 modified with chitosan and hyaluronic acid to formulate a long-acting glatiramer acetate depot. The formulation remained injectable at room temperature and rapidly gelled at 37 °C after administration, forming a localized reservoir with extended near-zero-order release over 168 h. In mice, this system reduced early cytokine and biomarker exposure peaks while maintaining immunomodulatory activity over a longer period, suggesting a more convenient long-acting dosing strategy for MS [453].

Hydrogels can also serve as disease-modeling tools for MS. Conventional demyelination models often induce global tissue damage and do not fully recapitulate focal lesions surrounded by intact CNS tissue. Eigel et al. developed PEGDA-based cylindrical cryogel scaffolds for spatially restricted lysophosphatidylcholine delivery in brain or spinal cord slice cultures. These macroporous cryogels enabled local toxin release, produced focal demyelination while preserving adjacent tissue, and allowed spontaneous remyelination to be observed over time. This platform preserves lesion–normal tissue interfaces and provides a hydrogel-engineered model for studying oligodendrocyte progenitor cell recruitment, focal demyelination, remyelination, and screening of pro-remyelinating compounds [475].

A third direction is hydrogel-assisted cell delivery for remyelination. In chronic MS lesions, endogenous oligodendrocyte progenitor cells may be present but fail to differentiate effectively because of inflammation and abnormal matrix cues. Transplantation of human oligodendrocyte progenitor cells has potential for replacement remyelination, but conventional needle injection exposes cells to shear stress and leads to substantial acute cell death and poor engraftment. Podder et al. developed a shear-thinning hydrogel based on host–guest interactions and covalent crosslinking among hyaluronic acid, heparin, PEG-adamantane, and β-cyclodextrin components. By incorporating heparin-binding RGD peptides and PDGF-AA, the hydrogel provided mechanical protection during injection and bioactive ECM-like cues for OPC survival. In vivo, this platform improved human OPC retention, enhanced MBP^+^ myelin formation, and restored node of Ranvier structures in hypomyelinated mouse models, demonstrating the potential of injectable hydrogels for cell-based remyelination therapy [451].

Taken together, hydrogel strategies for systemic autoimmune diseases are still at an early stage but show clear potential. In SLE, hydrogels mainly function as immunoregulatory niches or responsive nanogels for MSC protection, macrophage reprogramming, ROS/cfDNA scavenging, and organ-targeted immunosuppression. In MS, hydrogels can optimize existing drug delivery, create focal demyelination models, and protect transplanted OPCs for remyelination. These approaches indicate that hydrogel systems can support not only immune suppression, but also disease modeling, cell protection, and tissue repair in systemic autoimmune disorders.

#### Fibrotic and inflammatory skin diseases: systemic sclerosis and psoriasis

Beyond RA, SLE, and MS, hydrogel-based strategies have gradually expanded to autoimmune and immune-mediated diseases characterized by fibrosis, vascular injury, or skin barrier dysfunction, with SSc and psoriasis representing two distinct but clinically important examples. SSc is an immune-mediated rheumatic disease involving microvascular abnormalities, immune activation, and progressive fibrosis affecting the skin and multiple internal organs, including the lungs, gastrointestinal tract, and cardiovascular system [476]. Psoriasis, by contrast, is a chronic immune-mediated skin disease driven largely by the IL-23/IL-17 axis and characterized by keratinocyte hyperproliferation, epidermal thickening, inflammation, and barrier disruption [477]. In these conditions, hydrogels are mainly used to localize cell or vesicle therapies, enhance retention in diseased tissues, modulate inflammatory and fibrotic microenvironments, and support barrier repair.

In SSc, conventional therapies, including immunosuppressants, targeted biologics, antifibrotic agents, and in selected cases autologous hematopoietic stem cell transplantation, can partially alleviate symptoms and delay organ deterioration but are generally insufficient to reverse the vasculopathy–inflammation–fibrosis cycle. MSCs have attracted attention because of their immunomodulatory, pro-angiogenic, and antifibrotic potential, yet their clinical efficacy is limited by rapid death or clearance after injection into inflamed and fibrotic tissues. To address this limitation, Nie et al. developed an injectable self-healing hydrogel constructed from N,O-carboxymethyl chitosan and four-arm benzaldehyde-terminated PEG through reversible Schiff-base linkages. The hydrogel could be injected through a fine needle, gel in situ, self-heal, degrade controllably, and support three-dimensional expansion of clinical-grade umbilical cord MSCs. More importantly, the hydrogel microenvironment reshaped the MSC secretome, upregulating immunoregulatory and pro-angiogenic factors such as TSG-6, CCL-2, COX-2, PGE_2_, and HGF, while prolonging MSC retention in subcutaneous tissue. In a bleomycin-induced mouse model of skin fibrosis, MSC-loaded hydrogels improved body weight and skin thickness, reduced dermal collagen deposition, downregulated fibrosis-associated markers including Col1A, Col3A, α-SMA, and TGF-β, increased the Mmp1/Timp1 ratio, and preserved subcutaneous adipose architecture, supporting the potential of MSC-laden hydrogels for SSc-related skin fibrosis [478].

In psoriasis, current therapies such as topical corticosteroids, retinoids, phototherapy, systemic immunosuppressants, and biologics can relieve erythema, scaling, and pruritus but are often associated with recurrence, prolonged maintenance requirements, systemic adverse effects, and adherence challenges. Recent hydrogel strategies increasingly focus on combining extracellular vesicles or natural nanovesicles with topical or injectable hydrogels to provide local reservoirs for sustained immunomodulation and barrier repair. Kalarikkal et al. isolated plant-derived nanovesicles from garlic and green onion and incorporated them into a thermosensitive Pluronic F127 hydrogel for topical delivery. In an imiquimod-induced psoriasis model, this PDNV hydrogel reduced epidermal hyperproliferation, restored near-normal skin architecture, decreased Ki-67 and NF-κB expression, and upregulated differentiation-associated keratins. Mechanistically, the system activated NRF2 signaling and suppressed IL-17-mediated inflammatory pathways, while the F127 hydrogel improved local retention and transdermal delivery [479]. Chen et al. further explored the gut–skin axis by using outer membrane vesicles derived from the commensal bacterium *Parabacteroides goldsteinii* and encapsulating them in a PF127 thermosensitive hydrogel for subcutaneous administration. Compared with live bacterial therapy, these non-proliferative vesicles retained immunomodulatory components while avoiding concerns associated with live microbes. In imiquimod-induced psoriasis, PF127–Pg OMV hydrogel reduced skin inflammation and epidermal thickening, suppressed NF-κB, TNF-α, IL-1β, IL-6, IL-17A, IL-23, and S100A8/A9, and showed favorable systemic safety [480].

Overall, hydrogels in SSc and psoriasis illustrate two complementary directions for immune-mediated tissue disease therapy. In fibrotic disease, hydrogels can protect and potentiate MSCs, enhance their retention, and amplify their immunomodulatory, pro-angiogenic, and antifibrotic secretome. In inflammatory skin disease, thermosensitive or topical hydrogels can localize natural vesicles near lesions, sustain their release, suppress IL-23/Th17–IL-17 inflammatory signaling, reduce oxidative and inflammatory networks, and support epidermal differentiation and barrier recovery. These strategies expand the application of hydrogels from classical drug delivery toward local immune-tissue microenvironment remodeling.

Overall, hydrogel-based systems provide versatile platforms for autoimmune and immune-mediated inflammatory diseases by enabling localized, sustained, and microenvironment-responsive interventions. Despite the clinical heterogeneity of RA, SLE, MS, SSc, and psoriasis, several shared design principles are emerging. Hydrogels improve the retention and stability of small molecules, biologics, nucleic acids, cytokines, stem cells, and vesicle-based therapeutics, thereby reducing systemic exposure and increasing local activity. They also actively modulate pathological microenvironments by reprogramming macrophage phenotypes, scavenging ROS or cfDNA, regulating gasotransmitters such as NO and H_2_S, relieving hypoxia, supporting angiogenesis, promoting remyelination, or suppressing fibrotic and inflammatory signaling. In cell-based strategies, hydrogels provide protective ECM-mimetic niches that improve the survival and function of MSCs or oligodendrocyte progenitor cells in otherwise hostile inflammatory tissues.

### Reproductive tissue repair and fertility-related applications

Infertility, commonly defined as the inability to achieve a clinical pregnancy after 12 months or more of regular unprotected sexual intercourse, affects an estimated 15%–20% of reproductive-aged couples worldwide and has become an increasing public health concern across diverse socioeconomic regions [481, 482]. As a multifactorial condition, infertility may arise from pathological factors in either or both partners. In women, coordinated function of the ovaries, fallopian tubes, uterus, and endometrium is essential for ovulation, fertilization, embryo implantation, and pregnancy maintenance. Disorders such as intrauterine adhesions (IUAs), endometriosis, chronic endometritis, uterine fibroids, pelvic inflammatory disease, and age-related ovarian decline can impair fertility by disrupting hormonal signaling, damaging the endometrial interface, or generating inflammatory and fibrotic reproductive microenvironments [483]. Male infertility is similarly heterogeneous and may result from varicocele, testicular injury, obstructive abnormalities, reproductive tract infection, endocrine dysregulation, oxidative stress-mediated sperm damage, or vascular and stromal dysfunction, which together affect sperm production, quality, motility, transport, or sexual function.

Current clinical approaches to infertility and reproductive disorders mainly include hormonal therapy, surgical intervention, and assisted reproductive technologies (ART) [484]. Although these strategies have greatly improved reproductive medicine, they often do not fully restore the structural and functional integrity of reproductive tissues. In women, hormonal therapy can regulate ovulation or correct endocrine imbalance, but it has limited ability to repair structural injury or reverse chronic inflammation and fibrosis. Surgical procedures, including hysteroscopic adhesiolysis, myomectomy, and laparoscopic excision of endometriotic lesions, can restore anatomical continuity but may be complicated by recurrence, postoperative adhesions, infection, and impaired endometrial receptivity. In men, hormonal replacement, microsurgical repair, and antioxidant or anti-inflammatory regimens can improve selected conditions, but many forms of male infertility or sexual dysfunction arise from local microenvironmental abnormalities, such as fibrosis, chronic inflammation, vascular insufficiency, and oxidative stress, which are difficult to correct with conventional therapies [485]. ART, including in vitro fertilization and intracytoplasmic sperm injection, provides alternative routes to conception, but it does not directly repair the underlying pathology of reproductive tissues and remains constrained by cost, accessibility, psychological burden, ethical concerns, and the intrinsic quality of gametes and reproductive microenvironments.

Therefore, effective reproductive tissue repair requires more than anatomical correction or endocrine regulation. It also requires local modulation of inflammation, fibrosis, oxidative stress, vascularization, ECM remodeling, and gamete- or embryo-supportive microenvironments. In this context, hydrogel-based strategies are increasingly being explored as localized platforms for reproductive tissue reconstruction, bioactive factor delivery, cellular or vesicle-based therapy, and noninvasive biomarker detection. The following sections summarize representative applications in male reproductive disorders and female reproductive tissue repair (Table 7).

**Table 7:** Representative hydrogel-based strategies for reproductive tissue repair and fertility-related applications

Diseases	Biomaterial	Active substances	Advantages	References
Vaginal tissue repair	Gelatin methacryloyl; Carrageenan	Mg^2+^	Rapid repair	[486]
	Hyaluronic acid hydrogel	Umbilical cord mesenchymal stem cell exosomes	Non-hormonal therapeutic strategy	[487]
Endometrial repair	Phenylboronic acid-modified methacrylated hyaluronic acid and polyvinyl alcohol composite dual-network hydrogel	Platelet-rich plasma	Locational activation and sustained release	[488]
	poly(N-isopropylacrylamide)-grafted bacterial cellulose	*Lactobacillus*	Long-term retention	[489]
Ovarian tissue repair	Fibrin hydrogel	Nitric oxide-releasing nanoparticles	Alleviating ischemia in transplanted ovaries	[490]
	PEG-vinyl sulfone hydrogel with electrospun dextran fibers	Basement membrane binder peptide	High follicle survival	[491]
Erectile dysfunction	Acrylic acid, gelatin, polyethylene glycol diacrylate (PEGDA, 6 kDa), lithium phenyl 2,4,6-trimethylbenzoylphosphinate (LAP), and tartrazine	/	Achieved erectile function in vitro and restored reproductive ability in vivo	[492]
	Gelatin methacryloyl	Adrenomedullin	Mechano-therapy for ED	[493]
Testicular injury	Hyaluronic acid methacrylate and gelatin methacrylate	Exosomes preloaded with lycopene	Dual-stage delivery system, synergistic antioxidative and anti-apoptotic effects	[494]
Prostate disease	Chitosan-β-glycerophosphate disodium salt	Emodin	Triple-target rectal sustained release	[495]
	Polyethylene glycol diacrylate	Urinary exosomal miRNA	Highly sensitive non-invasive early diagnosis	[496]

#### Male reproductive tissue repair and diagnostic applications

In male reproductive disorders, hydrogels have been explored for erectile dysfunction (ED), testicular injury, prostatitis, and prostate cancer-related diagnosis. These conditions involve distinct anatomical sites but share several pathological features, including vascular dysfunction, oxidative stress, inflammation, fibrosis, impaired ECM organization, and insufficient local retention of therapeutic agents. Accordingly, hydrogel-based systems have been designed to provide biomimetic tissue support, local drug or vesicle delivery, antioxidative and anti-inflammatory microenvironment regulation, and sensitive biomarker detection.

ED is closely associated with endothelial dysfunction, smooth muscle apoptosis, fibrosis, impaired nitric oxide signaling, and abnormal penile hemodynamics [497, 498]. Conventional treatments, including oral phosphodiesterase-5 inhibitors, intracavernosal injection, vacuum erection devices, and penile prosthesis implantation, mainly alleviate symptoms by hemodynamic modulation or mechanical replacement, but they rarely reconstruct damaged cavernosal architecture or restore the microvascular and sinusoidal networks required for physiological erectile function [499]. This limitation is particularly evident in severe organic ED or cavernosal defects caused by trauma or tumor resection, where prosthesis implantation may require removal of residual cavernosal tissue and may be associated with infection, mechanical failure, and foreign-body sensation [500].

To address these structural and functional challenges, hydrogel-based strategies have been developed to reconstruct biomimetic corpus cavernosum systems. One representative example is a 3D-printed penile perfusion model constructed using a photocurable gelatin-based composite hydrogel ink. Through high-resolution 3D printing, the authors fabricated a biomimetic cavernosal model containing sinusoidal and microvascular channels, which was further combined with a high-strength tunica albuginea-mimicking layer. This hydrogel system reproduced key hemodynamic events of erection in vitro, including arterial inflow, cavernosal expansion, and venous occlusion. When used as an implantable scaffold in rabbit and porcine cavernosal defect models, it supported neovascular and sinusoidal tissue ingrowth, induced low inflammatory responses, was gradually replaced by newly formed cavernosal tissue, improved cavernosal pressure and blood-flow parameters, and increased pregnancy rates after mating [492]. This study highlights the value of 3D-printed hydrogel scaffolds in structural reconstruction and functional restoration of severe cavernosal defects.

Another important mechanism in ED is matrix stiffening and fibrosis, especially in metabolic or age-related disease. To investigate and regulate this process, mechanically tunable GelMA hydrogels have been used to construct in vitro and in vivo microenvironments with defined stiffness. By culturing cavernosal smooth muscle cells on GelMA substrates with different elastic moduli, researchers demonstrated that matrix stiffening promotes a fibrotic phenotype through YAP/TAZ nuclear translocation and upregulation of collagen deposition- and contraction-related genes. Based on this mechanobiological insight, injectable or in situ-gelling GelMA scaffolds were applied in ED animal models to soften the local microenvironment, suppress YAP/TAZ activation, reduce cavernosal fibrosis and vascular wall remodeling, and restore nitric oxide signaling and erectile responses [493]. This work suggests that hydrogels can serve not only as delivery matrices, but also as mechanically instructive platforms that regulate pathological signaling in reproductive tissues.

For testicular injury and male infertility, current treatments are often limited to removal of toxic exposures, hormonal intervention, and empirical antioxidant or anti-inflammatory therapy. These approaches frequently suffer from poor targeting, short drug half-life, insufficient local concentration, and limited ability to reconstruct the spermatogenic microenvironment. Testicular injury caused by toxins, oxidative stress, torsion, trauma, varicocele-related hypoxia, or chemotherapy is commonly associated with ROS accumulation, inflammation, apoptosis, and disruption of seminiferous tubule architecture [501]. Ru and colleagues developed a HAMA-GelMA composite hydrogel loaded with lycopene-containing adipose-derived mesenchymal stem cell exosomes to treat deoxynivalenol-induced testicular injury. In this system, exosomes served as natural nanocarriers for lycopene and also provided stem cell-derived immunoregulatory and regenerative signals, while the photocrosslinkable HAMA-GelMA hydrogel created an ECM-like, mechanically tunable, and sustained-release microenvironment. In vitro, the hydrogel enhanced spermatogenic cell viability, migration, and proliferation and reduced H_2_O_2_-induced ROS levels. In vivo, the HAMA-GelMA@Exo-Lyc system restored testicular and epididymal weight, sperm count, motility, survival rate, and testosterone levels, reduced oxidative stress and inflammatory markers, suppressed mitochondria-mediated apoptosis, and improved seminiferous tubule structure and spermatogenic stratification [494]. This example demonstrates how hydrogel-based vesicle delivery can coordinate antioxidative, anti-inflammatory, anti-apoptotic, and tissue-restorative effects in testicular injury.

Hydrogels have also shown value in prostate-related disorders, including chronic nonbacterial prostatitis and prostate cancer diagnosis. Chronic nonbacterial prostatitis is difficult to treat because most cases lack a clearly identifiable pathogen, and symptoms are often driven by persistent inflammation, oxidative stress, nociceptive sensitization, and stromal remodeling. Conventional anti-inflammatory agents, α-receptor blockers, and antioxidants often exhibit limited efficacy due to poor penetration across the prostatic barrier and unstable local drug concentrations. To improve local retention, Zhang and colleagues developed a thermosensitive rectal hydrogel based on a chitosan matrix containing emodin-based triple-targeting nanoparticles. After perianal administration, the hydrogel gelled at body temperature to form an adhesive depot for sustained periprostatic release. Through anti-inflammatory, antioxidant, and immunomodulatory effects, this system suppressed NF-κB activation, reduced ROS accumulation and inflammatory mediators, improved prostate histopathology, and alleviated pain hypersensitivity [495].

In prostate cancer, hydrogels have been applied not only for therapy but also for noninvasive diagnosis. Although PSA testing is widely used, its low specificity can lead to false positives, overdiagnosis, and unnecessary invasive biopsies. Urinary exosomal miRNAs are promising biomarkers because they are stable and can reflect the status of the urinary tract and prostate, but their low abundance in urine limits conventional detection methods. Kim and colleagues developed PEGDA hydrogel microparticles integrated with hybridization chain reaction amplification for urinary exosomal miRNA analysis. The barcoded and region-specific functionalization of hydrogel microparticles confined target-specific probes within a three-dimensional architecture, while HCR provided more than 30-fold signal amplification. This platform enabled detection of miR-6090 and miR-3665 from less than 1 mL of urine, and ratio-based analysis distinguished prostate cancer patients from healthy individuals [496]. This study illustrates the potential of hydrogel microstructures as diagnostic platforms for sensitive biomarker amplification and noninvasive reproductive cancer screening.

Overall, hydrogel-based strategies for male reproductive disorders are moving from simple local delivery toward multifunctional platforms for tissue reconstruction, microenvironment regulation, and diagnostic amplification. In ED, hydrogels can reconstruct cavernosal architecture, regulate matrix stiffness, promote angiogenesis, reduce fibrosis, and restore nitric oxide-related function. In testicular injury, hydrogels provide ECM-like niches for exosome or antioxidant delivery, attenuate oxidative stress and inflammation, and support spermatogenic tissue recovery. In prostate-related diseases, thermosensitive hydrogels improve local drug retention for prostatitis, while hydrogel microparticles enhance the sensitivity of urinary exosomal miRNA detection for prostate cancer. These applications demonstrate the capacity of hydrogels to address vascular, parenchymal, stromal, and diagnostic needs in male reproductive medicine.

#### Female reproductive tissue repair and fertility restoration

Female reproductive disorders involve highly coordinated but vulnerable tissues, including the vagina, endometrium, uterus, and ovaries. Vaginal injury or atrophy, endometrial damage, IUA, chronic inflammation, and ovarian dysfunction can impair fertility and reproductive health through epithelial damage, fibrosis, altered ECM structure, insufficient vascularization, immune imbalance, and hormonal disruption [502, 503]. Conventional treatments such as hormone therapy, topical medication, surgical intervention, and ovarian tissue cryopreservation can partially improve symptoms or preserve fertility, but they often fail to provide sustained local repair, prevent recurrence, or restore a physiologically supportive microenvironment. Hydrogels are therefore increasingly investigated as localized regenerative platforms for vaginal tissue repair, endometrial regeneration, and ovarian function restoration.

For vaginal tissue repair, conventional hormone therapy, topical agents, and surgery may alleviate symptoms but are limited by poor drug penetration, unstable local drug concentration, and inadequate reconstruction of damaged tissue, particularly in vaginal atrophy, radiation-induced injury, and postpartum tissue damage [504]. Wang and colleagues developed a GelMA/carrageenan hydrogel scaffold incorporating bioactive Mg^2+^ ions for reproductive tract defect repair. By modulating Mg^2+^ release, this hydrogel promoted vaginal epithelial cell proliferation and migration, exhibited favorable mechanical strength and biocompatibility, reduced inflammation, and accelerated tissue repair in vivo [486]. In another strategy, hyaluronic acid hydrogel was used to deliver umbilical cord mesenchymal stem cell-derived exosomes for vaginal atrophy in an ovariectomized rat model. The hydrogel prolonged local exosome retention and supported epithelial cell proliferation, migration, and differentiation, thereby improving vaginal epithelial thickness and repairing damaged tissue architecture without significant side effects [487]. These studies suggest that hydrogel-based non-hormonal delivery systems may provide a useful alternative for vaginal tissue regeneration.

Endometrial injury and IUA represent major causes of impaired fertility because they damage the endometrial basal layer, reduce receptivity, and promote fibrotic remodeling. Current treatments, including hysteroscopic adhesiolysis, local drug delivery, and physical barriers, can temporarily restore uterine cavity morphology but are frequently associated with recurrence, limited regeneration, and insufficient recovery of endometrial function [505]. Hydrogels have been used to prolong the local activity of regenerative agents and reshape the endometrial microenvironment. Qi et al. developed a double-network hydrogel for localized activation and delivery of platelet-rich plasma (PRP). Although PRP contains multiple growth factors that promote tissue repair, its clinical efficacy is constrained by instability and short duration of action. Incorporation into the hydrogel prolonged PRP retention and enabled sustained release of growth factors and cytokines, thereby promoting endometrial repair and reducing IUA formation more effectively than PRP alone [488]. Fan et al. proposed a probiotic-based strategy by loading lactobacilli into a thermosensitive poly(N-isopropylacrylamide)-grafted bacterial cellulose hydrogel. The hydrogel improved local retention and biocompatibility, while lactobacilli modulated the local immune microenvironment, promoted macrophage polarization from a pro-inflammatory M1 phenotype toward a reparative M2 phenotype, reduced fibrosis, increased endometrial thickness, and promoted endometrial regeneration without significant side effects [489]. These examples demonstrate that endometrial hydrogels can extend beyond structural filling to regulate growth factor retention, microbiota-related immune modulation, fibrosis, and tissue receptivity.

Ovarian repair and fertility preservation face additional challenges because ovarian tissue is highly sensitive to ischemia, oxidative stress, and disruption of follicular microenvironments. After ovarian injury or transplantation, insufficient angiogenesis can lead to ischemic damage and follicle loss, limiting functional recovery. Yang et al. developed a fibrin hydrogel containing nitric oxide-releasing nanoparticles to improve vascularization of transplanted ovaries. The hydrogel provided a local matrix for sustained NO release, promoted angiogenesis, improved follicle survival, and enhanced ovarian function after transplantation [490]. In parallel, Nason-Tomaszewski et al. explored ECM-templated fibrous hydrogels for ovarian tissue remodeling. By using electrospun dextran fibers functionalized with ECM-sequestering peptides, this system retained cell-secreted ECM, promoted cell aggregation and matrix deposition, and improved oocyte survival and growth by restoring critical cell–matrix interactions [491]. These studies highlight the importance of reconstructing vascular and ECM cues for ovarian tissue engineering.

Overall, hydrogel-based strategies for female reproductive disorders mainly focus on restoring local tissue structure and improving the regenerative microenvironment. In vaginal repair, hydrogels enhance local retention of bioactive ions or exosomes, promote epithelial regeneration, and reduce inflammation. In endometrial injury and IUA, hydrogels prolong the activity of PRP, probiotics, or other regenerative agents, regulate immune responses, reduce fibrosis, and support endometrial reconstruction. In ovarian repair, hydrogels promote angiogenesis, follicle survival, ECM remodeling, and functional recovery after injury or transplantation. Across these applications, hydrogels provide localized and tissue-adaptive platforms that can support fertility-preserving medicine by combining structural support, bioactive delivery, and microenvironmental modulation.

Taken together, reproductive system diseases illustrate the broader value of hydrogels in tissues where fertility depends on fine coordination among structure, vascularization, immune balance, ECM organization, endocrine signaling, and gamete or embryo-supportive microenvironments. In male reproductive disorders, hydrogels can contribute to cavernosal reconstruction, testicular protection, prostatitis treatment, and prostate cancer biomarker detection. In female reproductive disorders, hydrogels can support vaginal tissue repair, endometrial regeneration, adhesion prevention, and ovarian tissue preservation.

### Emerging applications in ophthalmic and endocrine/metabolic diseases

In addition to wound repair, musculoskeletal disorders, cancer, neurological diseases, cardiovascular diseases, immune-mediated inflammatory diseases, and reproductive system disorders, hydrogels have also shown increasing potential in several other clinically relevant disease contexts. These conditions often involve delicate tissue structures, dynamic physiological interfaces, long-term disease monitoring requirements, or systemic metabolic dysregulation, thereby imposing distinct demands on biomaterial design. For example, ophthalmic applications require materials with high transparency, ocular surface compatibility, strong but gentle adhesion, and sustained local residence, whereas endocrine and metabolic diseases such as diabetes require systems capable of continuous monitoring, intelligent drug release, and intervention against chronic complications. With continued advances in responsive polymer networks, microneedle systems, adhesive hydrogels, nanocomposite hydrogels, and bioelectronic interfaces, hydrogel-based platforms are progressively expanding from conventional therapeutic scaffolds toward integrated systems for tissue repair, disease monitoring, controlled delivery, and complication management (Table 8).

**Table 8:** Representative hydrogel-based strategies for ophthalmic and endocrine/metabolic diseases

Diseases	Biomaterial	Active substances	Advantages	References
Dry eye syndrome	Oxidized hyaluronic acid-containing aldehyde groups and gelatin	Polyethylene imine-functionalized carbon dots nano-enzymes	Combined antioxidative and anti-inflammatory activity, prolonged ocular surface retention	[506]
	balafilcon A silicone hydrogel	/	Long-lasting hydration & corneal protection	[507]
	Silk fibroin	Cyclosporine A	Strong bioadhesion, sustained release	[508]
	Silk fibroin nanoparticle–calcium alginate	CP-99,994	Long-acting anti-inflammatory repair	[509]
	Hyaluronic acid	Cyclosporine	Ultrasoft elastic, long retention	[510]
Diabetic nephropathy	Chitosan	Taxifolin	Enhanced bioavailability and renal protection	[511]
	Free-radical cross-linked ROS-responsive hydrogel shell	Recombinant adropin protein (34–76 fragment)	ROS-triggered release-renal protection	[512]
Eye injury	Gelatin Methacryloyl, Hyaluronic acid Glycidyl Methacrylate, Polyethylene Glycol Diacrylate	/	High adhesion, photocrosslinkable, easy application	[513]
	Alginate-Gly-Leu-Lys-Dopamine	/	Dual-network, ion-activated, robust adhesion, promotes regeneration, clinically applicable	[514]
	4-arm-polyethylene glycol-N-hydroxysuccinimide + lysozyme	/	Strong sealing, transparent, safe	[515]
	Porcine corneal ECM–peptide-modified alginate–microbial transglutaminase	/	Transparent-strong-adhesion-astigmatism-free	[516]
Diabetes	Gelatin Methacryloyl, Carboxymethyl cellulose-poly(2-hydroxyethyl acrylate) nanogel	Glucose Oxidase	Fast, minimally invasive, blood-free detection	[517]
	Crosslinked dextran-methacrylate	Fungal FAD-glucose dehydrogenase–phenanthroline quinone mediator	10-day continuous monitoring	[518]
	Dopamine-Hyaluronic Acid hydrogel, Poly(3,4-ethylenedioxythiophene): Polystyrene Sulfonate	Silver Nanoparticles, Platinum Nanoparticles	Enzyme-less, real-time, conductive sensing	[519]
	Polyacrylamide–polydopamine interpenetrating network	Glucose oxidase–selenium nanoparticles	High-sensitivity-fast-response-stable	[520]

#### Ophthalmic tissue repair and ocular surface disease management

Ocular tissues are highly delicate, transparent, and structurally specialized, and their clinical treatment is often challenged by limited local drug bioavailability, repeated administration requirements, slow healing, infection risk, and the need to preserve optical and biomechanical function. Hydrogels are particularly attractive for ophthalmic applications because they can mimic the hydrated ocular microenvironment, provide tissue-compatible interfaces, and be engineered as ocular surface scaffolds, surgical sealants, lubricating materials, or sustained drug delivery systems [521–523]. Their high water-retention capacity and tunable adhesion allow them to interact with the corneal or conjunctival surface without causing excessive mechanical irritation, while their network structure can be used to prolong local drug residence and control the release of antibiotics, antioxidants, anti-inflammatory agents, or bioactive molecules.

In ocular trauma and corneal repair, hydrogel systems are mainly designed to replace or assist conventional suturing, seal defects, restore tissue integrity, and support transparent regeneration. Severe ocular injuries can lead to scar formation, corneal opacity, infection, and visual impairment, while open-globe injuries disrupt the full thickness of the ocular wall and compromise the eye’s natural protective barrier [524, 525]. To address this clinical need, Jumelle et al. developed a hydrogel adhesive patch composed of GelMA, glycidyl methacrylate-modified hyaluronic acid, and PEGDA. This material integrates tissue adhesion, viscosity for surgical positioning, flexibility, extensibility, and tunable degradation. It can be applied in a contact lens-like manner and rapidly cured in situ under visible light. In ex vivo porcine eye models, the patch sealed sharp lacerations, blunt trauma, and complex defects with localized tissue loss, demonstrating good conformability and wound closure performance [513].

Corneal stromal and epithelial defects require not only mechanical closure but also preservation of transparency, curvature, and regenerative coordination among corneal layers. Shen et al. constructed a double-network hydrogel based on porcine decellularized corneal stroma matrix gel and HAMA. The decellularized corneal matrix provided native tissue-like bioactivity, whereas HAMA improved shape retention and structural stability through chemical and photo-crosslinking. In a rabbit corneal stromal defect model, the transparent hydrogel adhered to the stromal bed, accelerated repair, and achieved near-complete defect closure within two weeks [516]. Zhao et al. further developed an ion-activated bioadhesive hydrogel composed of native corneal ECM and peptide-modified alginate. Gelation could be triggered using a calcium ion-containing contact lens, enabling rapid closure of large corneal defects and restoration of corneal curvature. Long-term follow-up in rabbits showed coordinated regeneration of the corneal epithelium, stroma, and nerves, with progressive recovery of transparency and repair outcomes comparable to donor corneal transplantation [514]. In addition, Zhang et al. reported a PEG-lysozyme hydrogel corneal adhesive for postoperative wound closure. This material exhibited tunable pore size, gelation time, pH, and modulus, showed favorable cytocompatibility, withstood burst pressures comparable to suture closure in ex vivo porcine eyes, and maintained stable intraocular pressure without leakage or material detachment in rabbit eyes [515]. Collectively, these studies indicate that hydrogel corneal adhesives are evolving from simple sealants toward transparent, bioactive, and regenerative ocular repair materials.

Dry eye disease represents another important ocular application scenario. It is a multifactorial ocular surface disorder characterized by discomfort, visual dysfunction, tear film instability, hyperosmolarity, persistent inflammation, and tissue damage [526]. Oxidative stress plays an important role in its initiation and progression, making local antioxidant and anti-inflammatory therapy a promising strategy [527]. Ou et al. developed an aldehyde-functionalized Pluronic F127 hydrogel eye drop for delivering ultrasmall Cu_2_₋_x_Se nanoparticles. These nanoparticles exhibited ROS-scavenging activity and enzyme-mimicking functions similar to superoxide dismutase and glutathione peroxidase, while the AF127 hydrogel improved ocular surface adhesion and residence time. In a mouse model of dry eye disease, the Cu_2_₋_x_Se NPs@AF127 hydrogel reduced corneal oxidative injury and improved disease manifestations without evident systemic toxicity [528]. Yang et al. developed a metal-free thermoresponsive antioxidant carbon dot hydrogel capable of scavenging multiple free radicals and exerting antioxidant, anti-inflammatory, and anti-apoptotic effects. In a dry eye mouse model, this hydrogel restored corneal epithelial integrity and thickness, supporting its therapeutic potential for ocular surface repair [529].

Immune dysregulation is also closely involved in dry eye disease, particularly in autoimmune-associated cases. Patients with autoimmune disorders show an increased incidence of dry eye disease, and aberrant activation of innate and adaptive immunity contributes to ocular surface inflammation [530]. Chen et al. developed a cell-delivery eye-drop system in which mesenchymal stem/stromal cells were encapsulated within porous RGD-modified alginate microcarriers. These microcarriers provided a biomimetic niche that supported MSC expansion, viability, and functional maintenance while enabling ocular surface administration. In an autoimmune dry eye mouse model, RGD-Alg@MSCs prolonged cell residence on the ocular surface, promoted tear secretion and corneal epithelial repair, and attenuated local inflammation by suppressing dendritic cell activation and Th17 differentiation [531]. This example highlights the potential of hydrogel microcarriers to transform ocular therapy from repeated topical drug administration toward cell-based immunomodulatory intervention.

Overall, hydrogel-based ophthalmic strategies are moving from passive lubrication or sealing toward multifunctional platforms for transparent tissue repair, ocular surface protection, sustained drug delivery, antioxidative therapy, and immune regulation. In corneal trauma, hydrogels can provide adhesive, transparent, and regenerative materials for wound closure and tissue integration. In dry eye disease, they can prolong ocular residence, scavenge ROS, suppress inflammation, and support epithelial repair.

#### Endocrine and metabolic disease management

Diabetes is one of the most common chronic endocrine and metabolic diseases worldwide and remains a major threat to human health. Its management requires not only regulation of blood glucose homeostasis but also timely prevention, diagnosis, and treatment of systemic complications. Conventional diabetes care relies heavily on fingertip blood glucose testing and repeated insulin injection, both of which can reduce patient adherence because of pain, inconvenience, and skin-related complications [532]. In recent years, hydrogel-based systems have been increasingly explored for diabetes management because they can integrate minimally invasive sampling, continuous monitoring, glucose-responsive signal transduction, sustained insulin delivery, and intervention against diabetes-associated tissue damage. These systems illustrate the shift of hydrogels from therapeutic materials toward closed-loop or semi-closed-loop platforms that connect sensing, delivery, and disease microenvironment regulation.

Hydrogel microneedles represent an important approach for minimally invasive glucose monitoring. Compared with conventional lancets, microneedles penetrate only shallow skin layers, enabling interstitial fluid sampling with reduced pain and lower risk of tissue irritation. Yan Wang and colleagues developed an intelligent microneedle patch consisting of a transparent photopolymerized base and pH- and glucose-responsive hydrogel microneedles. The hydrogel was composed of GelMA, pH-responsive nanogels, and glucose oxidase, and could respond rapidly and sensitively to physiological glucose concentrations through ionization and proton balance mechanisms. In diabetic mouse models, the microneedles extracted and responded to interstitial glucose, allowing quantitative glucose readings based on changes in microneedle height and swelling ratio [517]. Darmau and colleagues developed a crosslinked hydrogel microneedle-bioelectrochemical enzyme sensor array using dextran-methacrylate micromolds and dry-state visible-light crosslinking. This design enabled controlled microneedle geometry, improved durability, and continuous biomarker monitoring in artificial interstitial fluid for up to 10 days [518]. GhavamiNejad and colleagues further reported a low-cost hydrogel microneedle system based on swellable dopamine-hyaluronic acid hydrogel, Pt and Ag nanoparticles, and PEDOT:PSS-enhanced conductivity. This system enabled non-enzymatic electrochemical glucose detection in dermal interstitial fluid and accurately tracked glucose fluctuations in a type 1 diabetic rat model across hyperglycemic, normoglycemic, and hypoglycemic ranges [519].

Beyond microneedle swelling or electrochemical systems, hydrogel networks can also act as functional matrices for implantable or wearable glucose sensors. Glucose oxidase-based sensors typically convert glucose into measurable signals through enzymatic oxidation and H_2_O_2_ generation [533]. Lin Jiang and colleagues developed a GOx- and selenium nanoparticle-containing hydrogel based on a PDA conductive network. GOx generated H_2_O_2_ during glucose oxidation, while selenium nanoparticles amplified the electrochemical signal through cascade catalysis. This hydrogel showed favorable electrochemical performance and provided a stable material platform for glucose detection [520]. In addition to blood or interstitial fluid, tears and sweat offer opportunities for less invasive glucose monitoring. Kim and colleagues immobilized bimetallic nanocatalysts within the nanoporous hydrogel of smart contact lenses for continuous tear glucose monitoring. In diabetic rabbits, the sensor showed high sensitivity, rapid response, low detection limits, and strong agreement between tear glucose and blood glucose measurements, with additional safety and feasibility evaluation in human eyes [534]. Kanokpaka and colleagues developed a self-healing glucose-responsive hydrogel-based triboelectric sensor for sweat glucose monitoring. In this system, GOx encapsulated in β-cyclodextrin was embedded in a PVA matrix, and glucose oxidation triggered hydrogel swelling and conductivity changes. Coupling with a triboelectric nanogenerator enabled self-powered monitoring of sweat glucose changes before and after meals [535]. These studies show that hydrogels can serve as both sampling interfaces and signal-transducing materials in wearable, ocular, and self-powered diabetes monitoring platforms.

Hydrogel-based insulin delivery systems aim to reduce the burden of repeated injections and improve glycemic control through sustained or glucose-responsive release. Insulin is a peptide hormone essential for regulating glucose uptake and maintaining blood glucose homeostasis, but long-term subcutaneous injection can cause local complications and reduce compliance. Mandal et al. designed an injectable cyclodextrin-extended polyurethane/carboxymethyl cellulose hydrogel for sustained insulin delivery. The CMC component formed a stable three-dimensional network, improved insulin loading, and provided a diffusion barrier for sustained release. In diabetic mice, subcutaneous injection of insulin-loaded PU-co-CD/CMC hydrogel maintained glucose regulation for up to three days, compared with less than one hour for conventional insulin injection, without evident organ toxicity [536]. In addition to delivery, monitoring insulin dynamics is also important for personalized diabetes management. Kelley et al. developed a microneedle patch based on polycarboxybetaine zwitterionic hydrogel and a molecular-spring electrochemical sensor. This platform resisted sterilization-related inactivation and enabled continuous insulin monitoring in interstitial fluid in a type 1 diabetic rat model, with real-time electrochemical signals consistent with ELISA measurements [537, 538].

Smart glucose-responsive hydrogels further provide a basis for self-regulated insulin release. Maity et al. developed a silk fibroin-based glucose-responsive hydrogel functionalized with phenylboronic acid and containing GOx. The PBA groups endowed the hydrogel with glucose responsiveness, while the porous network enabled insulin encapsulation. Under hyperglycemic conditions, H_2_O_2_ generated by the glucose-sensing elements induced oxidative cleavage and hydrogel degradation, triggering insulin release. In type 1 diabetic rats, subcutaneous injection of this hydrogel sustained insulin release and maintained blood glucose within the physiological range for up to 36 h [539]. Together, these insulin delivery systems show how hydrogels can prolong insulin exposure, reduce injection frequency, and respond to metabolic cues, although further optimization is still required for long-term precision, safety, and integration with glucose monitoring devices.

Diabetes management also requires intervention against chronic complications, including diabetic retinopathy, diabetic kidney disease, and diabetic neuropathy, which are closely associated with vascular dysfunction, basement membrane thickening, altered permeability, inflammation, oxidative stress, and tissue-specific microenvironmental disruption. In diabetic retinopathy, retinal microenvironment dysfunction is a key driver of disease progression. The retinal microenvironment consists of retinal cells, immune cells, ECM, soluble molecules, and the retinal vasculature, all of which interact to maintain retinal homeostasis [540]. Yue Zhou and colleagues developed a glucose-responsive hydrogel system, Cu-PEI/siMyD88@GEMA-Con A, for delivering Cu-PEI/siMyD88 nanoparticles to retinal pigment epithelial cells. Cu-PEI nanoparticles acted as antioxidants, reduced ROS, inhibited RPE pyroptosis, and helped prevent blood-retinal barrier damage, while siMyD88 reduced inflammatory signaling and IL-18 production. In diabetic mouse models, this hydrogel reduced retinal edema, lowered diabetic retinopathy incidence, and helped maintain retinal homeostasis [540].

For diabetic kidney disease, hydrogel or nanogel systems have been explored to improve drug bioavailability and enable microenvironment-responsive release. Taxifolin is a natural flavonoid with antioxidant and anti-inflammatory properties, but its therapeutic application is limited by bioavailability issues [541]. Yingchun Zhao and colleagues developed a chitosan-modified quercetin liposome system that improved taxifolin absorption and attenuated renal injury by inhibiting the NF-κB/NLRP3/caspase-1/IL-1β pathway. In diabetic nephropathy mice, this system reduced fasting blood glucose, kidney index, histological injury, and inflammatory pathway activation [511]. Another strategy focused on adropin, a peptide hormone encoded by the Enho gene with potential regulatory roles in diabetes and its complications [542]. Researchers developed a ROS-responsive hydrogel nanocapsule carrying adropin for targeted release in the diabetic kidney disease microenvironment [543]. At a relatively low dose, Ad@Gel improved renal function more effectively than recombinant adropin alone, reduced kidney lipid deposition, and inhibited SEBP-1 and ADRP expression, suggesting that ROS-responsive hydrogel nanocapsules can enhance therapeutic efficiency in diabetic kidney disease [512].

Overall, hydrogel-based systems in endocrine and metabolic disease management are evolving toward integrated sensing-delivery-regulation platforms. In diabetes monitoring, hydrogel microneedles, conductive hydrogel sensors, smart contact lenses, and sweat-based triboelectric systems provide minimally invasive or noninvasive routes for continuous glucose detection. In insulin therapy, injectable and glucose-responsive hydrogels prolong insulin exposure and enable metabolic cue-triggered release. In diabetic complications, responsive hydrogels and nanogels can reshape retinal or renal inflammatory and oxidative microenvironments, protect tissue barriers, and improve local therapeutic efficiency.

Beyond the disease contexts discussed above, hydrogels have also shown potential in additional clinically relevant fields, as partially summarized in the tables. Across these emerging applications, hydrogels share a common design logic: they provide soft, hydrated, and engineerable material interfaces capable of local retention, controlled release, tissue integration, and microenvironmental regulation. As the biomedical scope of hydrogel materials continues to expand, their clinical translation will increasingly depend not only on experimental efficacy but also on manufacturing scalability, formulation standardization, sterilization, storage stability, regulatory classification, clinical validation, and economic feasibility. These issues are therefore discussed in the following section, with the aim of clarifying the key barriers and future directions for translating hydrogel technologies from laboratory research into clinically viable therapeutic and diagnostic strategies.

## Perspectives and clinical translation

**Fig. 4:**
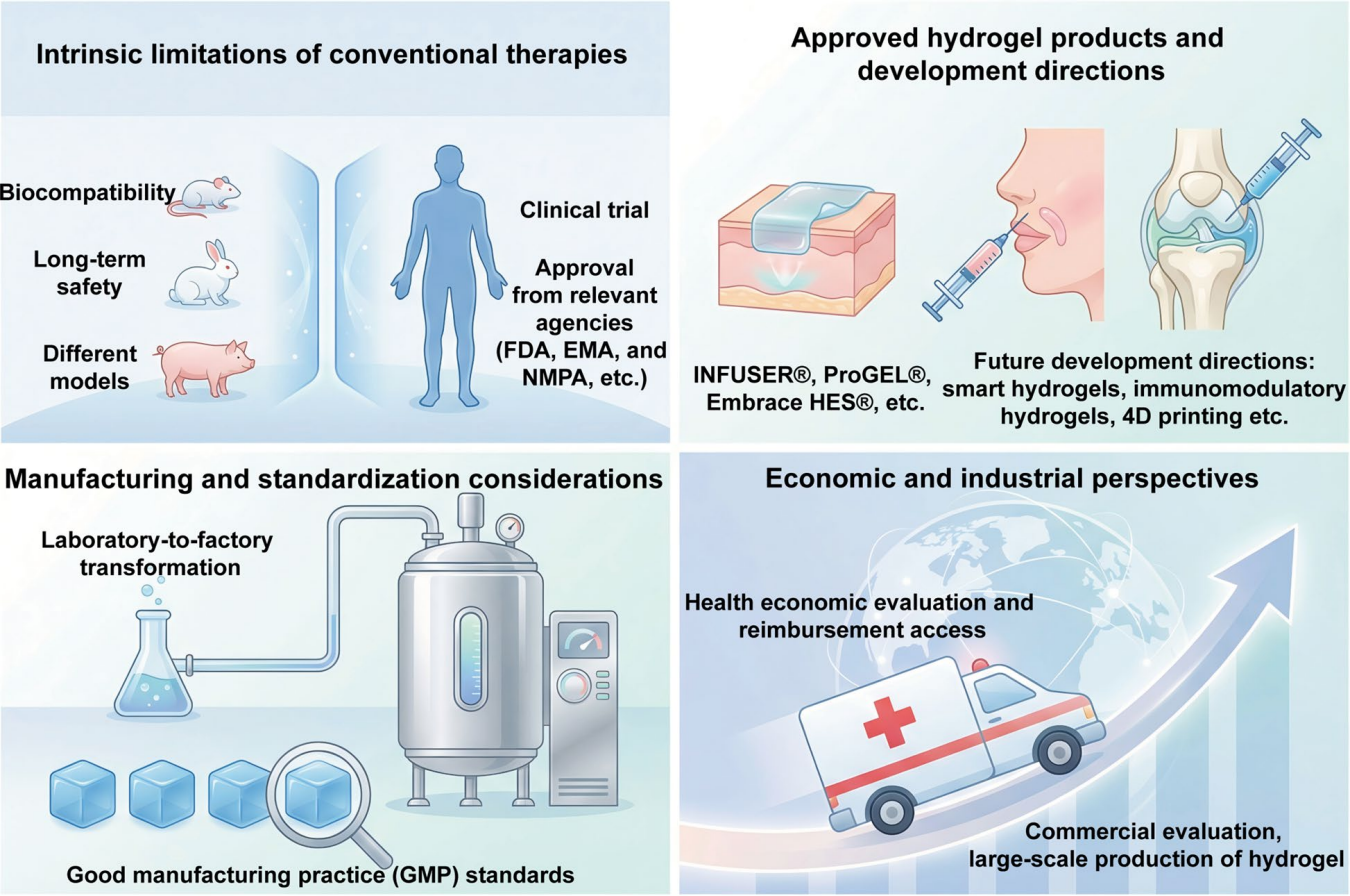
Schematic overview of key considerations for the clinical translation of hydrogel-based therapies. The figure summarizes the intrinsic limitations of conventional treatments, representative approved hydrogel products and emerging development directions, critical manufacturing and standardization requirements from laboratory to large-scale production, and economic and industrial perspectives including health economic evaluation and commercialization pathways. Created using Adobe Illustrator

With the rapid advances in hydrogel materials science, the potential of hydrogels in wound repair, drug delivery, tissue engineering, and the treatment of major diseases has been widely recognized. However, despite the compelling therapeutic outcomes achieved in laboratory studies, substantial barriers remain in translating hydrogel-based systems from bench to bedside. These challenges span multiple dimensions, including the verification of biosafety, controllability and robustness of functional performance, manufacturing scalability and standardization, regulatory approval pathways, and overall feasibility for industrialization. Accordingly, this section provides a forward-looking discussion of the clinical translation of hydrogels from four key perspectives: challenges in clinical translation, the current foundation of clinical applications, manufacturing and standardization considerations, and economic and industrial viewpoints (Fig. 4).

### Challenges in clinical translation

One of the primary challenges facing the clinical translation of hydrogels lies in the evaluation of biocompatibility and long-term safety. Although many hydrogel materials have demonstrated favorable cytocompatibility in vitro and promising short-term performance in animal studies, these results do not necessarily predict long-term safety in humans. After implantation or repeated administration, hydrogels may undergo swelling, mechanical fatigue, incomplete degradation, or unexpected interactions with proteins, immune cells, and surrounding tissues. These processes may lead to chronic inflammation, fibrotic encapsulation, foreign-body responses, delayed tissue remodeling, or local tissue compression. In addition, different hydrogel systems exhibit distinct degradation rates and degradation pathways, and the in vivo metabolism, accumulation, and potential toxicity of degradation products, residual crosslinkers, initiators, or functional nanocomponents require systematic long-term evaluation. These issues are particularly important for hydrogels intended for repeated administration, long-term implantation, or use in sensitive tissues such as the eye, nervous system, cardiovascular system, and reproductive organs.

A second important lesson from challenging translational attempts is that material performance in simplified experimental settings may not be maintained in clinically complex disease environments. Most preclinical hydrogel studies rely heavily on small-animal models with controlled lesion size, short observation windows, and relatively homogeneous pathological conditions. However, clinical patients often present with aging, diabetes, infection, vascular insufficiency, immune dysfunction, tumor heterogeneity, or chronic inflammation, all of which may alter hydrogel degradation, drug release, cell infiltration, vascularization, and immune responses. As a result, hydrogel-based therapies that show strong efficacy in animal models may exhibit reduced or inconsistent therapeutic effects in human settings. This translational gap is particularly evident in chronic wounds, cancer therapy, neurological repair, autoimmune diseases, and large-volume tissue regeneration, where the pathological microenvironment is highly dynamic and patient-specific.

The increasing complexity of hydrogel design also introduces new safety and controllability concerns. Many advanced hydrogels integrate drugs, growth factors, nucleic acids, extracellular vesicles, stem cells, immune cells, living microorganisms, conductive components, or nanomaterials. Although these strategies enhance multifunctionality, they also make it more difficult to define critical quality attributes and predict in vivo behavior. For example, uncontrolled or prolonged release of growth factors may induce ectopic tissue formation, aberrant angiogenesis, or excessive fibrosis, whereas insufficient release may result in poor therapeutic efficacy. Cell-laden hydrogels face additional challenges related to cell viability, phenotype stability, batch-to-batch variability, immune compatibility, and the potential risk of undesired differentiation or ectopic engraftment. Therefore, future translational studies should evaluate not only the hydrogel matrix itself, but also the stability, potency, dose, release profile, and biological fate of the incorporated active components.

Stimuli-responsive and smart hydrogels further highlight the need for more rigorous validation. Responsiveness to pH, ROS, enzymes, temperature, glucose, or inflammatory signals provides attractive opportunities for precision therapy, but disease-associated triggers in humans are often spatially and temporally heterogeneous. A response threshold optimized in vitro may not be appropriate in vivo, leading to premature activation, insufficient activation, or off-target release. In addition, repeated swelling-deswelling cycles, enzymatic degradation, or mechanical deformation may compromise long-term structural stability and release reproducibility. For hydrogel-based sensors and closed-loop therapeutic systems, unresolved issues such as biofouling, signal drift, calibration instability, interfacial mechanical mismatch, and integration with electronic or wireless modules must also be carefully addressed before clinical use.

Finally, the regulatory approval process remains a major barrier to clinical translation. Many functionalized hydrogels fall into the category of combination products, incorporating both a medical device component, such as a hydrogel scaffold, and a pharmaceutical or biological component, such as growth factors, small-molecule drugs, nucleic acids, extracellular vesicles, or cells. The regulatory classification of such products is inherently complex because they may need to satisfy requirements for devices, drugs, and biologics simultaneously. This complexity can prolong clinical trial timelines, increase validation costs, and raise uncertainty regarding product characterization, release criteria, sterilization, storage, and post-market surveillance. Establishing clearer regulatory strategies across different frameworks, including those of the FDA, EMA, and NMPA, is therefore essential for advancing hydrogel-based therapies.

Taken together, these lessons indicate that successful hydrogel translation requires a shift from proof-of-concept efficacy toward clinically realistic validation. Future studies should place greater emphasis on long-term safety, clinically relevant animal models, disease heterogeneity, standardized degradation and release testing, immune compatibility, reproducible manufacturing, and predefined performance criteria. Rather than increasing material complexity without clear clinical necessity, hydrogel design should be guided by specific unmet clinical needs, acceptable risk–benefit profiles, and feasible regulatory pathways.

### Approved hydrogel products and development directions

Despite the aforementioned challenges, hydrogels have already established a clinical foundation in several application areas. Currently approved or clinically used hydrogel products are mainly concentrated in scenarios with relatively simple structures, clearly defined functions, and favorable safety profiles, such as wound dressings, soft tissue fillers, tissue sealants, and localized tissue repair materials. Among these, natural polymer-based hydrogels, particularly hyaluronic acid-based systems, have been widely adopted in skin repair, soft tissue augmentation, ophthalmic applications, and postoperative adhesion prevention owing to their biocompatibility, injectability, and established clinical experience.

In the field of drug delivery, hydrogel systems have shown practical value by improving local retention and prolonging therapeutic exposure at target sites. Compared with systemic administration, localized hydrogel delivery can increase drug concentration within diseased tissues while reducing systemic adverse effects. This approach is particularly relevant to anticancer therapy, inflammatory disease management, metabolic regulation, and tissue repair. However, most clinically translated hydrogel products still rely on relatively simple material compositions and single-function mechanisms. More complex systems that integrate multiple drugs, biological factors, cells, nucleic acids, or responsive components remain largely at the preclinical or early translational stage, mainly because their safety, reproducibility, sterilization compatibility, and regulatory classification are more difficult to establish.

Looking forward, the development of hydrogel technologies is expected to move beyond conventional dressings, fillers, and passive depots toward more intelligent and integrated systems. One promising direction is AI-assisted hydrogel design. Because hydrogel performance is governed by highly coupled parameters, including polymer composition, crosslinking density, network structure, degradation kinetics, drug release behavior, and biological response, conventional trial-and-error optimization is often inefficient. Artificial intelligence and machine learning may help identify structure–property-function relationships, predict material behavior, and guide inverse design of hydrogel formulations according to specific clinical requirements. This direction will require standardized datasets, reproducible testing methods, and experimentally validated predictive models.

A second important direction is 4D printing of hydrogels. Unlike conventional three-dimensional printing, 4D printing introduces time-dependent changes in shape, structure, mechanics, degradation, or release behavior after fabrication. Such systems may be particularly useful for minimally invasive implantation, irregular defect repair, staged tissue regeneration, and dynamic disease microenvironment modulation. For example, a hydrogel construct could be delivered in a compact form and then expand, soften, stiffen, degrade, or release bioactive agents in response to physiological cues. The translational success of 4D-printed hydrogels will depend on the development of printable and biocompatible inks, predictable post-printing behavior, sterilization-resistant structures, and reliable in vivo performance.

A third emerging direction is the integration of hydrogels with closed-loop therapeutic systems. By combining sensing modules, signal-responsive networks, and controlled release functions, hydrogels may enable real-time monitoring and adaptive intervention. Representative examples include glucose-responsive hydrogels for diabetes management, pH- or ROS-responsive wound dressings, inflammation-responsive immunomodulatory systems, and hydrogel-based bioelectronic interfaces for tissue repair and physiological monitoring. These systems may transform hydrogels from passive therapeutic materials into interactive platforms capable of responding to disease progression. Nevertheless, closed-loop hydrogel systems also introduce additional challenges, including sensor drift, calibration stability, biofouling, power supply, data reliability, long-term biocompatibility, and complex regulatory pathways for material-device-drug combination products.

Overall, approved hydrogel products demonstrate that clinical translation is most feasible when material composition, function, safety, and manufacturing processes are clearly defined. Future development should therefore balance innovation with translational practicality. AI-assisted design, 4D printing, and closed-loop therapeutic systems provide concrete directions for next-generation hydrogels, but their clinical implementation will require stronger standardization, long-term validation, and closer integration among materials science, clinical medicine, engineering, and regulatory science.

### Manufacturing and standardization considerations

The clinical application of hydrogels depends not only on their biological functionality but also critically on their manufacturability and degree of standardization. During large-scale production, key parameters such as mechanical properties, degradation behavior, drug-loading efficiency, and release kinetics are often strongly coupled, such that even minor variations in a single parameter may lead to pronounced changes in overall performance. Therefore, the establishment of stable and well-controlled fabrication processes constitutes a fundamental prerequisite for successful clinical translation.

At present, there is still a lack of unified standards for hydrogel performance testing and evaluation across different research groups, which to some extent complicates cross-platform comparison and regulatory assessment. In the future, the development of more consensus-driven testing frameworks and quality standards, tailored to the critical performance requirements of hydrogels in specific clinical applications, is expected to improve their translational feasibility and overall clinical credibility.

Another major technical challenge in hydrogel manufacturing lies in sterilization. Hydrogels typically contain high water content and exhibit structural sensitivity to external conditions, rendering conventional high-temperature and high-pressure sterilization prone to inducing polymer network collapse, while high-energy irradiation methods, such as gamma irradiation, may compromise the integrity of encapsulated bioactive components, including proteins or peptides. Consequently, the development of mild terminal sterilization strategies or the implementation of fully aseptic processing approaches represents a key step toward ensuring both sterility and functional integrity of hydrogel products.

Finally, to enable efficient production and meet clinical application requirements, hydrogel fabrication processes must comply with Good Manufacturing Practice (GMP) standards. This necessitates not only high reproducibility and controllability of the manufacturing process but also the avoidance of harmful byproducts during production to ensure product safety. Accordingly, optimizing and adapting fabrication workflows to accommodate these stringent requirements during scale-up remains an essential step in facilitating the broader clinical adoption of hydrogel-based technologies.

### Economic and industrial perspectives

From an industrialization standpoint, hydrogel materials exhibit broad application potential in the biomedical field; however, their commercialization pathways require rational evaluation in the context of specific use scenarios. Hydrogel products with relatively simple structures, well-defined functions, and clear clinical needs are generally more amenable to large-scale manufacturing and market adoption. In contrast, highly integrated or structurally complex hydrogel systems may be better suited for high-value applications targeting diseases with significant unmet clinical needs, where their added functional sophistication can be more readily justified.

Another critical consideration lies in health economic evaluation and reimbursement access. Compared with conventional low-cost wound dressings or oral pharmaceuticals, emerging hydrogel-based therapies are often associated with higher upfront costs. To achieve market acceptance and inclusion in reimbursement systems, developers must not only demonstrate safety and efficacy, but also provide robust health economic evidence showing that these products can reduce overall healthcare expenditures, for example by shortening hospitalization duration, decreasing complication rates, or lowering disease recurrence.

Overall, the industrial translation of hydrogels requires coordinated efforts among academic researchers, industry stakeholders, and regulatory authorities. Through interdisciplinary collaboration and close integration with clinical practice and industrial development, hydrogel technologies are expected to establish stable and sustainable commercialization pathways in targeted disease treatments and precision medicine applications.

## Conclusion

In this review, we systematically summarize current biomedical applications of hydrogels, with a particular focus on the construction of hydrogel systems and the strategies by which customized biomedical functions can be imparted to these systems. We first outline the design principles and material engineering of hydrogels, including polymer chemistry and crosslinking strategies, tunable mechanical properties and degradation behavior, stimuli-responsive and smart hydrogels, biocompatibility, bioactivity, and functionalization strategies, as well as emerging manufacturing methods. We then review the current biomedical applications of hydrogels, encompassing drug delivery systems, tissue engineering and regenerative medicine, biosensing and diagnostics, and cell culture and organoid systems. Subsequently, we place emphasis on recent research progress of hydrogels in a wide range of disease contexts, including wound healing, musculoskeletal system repair, cancer therapy, neurological diseases, cardiovascular diseases, autoimmune disorders, and reproductive system diseases. Finally, we discuss the potential of hydrogels in clinical translation and outline future development directions. Overall, with the continued advances in life sciences, materials science, and regenerative medicine, hydrogels offer new research avenues and therapeutic strategies for diverse clinically relevant diseases. They show considerable potential in extending patient survival and improving quality of life, and are expected to contribute to the development of more effective therapeutic options in the future.

## Data Availability

Not applicable.

## References

[1] Fang W, Yang M, Wang L, Li W, Liu M, Jin Y, et al. Hydrogels for 3d bioprinting in tissue engineering and regenerative medicine: Current progress and challenges. Int J Bioprint. 2023;9(5):759. 10.18063/ijb.759. 10.18063/ijb.759 PMC10339415 37457925

[2] Lu P, Ruan D, Huang M, Tian M, Zhu K, Gan Z, et al. Harnessing the potential of hydrogels for advanced therapeutic applications: Current achievements and future directions. Signal Transduct Target Ther. 2024;9(1):166. 10.1038/s41392-024-01852-x. 38945949 10.1038/s41392-024-01852-x PMC11214942

[3] Ayoubi-Joshaghani MH, Seidi K, Azizi M, Jaymand M, Javaheri T, Jahanban-Esfahlan R, et al. Potential applications of advanced nano/hydrogels in biomedicine: Static, dynamic, multi-stage, and bioinspired. Adv Funct Mater. 2020;30(45):2004098. 10.1002/adfm.202004098.

[4] Wang Z, Wei H, Huang Y, Wei Y, Chen J. Naturally sourced hydrogels: Emerging fundamental materials for next-generation healthcare sensing. Chem Soc Rev. 2023;52(9):2992–3034. 10.1039/d2cs00813k. 37017633 10.1039/d2cs00813k

[5] Li J, Mooney DJ. Designing hydrogels for controlled drug delivery. Nat Rev Mater. 2016. 10.1038/natrevmats.2016.71. 29657852 10.1038/natrevmats.2016.71 PMC5898614

[6] Mikhail AS, Morhard R, Mauda-Havakuk M, Kassin M, Arrichiello A, Wood BJ. Hydrogel drug delivery systems for minimally invasive local immunotherapy of cancer. Adv Drug Deliv Rev. 2023;202:115083. 10.1016/j.addr.2023.115083. 37673217 10.1016/j.addr.2023.115083 PMC11616795

[7] Bakshi S, Sahoo PK, Li K, Johnson S, Raxworthy MJ, Krauss TF. Nanophotonic and hydrogel-based diagnostic system for the monitoring of chronic wounds. Biosens Bioelectron. 2023;242:115743. 10.1016/j.bios.2023.115743. 37826878 10.1016/j.bios.2023.115743

[8] Herrmann A, Haag R, Schedler U. Hydrogels and their role in biosensing applications. Adv Healthc Mater. 2021;10(11):e2100062. 10.1002/adhm.202100062. 33939333 10.1002/adhm.202100062 PMC11468738

[9] Liu H, Wang Y, Cui K, Guo Y, Zhang X, Qin J. Advances in hydrogels in organoids and organs-on-a-chip. Adv Mater. 2019;31(50):e1902042. 10.1002/adma.201902042. 31282047 10.1002/adma.201902042

[10] Tolabi H, Davari N, Khajehmohammadi M, Malektaj H, Nazemi K, Vahedi S, et al. Progress of microfluidic hydrogel-based scaffolds and organ-on-chips for the cartilage tissue engineering. Adv Mater. 2023;35(26):e2208852. 10.1002/adma.202208852. 36633376 10.1002/adma.202208852

[11] Lou J, Mooney DJ. Chemical strategies to engineer hydrogels for cell culture. Nat Rev Chem. 2022;6(10):726–44. 10.1038/s41570-022-00420-7. 37117490 10.1038/s41570-022-00420-7

[12] Prince E, Kumacheva E. Design and applications of man-made biomimetic fibrillar hydrogels. Nat Rev Mater. 2019;4(2):99–115. 10.1038/s41578-018-0077-9.

[13] Klionsky DJ, Petroni G, Amaravadi RK, Baehrecke EH, Ballabio A, Boya P, et al. Autophagy in major human diseases. EMBO J. 2021;40(19):EMBJ2021108863. 10.15252/embj.2021108863. 10.15252/embj.2021108863 PMC8488577 34459017

[14] Tian YE, Cropley V, Maier AB, Lautenschlager NT, Breakspear M, Zalesky A. Heterogeneous aging across multiple organ systems and prediction of chronic disease and mortality. Nat Med. 2023;29(5):1221–31. 10.1038/s41591-023-02296-6. 37024597 10.1038/s41591-023-02296-6

[15] Halliwell B. Understanding mechanisms of antioxidant action in health and disease. Nat Rev Mol Cell Biol. 2024;25(1):13–33. 10.1038/s41580-023-00645-4. 37714962 10.1038/s41580-023-00645-4

[16] Li H, Yang Y, Hong W, Huang M, Wu M, Zhao X. Applications of genome editing technology in the targeted therapy of human diseases: Mechanisms, advances and prospects. Signal Transduct Target Ther. 2020;5(1):1. 10.1038/s41392-019-0089-y. 32296011 10.1038/s41392-019-0089-y PMC6946647

[17] Borgo C, D’Amore C, Sarno S, Salvi M, Ruzzene M. Protein kinase ck2: A potential therapeutic target for diverse human diseases. Signal Transduct Target Ther. 2021;6(1):183. 10.1038/s41392-021-00567-7. 33994545 10.1038/s41392-021-00567-7 PMC8126563

[18] Tambuyzer E, Vandendriessche B, Austin CP, Brooks PJ, Larsson K, Miller Needleman KI, et al. Therapies for rare diseases: Therapeutic modalities, progress and challenges ahead. Nat Rev Drug Discov. 2020;19(2):93–111. 10.1038/s41573-019-0049-9. 31836861 10.1038/s41573-019-0049-9

[19] Manzari MT, Shamay Y, Kiguchi H, Rosen N, Scaltriti M, Heller DA. Targeted drug delivery strategies for precision medicines. Nat Rev Mater. 2021;6(4):351–70. 10.1038/s41578-020-00269-6. 34950512 10.1038/s41578-020-00269-6 PMC8691416

[20] Smith DA, Beaumont K, Maurer TS, Di L. Relevance of half-life in drug design. J Med Chem. 2018;61(10):4273–82. 10.1021/acs.jmedchem.7b00969. 29112446 10.1021/acs.jmedchem.7b00969

[21] Feng Y, Zhang Z, Tang W, Dai Y. Gel/hydrogel-based in situ biomaterial platforms for cancer postoperative treatment and recovery. Exploration (Beijing). 2023;3(5):20220173. 10.1002/exp.20220173. 37933278 10.1002/EXP.20220173 PMC10582614

[22] O’Brien WJ, Gupta K, Itani KMF. Association of postoperative infection with risk of long-term infection and mortality. JAMA Surg. 2020;155(1):61–8. 10.1001/jamasurg.2019.4539. 31693076 10.1001/jamasurg.2019.4539 PMC6865239

[23] Ward RA, Fawell S, Floc’h N, Flemington V, McKerrecher D, Smith PD. Challenges and opportunities in cancer drug resistance. Chem Rev. 2021;121(6):3297–351. 10.1021/acs.chemrev.0c00383. 32692162 10.1021/acs.chemrev.0c00383

[24] Yang Q, Xu J, Gu J, Shi H, Zhang J, Zhang J, et al. Extracellular vesicles in cancer drug resistance: Roles, mechanisms, and implications. Adv Sci (Weinh). 2022;9(34):e2201609. 10.1002/advs.202201609. 36253096 10.1002/advs.202201609 PMC9731723

[25] Li R, Liu K, Huang X, Li D, Ding J, Liu B, et al. Bioactive materials promote wound healing through modulation of cell behaviors. Adv Sci (Weinh). 2022;9(10):e2105152. 10.1002/advs.202105152. 35138042 10.1002/advs.202105152 PMC8981489

[26] Muthu S, Korpershoek JV, Novais EJ, Tawy GF, Hollander AP, Martin I. Failure of cartilage regeneration: Emerging hypotheses and related therapeutic strategies. Nat Rev Rheumatol. 2023;19(7):403–16. 10.1038/s41584-023-00979-5. 37296196 10.1038/s41584-023-00979-5

[27] Liu L, McClements DJ, Liu X, Liu F. Overcoming biopotency barriers: Advanced oral delivery strategies for enhancing the efficacy of bioactive food ingredients. Adv Sci (Weinh). 2024;11(44):e2401172. 10.1002/advs.202401172. 39361948 10.1002/advs.202401172 PMC11600209

[28] Zhang Y, Zhang Y, Ding R, Zhang K, Guo H, Lin Y. Self-assembled nanocarrier delivery systems for bioactive compounds. Small. 2024;20(26):e2310838. 10.1002/smll.202310838. 38214694 10.1002/smll.202310838

[29] Hoffman AS. Hydrogels for biomedical applications. Adv Drug Deliv Rev. 2002;54(1):3–12. 10.1016/s0169-409x(01)00239-3. 11755703 10.1016/s0169-409x(01)00239-3

[30] Peppas NA, Hilt JZ, Khademhosseini A, Langer R. Hydrogels in biology and medicine: From molecular principles to bionanotechnology. Adv Mater. 2006;18(11):1345–60. 10.1002/adma.200501612.

[31] Caliari SR, Burdick JA. A practical guide to hydrogels for cell culture. Nat Methods. 2016;13(5):405–14. 10.1038/nmeth.3839. 27123816 10.1038/nmeth.3839 PMC5800304

[32] Burdick JA, Prestwich GD. Hyaluronic acid hydrogels for biomedical applications. Adv Mater. 2011;23(12):H41-56. 10.1002/adma.201003963. 21394792 10.1002/adma.201003963 PMC3730855

[33] Van Vlierberghe S, Dubruel P, Schacht E. Biopolymer-based hydrogels as scaffolds for tissue engineering applications: A review. Biomacromol. 2011;12(5):1387–408. 10.1021/bm200083n. 10.1021/bm200083n 21388145

[34] DeForest CA, Anseth KS. Cytocompatible click-based hydrogels with dynamically tunable properties through orthogonal photoconjugation and photocleavage reactions. Nat Chem. 2011;3(12):925–31. 10.1038/nchem.1174. 22109271 10.1038/nchem.1174 PMC3229165

[35] Lutolf MP, Hubbell JA. Synthetic biomaterials as instructive extracellular microenvironments for morphogenesis in tissue engineering. Nat Biotechnol. 2005;23(1):47–55. 10.1038/nbt1055. 15637621 10.1038/nbt1055

[36] Webber MJ, Langer R. Drug delivery by supramolecular design. Chem Soc Rev. 2017;46(21):6600–20. 10.1039/c7cs00391a. 28828455 10.1039/c7cs00391a

[37] Appel EA, del Barrio J, Loh XJ, Scherman OA. Supramolecular polymeric hydrogels. Chem Soc Rev. 2012;41(18):6195–214. 10.1039/c2cs35264h. 22890548 10.1039/c2cs35264h

[38] Hersel U, Dahmen C, Kessler H. Rgd modified polymers: Biomaterials for stimulated cell adhesion and beyond. Biomaterials. 2003;24(24):4385–415. 10.1016/s0142-9612(03)00343-0. 12922151 10.1016/s0142-9612(03)00343-0

[39] Geiger B, Spatz JP, Bershadsky AD. Environmental sensing through focal adhesions. Nat Rev Mol Cell Biol. 2009;10(1):21–33. 10.1038/nrm2593. 19197329 10.1038/nrm2593

[40] Gong JP. Why are double network hydrogels so tough? Soft Matter. 2010;6(12):2583–90. 10.1039/B924290B.

[41] Sun JY, Zhao X, Illeperuma WR, Chaudhuri O, Oh KH, Mooney DJ, et al. Highly stretchable and tough hydrogels. Nature. 2012;489(7414):133–6. 10.1038/nature11409. 22955625 10.1038/nature11409 PMC3642868

[42] Haraguchi K, Takehisa T. Nanocomposite hydrogels: A unique organic–inorganic network structure with extraordinary mechanical, optical, and swelling/de-swelling properties. Adv Mater. 2002;14(16):1120–4. 10.1002/1521-4095(20020816)14:16<1120::AID-ADMA1120>3.0.CO;2-9.

[43] Sletten EM, Bertozzi CR. Bioorthogonal chemistry: Fishing for selectivity in a sea of functionality. Angew Chem Int Ed Engl. 2009;48(38):6974–98. 10.1002/anie.200900942. 19714693 10.1002/anie.200900942 PMC2864149

[44] Perera MM, Ayres N. Dynamic covalent bonds in self-healing, shape memory, and controllable stiffness hydrogels. Polym Chem. 2020;11(8):1410–23. 10.1039/c9py01694e.

[45] Whitesides GM. The origins and the future of microfluidics. Nature. 2006;442(7101):368–73. 10.1038/nature05058. 16871203 10.1038/nature05058

[46] Li D, Xia Y. Electrospinning of nanofibers: reinventing the wheel? Adv Mater. 2004;16(14):1151–70. 10.1002/adma.200400719.

[47] Xie J, MacEwan MR, Schwartz AG, Xia Y. Electrospun nanofibers for neural tissue engineering. Nanoscale. 2010;2(1):35–44. 10.1039/b9nr00243j. 20648362 10.1039/b9nr00243j

[48] Murphy SV, Atala A. 3D bioprinting of tissues and organs. Nat Biotechnol. 2014;32(8):773–85. 10.1038/nbt.2958. 25093879 10.1038/nbt.2958

[49] Hinton TJ, Jallerat Q, Palchesko RN, Park JH, Grodzicki MS, Shue H-J, et al. Three-dimensional printing of complex biological structures by freeform reversible embedding of suspended hydrogels. Sci Adv. 2015;1(9):e1500758. 10.1126/sciadv.1500758. 26601312 10.1126/sciadv.1500758 PMC4646826

[50] Kloxin AM, Kasko AM, Salinas CN, Anseth KS. Photodegradable hydrogels for dynamic tuning of physical and chemical properties. Science. 2009;324(5923):59–63. 10.1126/science.1169494. 19342581 10.1126/science.1169494 PMC2756032

[51] Patterson J, Hubbell JA. Enhanced proteolytic degradation of molecularly engineered peg hydrogels in response to mmp-1 and mmp-2. Biomaterials. 2010;31(30):7836–45. 10.1016/j.biomaterials.2010.06.061. 20667588 10.1016/j.biomaterials.2010.06.061

[52] Tibbitt MW, Anseth KS. Hydrogels as extracellular matrix mimics for 3d cell culture. Biotechnol Bioeng. 2009;103(4):655–63. 10.1002/bit.22361. 19472329 10.1002/bit.22361 PMC2997742

[53] Soliman BG, Nguyen AK, Gooding JJ, Kilian KA. Advancing synthetic hydrogels through nature-inspired materials chemistry. Adv Mater. 2024;36(42):e2404235. 10.1002/adma.202404235. 38896849 10.1002/adma.202404235 PMC11486603

[54] Su YC, Chen G, Lai YJ, Song GZ, Wu TL, Yeh YC. Tailoring the dynamic nanocomposite hydrogels through surface-functionalized nanomaterials and interfacial crosslinking chemistry toward multifunctional biomedical and engineering applications. Chem Soc Rev. 2026;55(2):819–68. 10.1039/d5cs00975h. 41257301 10.1039/d5cs00975h

[55] Stuart MA, Huck WT, Genzer J, Müller M, Ober C, Stamm M, et al. Emerging applications of stimuli-responsive polymer materials. Nat Mater. 2010;9(2):101–13. 10.1038/nmat2614. 20094081 10.1038/nmat2614

[56] Wei M, Gao Y, Li X, Serpe MJ. Stimuli-responsive polymers and their applications. Polym Chem. 2017;8(1):127–43. 10.1039/c6py01585a.

[57] Qiu Y, Park K. Environment-sensitive hydrogels for drug delivery. Adv Drug Deliv Rev. 2001;53(3):321–39. 10.1016/s0169-409x(01)00203-4. 11744175 10.1016/s0169-409x(01)00203-4

[58] Gu Z, Biswas A, Zhao M, Tang Y. Tailoring nanocarriers for intracellular protein delivery. Chem Soc Rev. 2011;40(7):3638–55. 10.1039/c0cs00227e. 21566806 10.1039/c0cs00227e

[59] Nathan C, Cunningham-Bussel A. Beyond oxidative stress: An immunologist’s guide to reactive oxygen species. Nat Rev Immunol. 2013;13(5):349–61. 10.1038/nri3423. 23618831 10.1038/nri3423 PMC4250048

[60] Hanahan D, Weinberg RA. Hallmarks of cancer: The next generation. Cell. 2011;144(5):646–74. 10.1016/j.cell.2011.02.013. 21376230 10.1016/j.cell.2011.02.013

[61] Tibbitt MW, Dahlman JE, Langer R. Emerging frontiers in drug delivery. J Am Chem Soc. 2016;138(3):704–17. 10.1021/jacs.5b09974. 26741786 10.1021/jacs.5b09974

[62] Schmaljohann D. Thermo- and ph-responsive polymers in drug delivery. Adv Drug Deliv Rev. 2006;58(15):1655–70. 10.1016/j.addr.2006.09.020. 17125884 10.1016/j.addr.2006.09.020

[63] Zhang J, Peppas NA. Synthesis and characterization of ph- and temperature-sensitive poly(methacrylic acid)/poly(n-isopropylacrylamide) interpenetrating polymeric networks. Macromolecules. 2000;33(1):102–7. 10.1021/ma991398q.

[64] Klouda L, Mikos AG. Thermoresponsive hydrogels in biomedical applications. Eur J Pharm Biopharm. 2008;68(1):34–45. 10.1016/j.ejpb.2007.02.025. 17881200 10.1016/j.ejpb.2007.02.025 PMC3163097

[65] Binauld S, Stenzel MH. Acid-degradable polymers for drug delivery: A decade of innovation. Chem Commun. 2013;49(21):2082–102. 10.1039/C2CC36589H. 10.1039/c2cc36589h 23320254

[66] Cheng R, Feng F, Meng F, Deng C, Feijen J, Zhong Z. Glutathione-responsive nano-vehicles as a promising platform for targeted intracellular drug and gene delivery. J Control Release. 2011;152(1):2–12. 10.1016/j.jconrel.2011.01.030. 21295087 10.1016/j.jconrel.2011.01.030

[67] Meng F, Hennink WE, Zhong Z. Reduction-sensitive polymers and bioconjugates for biomedical applications. Biomaterials. 2009;30(12):2180–98. 10.1016/j.biomaterials.2009.01.026. 19200596 10.1016/j.biomaterials.2009.01.026

[68] Timko BP, Dvir T, Kohane DS. Remotely triggerable drug delivery systems. Adv Mater. 2010;22(44):4925–43. 10.1002/adma.201002072. 20818618 10.1002/adma.201002072

[69] Optogenetics DK. Nat Methods. 2011;8(1):26–9. 10.1038/nmeth.f.324. 21191368 10.1038/nmeth.f.324 PMC6814250

[70] Hu Q, Katti PS, Gu Z. Enzyme-responsive nanomaterials for controlled drug delivery. Nanoscale. 2014;6(21):12273–86. 10.1039/C4NR04249B. 25251024 10.1039/c4nr04249b PMC4425417

[71] Ulijn RV. Enzyme-responsive materials: A new class of smart biomaterials. J Mater Chem. 2006;16(23):2217–25. 10.1039/B601776M.

[72] Chen G, Roy I, Yang C, Prasad PN. Nanochemistry and nanomedicine for nanoparticle-based diagnostics and therapy. Chem Rev. 2016;116(5):2826–85. 10.1021/acs.chemrev.5b00148. 26799741 10.1021/acs.chemrev.5b00148

[73] Yang Y, Urban MW. Self-healing polymeric materials. Chem Soc Rev. 2013;42(17):7446–67. 10.1039/c3cs60109a. 23864042 10.1039/c3cs60109a

[74] Chaudhuri O, Gu L, Klumpers D, Darnell M, Bencherif SA, Weaver JC, et al. Hydrogels with tunable stress relaxation regulate stem cell fate and activity. Nat Mater. 2016;15(3):326–34. 10.1038/nmat4489. 26618884 10.1038/nmat4489 PMC4767627

[75] Handorf AM, Zhou Y, Halanski MA, Li W-J. Tissue stiffness dictates development, homeostasis, and disease progression. Organogenesis. 2015;11(1):1–15. 10.1080/15476278.2015.1019687. 25915734 10.1080/15476278.2015.1019687 PMC4594591

[76] Chen Z, Wang Z, Gu Z. Bioinspired and biomimetic nanomedicines. Acc Chem Res. 2019;52(5):1255–64. 10.1021/acs.accounts.9b00079. 30977635 10.1021/acs.accounts.9b00079 PMC7293770

[77] Jokerst JV, Gambhir SS. Molecular imaging with theranostic nanoparticles. Acc Chem Res. 2011;44(10):1050–60. 10.1021/ar200106e. 21919457 10.1021/ar200106e PMC3196845

[78] Xiong Y, Li M, Qing G. Biomolecule-responsive polymers and their bio-applications. Interdiscip Mater. 2024;3(6):865–96. 10.1002/idm2.12210.

[79] Yang Z-H, Yin J, Xin L, Li Y, Huang Y, Yuan R, et al. Research advancement of DNA-based intelligent hydrogels: Manufacture, characteristics, application of disease diagnosis and treatment. Chin Chem Lett. 2024;35(10):109558. 10.1016/j.cclet.2024.109558.

[80] Materón EM, Miyazaki CM, Carr O, Joshi N, Picciani PHS, Dalmaschio CJ, et al. Magnetic nanoparticles in biomedical applications: A review. Appl Surf Sci Adv. 2021;6:100163. 10.1016/j.apsadv.2021.100163.

[81] Yu C, Qiu Y, Yao F, Wang C, Li J. Chemically programmed hydrogels for spatiotemporal modulation of the cardiac pathological microenvironment. Adv Mater. 2024;36(32):e2404264. 10.1002/adma.202404264. 38830198 10.1002/adma.202404264

[82] Zhang P, Qin Q, Cao X, Xiang H, Feng D, Wusiman D, et al. Hydrogel microspheres for bone regeneration through regulation of the regenerative microenvironment. Biomater Transl. 2024;5(3):205–35. 10.12336/biomatertransl.2024.03.002. 10.12336/biomatertransl.2024.03.002 PMC11681181 39734698

[83] Anderson JM, Rodriguez A, Chang DT. Foreign body reaction to biomaterials. Semin Immunol. 2008;20(2):86–100. 10.1016/j.smim.2007.11.004. 18162407 10.1016/j.smim.2007.11.004 PMC2327202

[84] Williams DF. On the mechanisms of biocompatibility. Biomaterials. 2008;29(20):2941–53. 10.1016/j.biomaterials.2008.04.023. 18440630 10.1016/j.biomaterials.2008.04.023

[85] Jia B, Zhao X, Wan X, Wu Z, Wu Y, Huang H. Biofunctional and interface-engineered hydrogels for advanced tissue engineering. Adv Healthc Mater. 2025;14(28):e2502146. 10.1002/adhm.202502146. 40817676 10.1002/adhm.202502146

[86] Tang H, Li Y, Liao S, Liu H, Qiao Y, Zhou J. Multifunctional conductive hydrogel interface for bioelectronic recording and stimulation. Adv Healthc Mater. 2024;13(22):e2400562. 10.1002/adhm.202400562. 38773929 10.1002/adhm.202400562

[87] Bryers JD, Giachelli CM, Ratner BD. Engineering biomaterials to integrate and heal: The biocompatibility paradigm shifts. Biotechnol Bioeng. 2012;109(8):1898–911. 10.1002/bit.24559. 22592568 10.1002/bit.24559 PMC3490630

[88] Veiseh O, Doloff JC, Ma M, Vegas AJ, Tam HH, Bader AR, et al. Size- and shape-dependent foreign body immune response to materials implanted in rodents and non-human primates. Nat Mater. 2015;14(6):643–51. 10.1038/nmat4290. 25985456 10.1038/nmat4290 PMC4477281

[89] Franz S, Rammelt S, Scharnweber D, Simon JC. Immune responses to implants - a review of the implications for the design of immunomodulatory biomaterials. Biomaterials. 2011;32(28):6692–709. 10.1016/j.biomaterials.2011.05.078. 21715002 10.1016/j.biomaterials.2011.05.078

[90] Hynes RO. The extracellular matrix: Not just pretty fibrils. Science. 2009;326(5957):1216–9. 10.1126/science.1176009. 19965464 10.1126/science.1176009 PMC3536535

[91] Lee KY, Mooney DJ. Alginate: Properties and biomedical applications. Prog Polym Sci. 2012;37(1):106–26. 10.1016/j.progpolymsci.2011.06.003. 22125349 10.1016/j.progpolymsci.2011.06.003 PMC3223967

[92] Benoit DS, Schwartz MP, Durney AR, Anseth KS. Small functional groups for controlled differentiation of hydrogel-encapsulated human mesenchymal stem cells. Nat Mater. 2008;7(10):816–23. 10.1038/nmat2269. 18724374 10.1038/nmat2269 PMC2929915

[93] Joyce JA, Pollard JW. Microenvironmental regulation of metastasis. Nat Rev Cancer. 2009;9(4):239–52. 10.1038/nrc2618. 19279573 10.1038/nrc2618 PMC3251309

[94] Mantovani A, Allavena P, Sica A, Balkwill F. Cancer-related inflammation. Nature. 2008;454(7203):436–44. 10.1038/nature07205. 18650914 10.1038/nature07205

[95] Rosales AM, Anseth KS. The design of reversible hydrogels to capture extracellular matrix dynamics. Nat Rev Mater. 2016;1. 10.1038/natrevmats.2015.12. 10.1038/natrevmats.2015.12 PMC5714327 29214058

[96] Tibbitt MW, Anseth KS. Dynamic microenvironments: the fourth dimension. Sci Transl Med. 2012;4(160):160ps24. 10.1126/scitranslmed.3004804. 23152326 10.1126/scitranslmed.3004804

[97] Meng W, Yang H, Zhang W, Li X, Sun J, Hu T, et al. Ros-triggered self-assembling peptide hydrogel combining bacterial entrapment and intracellular penetration for salmonella typhimurium-induced enteritis therapy. Chem Eng J. 2025;524:169392. 10.1016/j.cej.2025.169392.

[98] Zhou Q, Zhuang Y, Deng X, Jiang W, Wang X, Yuan C, et al. Hydrogel-based ros-regulating strategy: Reprogramming the oxidative stress imbalance in advanced diabetic wound repair. Adv Mater. 2026;38(3):e12719. 10.1002/adma.202512719. 41030212 10.1002/adma.202512719

[99] Du L, Lin C, Hu H, Zhao Y, Liao J, Al-Smadi F, et al. Recent advances and challenges in hydrogel-based delivery of immunomodulatory strategies for diabetic wound healing. Theranostics. 2026;16(1):516–44. 10.7150/thno.117949. 41328337 10.7150/thno.117949 PMC12665143

[100] Kalukula Y, Ciccone G, Mohammed D, Procès A, Versaevel M, Deridoux A, et al. Unlocking the therapeutic potential of cellular mechanobiology. Sci Adv. 2025;11(44):eaea6817. 10.1126/sciadv.aea6817. 41171907 10.1126/sciadv.aea6817 PMC12577691

[101] Chen X, Feng Y, Zhang P, Ni Z, Xue Y, Liu J. Hydrogel fibers-based biointerfacing. Adv Mater. 2025;37(4):e2413476. 10.1002/adma.202413476. 39578344 10.1002/adma.202413476

[102] Wu J, Yun Z, Song W, Yu T, Xue W, Liu Q, et al. Highly oriented hydrogels for tissue regeneration: Design strategies, cellular mechanisms, and biomedical applications. Theranostics. 2024;14(5):1982–2035. 10.7150/thno.89493. 38505623 10.7150/thno.89493 PMC10945336

[103] Feng S, Chen K, Wang S. Practical guide to the design of granular hydrogels for customizing complex cellular microenvironments. Adv Healthc Mater. 2025;14(27):e01947. 10.1002/adhm.202501947. 40734307 10.1002/adhm.202501947 PMC12538541

[104] Zhang R, Tan SF, Wang Y, Wu J, Zhang C. From macrophage polarization to clinical translation: Immunomodulatory hydrogels for infection-associated bone regeneration. Front Cell Dev Biol. 2025;13:1684357. 10.3389/fcell.2025.1684357. 41070348 10.3389/fcell.2025.1684357 PMC12504304

[105] Mohammadzadeh V, Atapour-Mashhad H, Shahvali S, Salehi B, Shaban M, Shirzad M, et al. Hydrogels as advanced drug delivery platforms for cancer immunotherapy: Promising innovations and future outlook. J Nanobiotechnology. 2025;23(1):545. 10.1186/s12951-025-03613-6. 40717098 10.1186/s12951-025-03613-6 PMC12302882

[106] Xiang G, Yin B, Shiroud Heidari B, Youssef G, Gosecka M, Gosecki M, et al. Programmable hydrogels: Frontiers in dynamic closed-loop systems, biomimetic synergy, and clinical translation. Adv Sci (Weinh). 2025;12(47):e12037. 10.1002/advs.202512037. 41235760 10.1002/advs.202512037 PMC12713069

[107] Tumbleston JR, Shirvanyants D, Ermoshkin N, Janusziewicz R, Johnson AR, Kelly D, et al. Additive manufacturing. Continuous liquid interface production of 3d objects. Science. 2015;347(6228):1349–52. 10.1126/science.aaa2397. 10.1126/science.aaa2397 25780246

[108] Grigoryan B, Paulsen SJ, Corbett DC, Sazer DW, Fortin CL, Zaita AJ, et al. Multivascular networks and functional intravascular topologies within biocompatible hydrogels. Science. 2019;364(6439):458–64. 10.1126/science.aav9750. 31048486 10.1126/science.aav9750 PMC7769170

[109] Ovsianikov A, Deiwick A, Van Vlierberghe S, Dubruel P, Möller L, Dräger G, et al. Laser fabrication of three-dimensional cad scaffolds from photosensitive gelatin for applications in tissue engineering. Biomacromol. 2011;12(4):851–8. 10.1021/bm1015305. 10.1021/bm1015305 21366287

[110] Kolesky DB, Homan KA, Skylar-Scott MA, Lewis JA. Three-dimensional bioprinting of thick vascularized tissues. Proc Natl Acad Sci U S A. 2016;113(12):3179–84. 10.1073/pnas.1521342113. 26951646 10.1073/pnas.1521342113 PMC4812707

[111] Dendukuri D, Pregibon DC, Collins J, Hatton TA, Doyle PS. Continuous-flow lithography for high-throughput microparticle synthesis. Nat Mater. 2006;5(5):365–9. 10.1038/nmat1617. 16604080 10.1038/nmat1617

[112] Seiffert S, Weitz DA. Microfluidic fabrication of smart microgels from macromolecular precursors. Polymer. 2010;51(25):5883–9. 10.1016/j.polymer.2010.10.034.

[113] Griffin DR, Weaver WM, Scumpia PO, Di Carlo D, Segura T. Accelerated wound healing by injectable microporous gel scaffolds assembled from annealed building blocks. Nat Mater. 2015;14(7):737–44. 10.1038/nmat4294. 26030305 10.1038/nmat4294 PMC4615579

[114] Bhatia SN, Ingber DE. Microfluidic organs-on-chips. Nat Biotechnol. 2014;32(8):760–72. 10.1038/nbt.2989. 25093883 10.1038/nbt.2989

[115] Huh D, Matthews BD, Mammoto A, Montoya-Zavala M, Hsin HY, Ingber DE. Reconstituting organ-level lung functions on a chip. Science. 2010;328(5986):1662–8. 10.1126/science.1188302. 20576885 10.1126/science.1188302 PMC8335790

[116] Zhang B, Radisic M. Organ-on-a-chip devices advance to market. Lab Chip. 2017;17(14):2395–420. 10.1039/c6lc01554a. 28617487 10.1039/c6lc01554a

[117] Shi Y, Li D, Yi B, Tang H, Xu T, Zhang Y. Physiological cyclic stretching potentiates the cell-cell junctions in vascular endothelial layer formed on aligned fiber substrate. Biomater Adv. 2024;157:213751. 10.1016/j.bioadv.2023.213751. 38219418 10.1016/j.bioadv.2023.213751

[118] Tabassum A, Rajasekaran SP, Kumar KK, Deng M, Hu Z. Influence of electrospinning parameters on polycaprolactone fiber alignment for the differentiation of embryonic stem cells into neuronal lineage - a systematic study. Biomater Adv. 2026;179:214485. 10.1016/j.bioadv.2025.214485. 40987149 10.1016/j.bioadv.2025.214485

[119] Dalby MJ, Gadegaard N, Tare R, Andar A, Riehle MO, Herzyk P, et al. The control of human mesenchymal cell differentiation using nanoscale symmetry and disorder. Nat Mater. 2007;6(12):997–1003. 10.1038/nmat2013. 17891143 10.1038/nmat2013

[120] Wang X-w, Zheng H-y, Wang J, Yu H-m, Tang Q, Fu G-s, et al. Impact of micro-scale regular topography on cell and tissue behaviors. Sci China Mater. 2024;67(7):2090–102. 10.1007/s40843-024-2917-7.

[121] Wang Y, Wang T, Tang R, Wang D, Zhang X, Nagaumi H, et al. Multiscale interface engineering in biohybrid composites for biomedical applications. Mater Today Bio. 2025;35:102382. 10.1016/j.mtbio.2025.102382. 41080738 10.1016/j.mtbio.2025.102382 PMC12513274

[122] DeForest CA, Tirrell DA. A photoreversible protein-patterning approach for guiding stem cell fate in three-dimensional gels. Nat Mater. 2015;14(5):523–31. 10.1038/nmat4219. 25707020 10.1038/nmat4219

[123] Miao S, Castro N, Nowicki M, Xia L, Cui H, Zhou X, et al. 4d printing of polymeric materials for tissue and organ regeneration. Mater Today (Kidlington). 2017;20(10):577–91. 10.1016/j.mattod.2017.06.005. 29403328 10.1016/j.mattod.2017.06.005 PMC5796676

[124] Butler KT, Davies DW, Cartwright H, Isayev O, Walsh A. Machine learning for molecular and materials science. Nature. 2018;559(7715):547–55. 10.1038/s41586-018-0337-2. 30046072 10.1038/s41586-018-0337-2

[125] Truby RL, Lewis JA. Printing soft matter in three dimensions. Nature. 2016;540(7633):371–8. 10.1038/nature21003. 27974748 10.1038/nature21003

[126] Cianchetti M, Ranzani T, Gerboni G, Nanayakkara T, Althoefer K, Dasgupta P. et al. Soft robotics technologies to address shortcomings in today's minimally invasive surgery: The stiff-flop approach. Soft Robotics. 2014;1(2014)(2):122–31. 10.1089/soro.2014.0001.

[127] Liu X, Inda ME, Lai Y, Lu TK, Zhao X. Engineered living hydrogels. Adv Mater. 2022;34(26):e2201326. 10.1002/adma.202201326. 35243704 10.1002/adma.202201326 PMC9250645

[128] Langer R. Drug delivery and targeting. Nature. 1998;392(6679 Suppl):5–10. 9579855

[129] Mitragotri S, Burke PA, Langer R. Overcoming the challenges in administering biopharmaceuticals: formulation and delivery strategies. Nat Rev Drug Discov. 2014;13(9):655–72. 10.1038/nrd4363. 25103255 10.1038/nrd4363 PMC4455970

[130] Webber MJ, Appel EA, Meijer EW, Langer R. Supramolecular biomaterials. Nat Mater. 2016;15(1):13–26. 10.1038/nmat4474. 26681596 10.1038/nmat4474

[131] Peer D, Karp JM, Hong S, Farokhzad OC, Margalit R, Langer R. Nanocarriers as an emerging platform for cancer therapy. Nat Nanotechnol. 2007;2(12):751–60. 10.1038/nnano.2007.387. 18654426 10.1038/nnano.2007.387

[132] Dong S, Chapman SL, Pluen A, Richardson SM, Miller AF, Saiani A. Effect of peptide-polymer host-guest electrostatic interactions on self-assembling peptide hydrogels structural and mechanical properties and polymer diffusivity. Biomacromol. 2024;25(6):3628–41. 10.1021/acs.biomac.4c00232. 10.1021/acs.biomac.4c00232 PMC11170954 38771115

[133] Wang Q, Cheng Q, Yao G, Wang Z, Zhu L, Zeng Z, et al. A cationic hydrogel with anti-IL-17A-specific nanobodies for rheumatoid arthritis treatment via inhibition of inflammatory activities of neutrophils. Nano Today. 2024;59:102507. 10.1016/j.nantod.2024.102507.

[134] Chang Z, Ran X, Chu Y, Li B, Fan Z, Li G, et al. Dynamic-covalent hybrid hydrogels with cartilaginous immune microenvironment temporally regulating meniscus regeneration. Bioact Mater. 2025;50:14–29. 10.1016/j.bioactmat.2025.03.026. 40242503 10.1016/j.bioactmat.2025.03.026 PMC11998115

[135] Wang T, Huang C, Fang Z, Bahatibieke A, Fan D, Wang X, et al. A dual dynamically cross-linked hydrogel promotes rheumatoid arthritis repair through ros initiative regulation and microenvironment modulation-independent triptolide release. Mater Today Bio. 2024;26:101042. 10.1016/j.mtbio.2024.101042. 38660473 10.1016/j.mtbio.2024.101042 PMC11040138

[136] Kloxin AM, Kloxin CJ, Bowman CN, Anseth KS. Mechanical properties of cellularly responsive hydrogels and their experimental determination. Adv Mater. 2010;22(31):3484–94. 10.1002/adma.200904179. 20473984 10.1002/adma.200904179 PMC3890982

[137] Yufei M, Ting H, Qinxuan Y, Jing W, Bicong F, Yuanbo J, et al. Viscoelastic cell microenvironment: Hydrogel-based strategy for recapitulating dynamic ecm mechanics. Adv Funct Mater. 2021. 10.1002/adfm.202100848.

[138] Zhang K, Feng Q, Fang Z, Gu L, Bian L. Structurally dynamic hydrogels for biomedical applications: Pursuing a fine balance between macroscopic stability and microscopic dynamics. Chem Rev. 2021;121(18):11149–93. 10.1021/acs.chemrev.1c00071. 34189903 10.1021/acs.chemrev.1c00071

[139] Ding X, Zang M, Zhang Y, Chen Y, Du J, Yan A, et al. A bioresponsive diselenide-functionalized hydrogel with cascade catalytic activities for enhanced local starvation- and hypoxia-activated melanoma therapy. Acta Biomater. 2023;167:182–94. 10.1016/j.actbio.2023.06.017. 37339693 10.1016/j.actbio.2023.06.017

[140] Han Y, Liu C, Xu H, Cao Y. Engineering reversible hydrogels for 3d cell culture and release using diselenide catalyzed fast disulfide formation. Chin J Chem. 2022;40(13):1578–84. 10.1002/cjoc.202200099.

[141] Hu Q, Sun W, Wang C, Gu Z. Recent advances of cocktail chemotherapy by combination drug delivery systems. Adv Drug Deliv Rev. 2016;98:19–34. 10.1016/j.addr.2015.10.022. 26546751 10.1016/j.addr.2015.10.022 PMC4998845

[142] Mohanty S, Roy S. Bioactive hydrogels inspired by laminin: An emerging biomaterial for tissue engineering applications. Macromol Biosci. 2024;24(11):e2400207. 10.1002/mabi.202400207. 39172212 10.1002/mabi.202400207

[143] Rijns L, Wijnakker J, Veenbrink VA, Bellan R, Craenmehr FWB, Hendrikse SIS, et al. Controlling intestinal organoid polarity using synthetic dynamic hydrogels decorated with laminin-derived ikvav peptides. Adv Healthc Mater. 2026;15(8):e02079. 10.1002/adhm.202502079. 40904064 10.1002/adhm.202502079 PMC12927536

[144] Aurand ER, Lampe KJ, Bjugstad KB. Defining and designing polymers and hydrogels for neural tissue engineering. Neurosci Res. 2012;72(3):199–213. 10.1016/j.neures.2011.12.005. 22192467 10.1016/j.neures.2011.12.005 PMC3408056

[145] Lutolf MP, Gilbert PM, Blau HM. Designing materials to direct stem-cell fate. Nature. 2009;462(7272):433–41. 10.1038/nature08602. 19940913 10.1038/nature08602 PMC2908011

[146] Mooney DJ, Vandenburgh H. Cell delivery mechanisms for tissue repair. Cell Stem Cell. 2008;2(3):205–13. 10.1016/j.stem.2008.02.005. 18371446 10.1016/j.stem.2008.02.005

[147] Pakulska MM, Ballios BG, Shoichet MS. Injectable hydrogels for central nervous system therapy. Biomed Mater. 2012;7(2):024101. 10.1088/1748-6041/7/2/024101. 22456684 10.1088/1748-6041/7/2/024101

[148] Yang QQ, Jin J, Sun J, Zhang L, Tang JB, Zhou YL. Simultaneous targeting of tgf-β1/pd-l1 via a hydrogel-nanoparticle system to remodel the ecm and immune microenvironment for limiting adhesion formation. Acta Biomater. 2024;190:447–62. 10.1016/j.actbio.2024.10.043. 39481625 10.1016/j.actbio.2024.10.043

[149] Zhao Y, Song S, Zhang J, Ren X, Zhao Y. Hitchhiking delivery systems: Therapeutic hydrogels as advanced tools for immunomodulatory applications. Chem Rev. 2025;125(17):8089–122. 10.1021/acs.chemrev.4c00923. 40854180 10.1021/acs.chemrev.4c00923

[150] Groll J, Burdick JA, Cho DW, Derby B, Gelinsky M, Heilshorn SC, et al. A definition of bioinks and their distinction from biomaterial inks. Biofabrication. 2018;11(1):013001. 10.1088/1758-5090/aaec52. 30468151 10.1088/1758-5090/aaec52

[151] Lancaster MA, Knoblich JA. Organogenesis in a dish: modeling development and disease using organoid technologies. Science. 2014;345(6194):1247125. 10.1126/science.1247125. 25035496 10.1126/science.1247125

[152] Hao R, Niu X, Jiang X, Liu K, Ma X, Chen C. Transglutaminase-triggered dual gradients of mechanical and biochemical cues self-assembling peptide hydrogel for guiding MC3T3-E1 cell behaviors. Int J Biol Macromol. 2025;285:138281. 10.1016/j.ijbiomac.2024.138281. 39631574 10.1016/j.ijbiomac.2024.138281

[153] Niu R, Xin Q, Xu E, Yao S, Chen M, Liu D. Nanostarch-stimulated cell adhesion in 3d bioprinted hydrogel scaffolds for cell cultured meat. ACS Appl Mater Interfaces. 2024;16(18):23015–26. 10.1021/acsami.4c03585. 38680043 10.1021/acsami.4c03585

[154] Engler AJ, Sen S, Sweeney HL, Discher DE. Matrix elasticity directs stem cell lineage specification. Cell. 2006;126(4):677–89. 10.1016/j.cell.2006.06.044. 16923388 10.1016/j.cell.2006.06.044

[155] Burdick JA, Murphy WL. Moving from static to dynamic complexity in hydrogel design. Nat Commun. 2012;3:1269. 10.1038/ncomms2271. 23232399 10.1038/ncomms2271

[156] Amitrano A, Yuan Q, Agarwal B, Sen A, Dance Y W, Zuo Y, et al. Extracellular fluid viscosity regulates human mesenchymal stem cell lineage and function. Sci Adv. 2025;11(1):eadr5023. 10.1126/sciadv.adr5023. 10.1126/sciadv.adr5023 PMC11691697 39742493

[157] Walker M, Pringle EW, Ciccone G, Oliver-Cervelló L, Tassieri M, Gourdon D, et al. Mind the viscous modulus: The mechanotransductive response to the viscous nature of isoelastic matrices regulates stem cell chondrogenesis. Adv Healthc Mater. 2024;13(9):e2302571. 10.1002/adhm.202302571. 38014647 10.1002/adhm.202302571 PMC11481034

[158] Yang C, Tibbitt MW, Basta L, Anseth KS. Mechanical memory and dosing influence stem cell fate. Nat Mater. 2014;13(6):645–52. 10.1038/nmat3889. 24633344 10.1038/nmat3889 PMC4031270

[159] Rane AA, Christman KL. Biomaterials for the treatment of myocardial infarction: A 5-year update. J Am Coll Cardiol. 2011;58(25):2615–29. 10.1016/j.jacc.2011.11.001. 22152947 10.1016/j.jacc.2011.11.001

[160] Wells RG. The role of matrix stiffness in regulating cell behavior. Hepatology. 2008;47(4):1394–400. 10.1002/hep.22193. 18307210 10.1002/hep.22193

[161] Gjorevski N, Sachs N, Manfrin A, Giger S, Bragina ME, Ordóñez-Morán P, et al. Designer matrices for intestinal stem cell and organoid culture. Nature. 2016;539(7630):560–4. 10.1038/nature20168. 27851739 10.1038/nature20168

[162] Kim DH, Ghaffari R, Lu N, Rogers JA. Flexible and stretchable electronics for biointegrated devices. Annu Rev Biomed Eng. 2012;14:113–28. 10.1146/annurev-bioeng-071811-150018. 22524391 10.1146/annurev-bioeng-071811-150018

[163] Rogers JA, Someya T, Huang Y. Materials and mechanics for stretchable electronics. Science. 2010;327(5973):1603–7. 10.1126/science.1182383. 20339064 10.1126/science.1182383

[164] Trung TQ, Lee NE. Flexible and stretchable physical sensor integrated platforms for wearable human-activity monitoringand personal healthcare. Adv Mater. 2016;28(22):4338–72. 10.1002/adma.201504244. 26840387 10.1002/adma.201504244

[165] Yuk H, Lu B, Zhao X. Hydrogel bioelectronics. Chem Soc Rev. 2019;48(6):1642–67. 10.1039/c8cs00595h. 30474663 10.1039/c8cs00595h

[166] Song C-W, Jang W, Hong J, Lim SY, Moon D, Roh HY, et al. Label-free multiplexed protein quantification in clinical samples using encodable hydrogel barcode and low-aspect-ratio micropore. Sens Actuators B Chem. 2025;432:137496. 10.1016/j.snb.2025.137496.

[167] Yang G, Qiu Y, Pang B, Guo W, Liu S, Zheng Q, et al. A reusable hydrogel biosensor array with electrically responsive hydrogel interfaces for noninvasive locating of perforating arteries. Sci Adv. 2025;11(26):eadw6166. 10.1126/sciadv.adw6166. 40561023 10.1126/sciadv.adw6166 PMC12190022

[168] Keplinger C, Sun JY, Foo CC, Rothemund P, Whitesides GM, Suo Z. Stretchable, transparent, ionic conductors. Science. 2013;341(6149):984–7. 10.1126/science.1240228. 23990555 10.1126/science.1240228

[169] Yuk H, Zhang T, Lin S, Parada GA, Zhao X. Tough bonding of hydrogels to diverse non-porous surfaces. Nat Mater. 2016;15(2):190–6. 10.1038/nmat4463. 26552058 10.1038/nmat4463 PMC4762474

[170] Lu Y, Aimetti AA, Langer R, Gu Z. Bioresponsive materials Nat Rev Mater. 2016;2(1):16075. 10.1038/natrevmats.2016.75.

[171] Chenani H, Saeidi M, Rastkhiz MA, Bolghanabadi N, Aghaii AH, Orouji M, et al. Challenges and advances of hydrogel-based wearable electrochemical biosensors for real-time monitoring of biofluids: From lab to market. A review Anal Chem. 2024;96(20):8160–83. 10.1021/acs.analchem.3c03942. 38377558 10.1021/acs.analchem.3c03942

[172] Huang L, Zhou Y, Hu X, Yang Z. Emerging combination of hydrogel and electrochemical biosensors. Small. 2025;21(5):e2409711. 10.1002/smll.202409711. 39679847 10.1002/smll.202409711

[173] Lacour SP, Courtine G, Guck J. Materials and technologies for soft implantable neuroprostheses. Nat Rev Mater. 2016;1(10):16063. 10.1038/natrevmats.2016.63.

[174] Nam JW, Moon C-H, Kim D-H, Kim MH, Park WH. Dynamically crosslinked poly(vinyl alcohol)/borax strain sensors for organ motion monitoring. Chem Eng J. 2024;498:155748. 10.1016/j.cej.2024.155748.

[175] Yu A, Zhu M, Chen C, Li Y, Cui H, Liu S, et al. Implantable flexible sensors for health monitoring. Adv Healthc Mater. 2024;13(2):e2302460. 10.1002/adhm.202302460. 37816513 10.1002/adhm.202302460

[176] Li C, Wu PM, Lee S, Gorton A, Schulz MJ, Ahn CH. Flexible dome and bump shape piezoelectric tactile sensors using pvdf-trfe copolymer. J Microelectromech Syst. 2008;17(2):334–41. 10.1109/JMEMS.2007.911375.

[177] Trung TQ, Ramasundaram S, Hwang BU, Lee NE. An all-elastomeric transparent and stretchable temperature sensor for body-attachable wearable electronics. Adv Mater. 2016;28(3):502–9. 10.1002/adma.201504441. 26607674 10.1002/adma.201504441

[178] Wang X, Gu Y, Xiong Z, Cui Z, Zhang T. Silk-molded flexible, ultrasensitive, and highly stable electronic skin for monitoring human physiological signals. Adv Mater. 2014;26(9):1336–42. 10.1002/adma.201304248. 24347340 10.1002/adma.201304248

[179] Baker BM, Chen CS. Deconstructing the third dimension: how 3d culture microenvironments alter cellular cues. J Cell Sci. 2012;125(Pt 13):3015–24. 10.1242/jcs.079509. 22797912 10.1242/jcs.079509 PMC3434846

[180] Kapałczyńska M, Kolenda T, Przybyła W, Zajączkowska M, Teresiak A, Filas V, et al. 2d and 3d cell cultures - a comparison of different types of cancer cell cultures. Arch Med Sci. 2018;14(4):910–9. 10.5114/aoms.2016.63743. 30002710 10.5114/aoms.2016.63743 PMC6040128

[181] Küstner MJ, Marx C, Graben PB, Rodrigues S, Hampl J, Weise F, et al. Biotechnical multiscale engineering of scaffolds for stem cell and organoid research. Small. 2026;22(12):e04070. 10.1002/smll.202504070. 41166590 10.1002/smll.202504070 PMC12934388

[182] Link R, Weißenbruch K, Tanaka M, Bastmeyer M, Schwarz US. Cell shape and forces in elastic and structured environments: from single cells to organoids. Adv Funct Mater. 2024;34:2302145. 10.1002/adfm.202302145.

[183] Guo Z, Dong Z, Gao J, Zhang H, Zhang B, Li MH, et al. Unlocking auxetic behavior in recyclable thermosetting foams enabled by dynamic disulfide cross-linking strategy. Small. 2024;20(50):e2406876. 10.1002/smll.202406876. 39308248 10.1002/smll.202406876

[184] Jia Y, Qian J, Hao S, Zhang S, Wei F, Zheng H, et al. New prospects arising from dynamically crosslinked polymers: Reprogramming their properties. Adv Mater. 2024;36(21):e2313164. 10.1002/adma.202313164. 38577834 10.1002/adma.202313164

[185] Quadrato G, Nguyen T, Macosko EZ, Sherwood JL, Min Yang S, Berger DR, et al. Cell diversity and network dynamics in photosensitive human brain organoids. Nature. 2017;545(7652):48–53. 10.1038/nature22047. 28445462 10.1038/nature22047 PMC5659341

[186] Chaudhuri O, Cooper-White J, Janmey PA, Mooney DJ, Shenoy VB. Effects of extracellular matrix viscoelasticity on cellular behaviour. Nature. 2020;584(7822):535–46. 10.1038/s41586-020-2612-2. 32848221 10.1038/s41586-020-2612-2 PMC7676152

[187] Nerger BA, Kashyap K, Deveney BT, Lou J, Hanan BF, Liu Q, et al. Tuning porosity of macroporous hydrogels enables rapid rates of stress relaxation and promotes cell expansion and migration. Proc Natl Acad Sci U S A. 2024;121(45):e2410806121. 10.1073/pnas.2410806121. 39467139 10.1073/pnas.2410806121 PMC11551365

[188] Warmflash A, Sorre B, Etoc F, Siggia ED, Brivanlou AH. A method to recapitulate early embryonic spatial patterning in human embryonic stem cells. Nat Methods. 2014;11(8):847–54. 10.1038/nmeth.3016. 24973948 10.1038/nmeth.3016 PMC4341966

[189] Li B, Fu Q, Lu Y, Chen C, Zhao Y, Zhao Y, et al. 3d hydrogel platform with macromolecular actuators for precisely controlled mechanical forces on cancer cell migration. Nat Commun. 2025;16(1):4831. 10.1038/s41467-025-60062-3. 40413192 10.1038/s41467-025-60062-3 PMC12103621

[190] Vernerey FJ, Lalitha Sridhar S, Muralidharan A, Bryant SJ. Mechanics of 3d cell-hydrogel interactions: Experiments, models, and mechanisms. Chem Rev. 2021;121(18):11085–148. 10.1021/acs.chemrev.1c00046. 34473466 10.1021/acs.chemrev.1c00046

[191] Mulero-Russe A, García AJ. Engineered synthetic matrices for human intestinal organoid culture and therapeutic delivery. Adv Mater. 2024;36(9):e2307678. 10.1002/adma.202307678. 37987171 10.1002/adma.202307678 PMC10922691

[192] Wang Y, Cao Z, Li B, Huang Y, Wang G, Chen Q. Hydrogel design for intestinal organoids: Principles governing translational regenerative medicine. Biomater Sci. 2025;13(21):5948–70. 10.1039/d5bm00926j. 40982248 10.1039/d5bm00926j

[193] Kadoshima T, Sakaguchi H, Nakano T, Soen M, Ando S, Eiraku M, et al. Self-organization of axial polarity, inside-out layer pattern, and species-specific progenitor dynamics in human es cell-derived neocortex. Proc Natl Acad Sci U S A. 2013;110(50):20284–9. 10.1073/pnas.1315710110. 24277810 10.1073/pnas.1315710110 PMC3864329

[194] Huch M, Knoblich JA, Lutolf MP, Martinez-Arias A. The hope and the hype of organoid research. Development. 2017;144(6):938–41. 10.1242/dev.150201. 28292837 10.1242/dev.150201

[195] Murphy WL, McDevitt TC, Engler AJ. Materials as stem cell regulators. Nat Mater. 2014;13(6):547–57. 10.1038/nmat3937. 24845994 10.1038/nmat3937 PMC4163547

[196] Homan KA, Gupta N, Kroll KT, Kolesky DB, Skylar-Scott M, Miyoshi T, et al. Flow-enhanced vascularization and maturation of kidney organoids in vitro. Nat Methods. 2019;16(3):255–62. 10.1038/s41592-019-0325-y. 30742039 10.1038/s41592-019-0325-y PMC6488032

[197] Clevers H. Modeling development and disease with organoids. Cell. 2016;165(7):1586–97. 10.1016/j.cell.2016.05.082. 27315476 10.1016/j.cell.2016.05.082

[198] Takebe T, Wells JM. Organoids by design. Science. 2019;364(6444):956–9. 10.1126/science.aaw7567. 31171692 10.1126/science.aaw7567 PMC8212787

[199] Dąbrowska AK, Spano F, Derler S, Adlhart C, Spencer ND, Rossi RM. The relationship between skin function, barrier properties, and body-dependent factors. Skin Res Technol. 2018;24(2):165–74. 10.1111/srt.12424. 29057509 10.1111/srt.12424

[200] Martin P. Wound healing–aiming for perfect skin regeneration. Science. 1997;276(5309):75–81. 10.1126/science.276.5309.75. 9082989 10.1126/science.276.5309.75

[201] Gurtner GC, Werner S, Barrandon Y, Longaker MT. Wound repair and regeneration. Nature. 2008;453(7193):314–21. 10.1038/nature07039. 18480812 10.1038/nature07039

[202] Li Z, Guan S, Duan X, Wang L, Zhang Z, Wu J. Pro-angiogenic functionalized hyaluronic acid-based hydrogel encapsulating metformin coordinates in situ immune-inflammatory modulation for accelerating diabetic wound healing. Int J Biol Macromol. 2025;318(Pt 2):144944. 10.1016/j.ijbiomac.2025.144944. 40473174 10.1016/j.ijbiomac.2025.144944

[203] Rivera AE, Spencer JM. Clinical aspects of full-thickness wound healing. Clin Dermatol. 2007;25(1):39–48. 10.1016/j.clindermatol.2006.10.001. 17276200 10.1016/j.clindermatol.2006.10.001

[204] Robson MC. Wound infection. A failure of wound healing caused by an imbalance of bacteria. Surg Clin North Am. 1997;77(3):637–50. 10.1016/s0039-6109(05)70572-7. 9194884 10.1016/s0039-6109(05)70572-7

[205] Du J, Xian C, Liang X, Fan S, Wang L, Wu J. Advanced responsive hydrogels for diabetic wound healing: Design principles, controlled drug delivery, therapeutic strategies, and application prospects. MedComm - Biomater appl. 2025;4(3):e70019. 10.1002/mba2.70019.

[206] Simões D, Miguel SP, Ribeiro MP, Coutinho P, Mendonça AG, Correia IJ. Recent advances on antimicrobial wound dressing: A review. Eur J Pharm Biopharm. 2018;127:130–41. 10.1016/j.ejpb.2018.02.022. 29462687 10.1016/j.ejpb.2018.02.022

[207] Brumberg V, Astrelina T, Malivanova T, Samoilov A. Modern wound dressings: hydrogel dressings. Biomedicines. 2021. 10.3390/biomedicines9091235. 34572421 10.3390/biomedicines9091235 PMC8472341

[208] Hu H, Xu FJ. Rational design and latest advances of polysaccharide-based hydrogels for wound healing. Biomater Sci. 2020;8(8):2084–101. 10.1039/d0bm00055h. 32118241 10.1039/d0bm00055h

[209] Ahmed EM. Hydrogel: Preparation, characterization, and applications: A review. J Adv Res. 2015;6(2):105–21. 10.1016/j.jare.2013.07.006. 25750745 10.1016/j.jare.2013.07.006 PMC4348459

[210] Ribeiro MP, Espiga A, Silva D, Baptista P, Henriques J, Ferreira C, et al. Development of a new chitosan hydrogel for wound dressing. Wound Repair Regen. 2009;17(6):817–24. 10.1111/j.1524-475X.2009.00538.x. 19903303 10.1111/j.1524-475X.2009.00538.x

[211] Koehler J, Brandl FP, Goepferich AM. Hydrogel wound dressings for bioactive treatment of acute and chronic wounds. Eur Polym J. 2018;100:1–11. 10.1016/j.eurpolymj.2017.12.046.

[212] Xiang J, Shen L, Hong Y. Status and future scope of hydrogels in wound healing: Synthesis, materials and evaluation. Eur Polym J. 2020;130:109609. 10.1016/j.eurpolymj.2020.109609.

[213] Roy S, Fan Z, Ullah Z, Madni M, Shyamal S, Roy J, et al. Ultrasound-powered piezoelectric hydrogel enables dual piezodynamic-chemodynamic therapy and immunomodulation against bacteria-infected burn wounds. Nano Energy. 2025;146:111535. 10.1016/j.nanoen.2025.111535.

[214] Shi H, Zhang W, Zang Y, Guo X, Jiang Z, Sun Y, et al. Copper-manganese bimetallic oxide-encapsulated hydrogels with multienzyme activities accelerate mrsa-infected wound healing by disrupting bacterial protein expression. Acta Biomater. 2025. 10.1016/j.actbio.2025.09.017. 40972744 10.1016/j.actbio.2025.09.017

[215] Ye Y, Liu Y, Ma S, Li X, Wang W, Chen X, et al. Multifunctional DNA hydrogels with light-triggered gas-therapy and controlled g-exos release for infected wound healing. Bioact Mater. 2025;52:422–37. 10.1016/j.bioactmat.2025.06.004. 40585387 10.1016/j.bioactmat.2025.06.004 PMC12206045

[216] Ma H, Luo Y, Wang Y, Hao Y, Li J, Gao X, et al. Artificial multienzyme nanoflower composite hydrogel for efficiently promoting mrsa-infected diabetic wound healing via glucose-activated no releasing and microenvironment regulation. Bioact Mater. 2025;49:531–48. 10.1016/j.bioactmat.2025.03.014. 40212777 10.1016/j.bioactmat.2025.03.014 PMC11982317

[217] Zhang H, Liang Q, Ji Y, Chen Q, Jiang W, Zhang D, et al. Facile fabrication of antioxidative and antibacterial hydrogel films to accelerate infected diabetic wound healing. Bioact Mater. 2025;53:386–403. 10.1016/j.bioactmat.2025.07.026. 40735354 10.1016/j.bioactmat.2025.07.026 PMC12305319

[218] Duan X, Li J, Gao R, Zhou Y, Chen R, Shang Y, et al. Antimicrobial hydrogel loaded with broccoli exosomes promotes anti-scarring healing of mrsa-infected wounds. Mater Today Bio. 2025;35:102276. 10.1016/j.mtbio.2025.102276. 40978160 10.1016/j.mtbio.2025.102276 PMC12446615

[219] Guo J, Wei Y, Guo Z, Wang T, Ma Y, Li H, et al. Triboelectro-responsive DNA hydrogels for accelerated healing of bacterium-infected diabetic wounds. Nano Energy. 2025;142:111217. 10.1016/j.nanoen.2025.111217.

[220] Xie X, Lei H, Fan D. Antibacterial hydrogel with ph-responsive microcarriers of slow-release vegf for bacterial infected wounds repair. J Mater Sci Technol. 2023;144:198–212. 10.1016/j.jmst.2022.09.062.

[221] Ye R, Zhu Z, Gu T, Cao D, Jiang K, Dai Q, et al. Neutrophil extracellular traps-inspired DNA hydrogel for wound hemostatic adjuvant. Nat Commun. 2024;15(1):5557. 10.1038/s41467-024-49933-3. 38956415 10.1038/s41467-024-49933-3 PMC11219873

[222] Geng Y, Gao Y, Qi D, Wang Z, Zou Z, Zhang Z, et al. A hydrogel tissue adhesive incorporating basic fibroblast growth factor-loaded liposomes accelerates cutaneous wound healing by enhancing cell proliferation, collagen synthesis and angiogenesis. Acta Biomater. 2025;205:242–59. 10.1016/j.actbio.2025.08.035. 40848946 10.1016/j.actbio.2025.08.035

[223] Guo Y, Zhao X, Wang Y, Zhou J, Lu T, Luo G, et al. Injectable hemostatic foam hydrogel for traumatic intra-abdominal hemorrhage. Mater Today Bio. 2025;35:102364. 10.1016/j.mtbio.2025.102364. 41127547 10.1016/j.mtbio.2025.102364 PMC12537577

[224] Tan L, Li M, Chen H, Zhang Y, Liu Y, Chen M, et al. Dynamic hydrogel with environment-adaptive autonomous wound-compressing ability enables rapid hemostasis and inflammation amelioration for hemorrhagic wound healing. Nano Today. 2023;52:101962. 10.1016/j.nantod.2023.101962.

[225] Zhu S, Zhao B, Li M, Wang H, Zhu J, Li Q, et al. Microenvironment responsive nanocomposite hydrogel with NIR photothermal therapy, vascularization and anti-inflammation for diabetic infected wound healing. Bioact Mater. 2023;26:306–20. 10.1016/j.bioactmat.2023.03.005. 36950149 10.1016/j.bioactmat.2023.03.005 PMC10027510

[226] Gao H, Luo J, Chen X, Huang Y, Mo M, Luo Q, et al. Immunomodulatory copper-based polyphenol nanozyme for diabetic infectious wound healing via nir amplified cuproptosis bacteriostat in synergy with ferroptosis inhibition anti-inflammation. Bioact Mater. 2025;54:759–76. 10.1016/j.bioactmat.2025.08.042. 41268351 10.1016/j.bioactmat.2025.08.042 PMC12628149

[227] Cong R, Deng C, Li P, Tang Y, Hou J, Zhao J, et al. Dimeric copper peptide incorporated hydrogel for promoting diabetic wound healing. Nat Commun. 2025;16(1):5797. 10.1038/s41467-025-61141-1. 40592840 10.1038/s41467-025-61141-1 PMC12215000

[228] Deng H, Wang F, Zhou Y, Lei H, Zhou H, Chen S, et al. Biosynthesis of a dual growth factors (gfs) functionalized silk sericin hydrogel to promote chronic wound healing in diabetic mice. Bioact Mater. 2025;52:511–28. 10.1016/j.bioactmat.2025.06.017. 40599340 10.1016/j.bioactmat.2025.06.017 PMC12212160

[229] Liu X, Sun Y, Wang J, Kang Y, Wang Z, Cao W, et al. A tough, antibacterial and antioxidant hydrogel dressing accelerates wound healing and suppresses hypertrophic scar formation in infected wounds. Bioact Mater. 2024;34:269–81. 10.1016/j.bioactmat.2023.12.019. 38261887 10.1016/j.bioactmat.2023.12.019 PMC10794931

[230] Parsons JB, Mourad A, Conlon BP, Kielian T, Fowler VG Jr. Methicillin-resistant and susceptible *Staphylococcus aureus*: tolerance, immune evasion and treatment. Nat Rev Microbiol. 2026;24(2):127–45. 10.1038/s41579-025-01226-2.40835978 10.1038/s41579-025-01226-2

[231] Sabrin S, Hong SH, Karmokar DK, Habibullah H, Fitridge R, Short RD, et al. Healing wounds with plasma-activated hydrogel therapy. Trends Biotechnol. 2025;43(2):278–89. 10.1016/j.tibtech.2024.07.013. 39209604 10.1016/j.tibtech.2024.07.013

[232] Huang J, Chen L, Yuan Q, Gu Z, Wu J. Tofu-based hybrid hydrogels with antioxidant and low immunogenicity activity for enhanced wound healing. J Biomed Nanotechnol. 2019;15(7):1371–83. 10.1166/jbn.2019.2814. 31196344 10.1166/jbn.2019.2814

[233] Xie L, Zhang X, Wang X, Zhang Z, Nie T, Wu J, et al. Multifunctional gelma hydrogel doped with spermidine-ferrocene polymeric nanoparticles for accelerative diabetic wound healing. Chin Chem Lett. 2025;36(11):110848. 10.1016/j.cclet.2025.110848.

[234] Fernandez Alarcon J, Perez Schmidt P, Panini N, Caruso F, Violatto MB, Sukubo NG, et al. Functional polarization of liver macrophages by glyco gold nanoparticles. Adv Sci (Weinh). 2025;12(16):e2407458. 10.1002/advs.202407458. 39950558 10.1002/advs.202407458 PMC12021048

[235] Zhan J, Zou J, Pang Q, Chen Z, Liu J, Liu S, et al. Mscs-evs harboring oa immune memory reprogram macrophage phenotype via modulation of the mt-nd3/nadh-coq axis for oa treatment. J Nanobiotechnology. 2025;23(1):140. 10.1186/s12951-025-03216-1. 40001168 10.1186/s12951-025-03216-1 PMC11863759

[236] Huang H, Xie Y, Zhong J, Fu Z, Wu P, Chen X, et al. Antimicrobial peptides loaded collagen nanosheets with enhanced antibacterial activity, corneal wound healing and m1 macrophage polarization in bacterial keratitis. COMPOS PART B-ENG. 2024;275:111283. 10.1016/j.compositesb.2024.111283.

[237] Wang Y, Zhou C, Li Z, Li G, Zou Y, Li X, et al. Injectable immunoregulatory hydrogels sequentially drive phenotypic polarization of macrophages for infected wound healing. Bioact Mater. 2024;41:193–206. 10.1016/j.bioactmat.2024.07.015. 39149597 10.1016/j.bioactmat.2024.07.015 PMC11326493

[238] Zhang M, Yuan F, Jia H, Xu Y, Yan L, Zhang T, et al. Rapidly in situ forming antibiotic-free injectable hydrogel wound dressing for eradicating drug-resistant bacterial infections in human skin organoids. Int J Biol Macromol. 2024;282(Pt 6):137542. 10.1016/j.ijbiomac.2024.137542. 39537051 10.1016/j.ijbiomac.2024.137542

[239] Zhao J, Du J, Zheng Y, Fang Y, Huang J, Wu J, et al. Zinc ions coordinated carboxymethyl chitosan hydrogel doped with ellagic acid for accelerative diabetic wound healing. Int J Biol Macromol. 2025;327(Pt 1):147372. 10.1016/j.ijbiomac.2025.147372. 40907919 10.1016/j.ijbiomac.2025.147372

[240] Xiang G, Wang B, Zhang W, Dong Y, Tao J, Zhang A, et al. A Zn-MOF-GOx-based cascade nanoreactor promotes diabetic infected wound healing by NO release and microenvironment regulation. Acta Biomater. 2024;182:245–59. 10.1016/j.actbio.2024.05.015. 38729545 10.1016/j.actbio.2024.05.015

[241] Zhou Y, Dai F, Zhao S, Li Z, Liang H, Wang X, et al. Ph and glucose dual-responsive hydrogels promoted diabetic wound healing by remodeling the wound microenvironment. Adv Healthc Mater. 2025;14(15):e2500810. 10.1002/adhm.202500810. 40237168 10.1002/adhm.202500810

[242] Zhao Z, Han S, Feng W, Zhang Z, Shen S, Huang H, et al. *Xanthium strumarium*/gelatin methacryloyl based hydrogels with anti-inflammatory and antioxidant properties for diabetic wound healing via akt/mtor pathway. Int J Biol Macromol. 2025;300:140186. 10.1016/j.ijbiomac.2025.140186. 39864703 10.1016/j.ijbiomac.2025.140186

[243] Jain A, Kolipaka T, Pandey G, Priyadarshinee A, Puri N, Shinde S, et al. Innovative approaches to diabetic wound healing: focusing on ros and redox signals. Mol Pharm. 2025;22(10):5738–66. 10.1021/acs.molpharmaceut.5c00491. 40932003 10.1021/acs.molpharmaceut.5c00491

[244] Zhang Q, Liu Z, Wu K, Zhang Z, Guo W, Guan X. Biomaterials for regulating the micro-environment of diabetic foot ulcers. Chin Chem Lett. 2026;37(6):111493. 10.1016/j.cclet.2025.111493.

[245] Zhou C, Geng Y, Zhang C, Hsu Y, Ma L, Li M, et al. Research progress of responsive electrospun dressings for precise monitoring and treatment of diabetic wounds. Burns Trauma. 2026;14:tkaf049. 10.1093/burnst/tkaf049. 10.1093/burnst/tkaf049 PMC13019705 41907171

[246] Xiang J, Wang Y, Yang L, Zhang X, Hong Y, Shen L. A novel hydrogel based on *Bletilla striata* polysaccharide for rapid hemostasis: synthesis, characterization and evaluation. Int J Biol Macromol. 2022;196:1–12. 10.1016/j.ijbiomac.2021.11.166. 34843815 10.1016/j.ijbiomac.2021.11.166

[247] Fan X, Xue R, Li N, Yang Q, Yu C, Dong Y, et al. A rapid hemostasis for incompressible wounds by microwave-assisting construction of cellulose sponge with chitosan. Mater Design. 2022;221:111009. 10.1016/j.matdes.2022.111009.

[248] Lai Y, Peng W, Jiang Y, Zhang Y, Zhang Y, Wang K, et al. Sprayable self-gelling powder based multifunctional tissue adhesives for rapid hemostasis and robust tissue adhesion. Chem Eng J. 2025;513:162928. 10.1016/j.cej.2025.162928.

[249] Liu A, Cui S, Song L, Guo X, Huang Z, Wang S, et al. Ultrafast self-gelling, superabsorbent, and adhesive chitosan-based hemostatic powders for rapid hemostasis and wound healing. Carbohydr Polym. 2025;355:123362. 10.1016/j.carbpol.2025.123362. 40037735 10.1016/j.carbpol.2025.123362

[250] Vilahur G, Fuster V. Interplay between platelets and coagulation: From protective haemostasis to pathological arterial thrombosis. Eur Heart J. 2025;46(5):413–23. 10.1093/eurheartj/ehae776. 39673717 10.1093/eurheartj/ehae776

[251] Costantini TW, Kornblith LZ, Pritts T, Coimbra R. The intersection of coagulation activation and inflammation after injury: What you need to know. J Trauma Acute Care Surg. 2024;96(3):347–56. 10.1097/ta.0000000000004190. 37962222 10.1097/TA.0000000000004190 PMC11001294

[252] Fanaee S, Austin W, Filiaggi M, Adibnia V. External bleeding and advanced biomacromolecules for hemostasis. Biomacromol. 2024;25(11):6936–66. 10.1021/acs.biomac.4c00952. 10.1021/acs.biomac.4c00952 39463174

[253] Wang L, Hou W, Zhang Q, Qiao H, Lin M, Shen Z, et al. Hierarchical self-assembly of injectable alginate supramolecular nanofibril hydrogels for hemostasis in vivo. Adv Fiber Mater. 2024;6(2):489–500. 10.1007/s42765-023-00355-8.

[254] Meng X, Liu F, Shi C, Dou Y, Zhang J, Jiang X, et al. Injectable in-situ curable hydrogel for medullary cavity hemostasis. Front Bioeng Biotechnol. 2025;13:1658768. 10.3389/fbioe.2025.1658768. 41064529 10.3389/fbioe.2025.1658768 PMC12500688

[255] Shi J, Wang D, Wang H, Yang X, Gu S, Wang Y, et al. An injectable hemostatic peg-based hydrogel with on-demand dissolution features for emergency care. Acta Biomater. 2022;145:106–21. 10.1016/j.actbio.2022.04.020. 35436591 10.1016/j.actbio.2022.04.020

[256] Koons GL, Diba M, Mikos AG. Materials design for bone-tissue engineering. Nat Rev Mater. 2020;5(8):584–603. 10.1038/s41578-020-0204-2.

[257] Bose S, Sarkar N, Banerjee D. Natural medicine delivery from biomedical devices to treat bone disorders: A review. Acta Biomater. 2021;126:63–91. 10.1016/j.actbio.2021.02.034. 33657451 10.1016/j.actbio.2021.02.034 PMC8247456

[258] Zhang F-X, Chien M-H, Fan Q-r, Jiang D. Advances in bioadhesive hydrogels for musculoskeletal tissue application. Adv Funct Mater. 2024;34(32):2316540. 10.1002/adfm.202316540.

[259] Yang J, Xia P, Meng F, Li X, Xu X. Bio-functional hydrogel microspheres for musculoskeletal regeneration. Adv Funct Mater. 2024;34(33):2400257. 10.1002/adfm.202400257.

[260] Kang S, Shi X, Chen Y, Zhang L, Liu Q, Lin Z, et al. Injectable decellularized wharton’s jelly hydrogel containing cd56(+) umbilical cord mesenchymal stem cell-derived exosomes for meniscus tear healing and cartilage protection. Mater Today Bio. 2024;29:101258. 10.1016/j.mtbio.2024.101258. 39347017 10.1016/j.mtbio.2024.101258 PMC11437876

[261] Sun J, Chan YT, Ho KWK, Zhang L, Bian L, Tuan RS, et al. “Slow walk” mimetic tensile loading maintains human meniscus tissue resident progenitor cells homeostasis in photocrosslinked gelatin hydrogel. Bioact Mater. 2023;25:256–72. 10.1016/j.bioactmat.2023.01.025. 36825224 10.1016/j.bioactmat.2023.01.025 PMC9941420

[262] Li Z, Yan W, Zhao F, Wang H, Cheng J, Duan X, et al. Regional specific tunable meniscus decellularized extracellular matrix (mdecm) reinforced bioink promotes anistropic meniscus regeneration. Chem Eng J. 2023;473:145209. 10.1016/j.cej.2023.145209.

[263] Vinikoor T, Dzidotor GK, Le TT, Liu Y, Kan HM, Barui S, et al. Injectable and biodegradable piezoelectric hydrogel for osteoarthritis treatment. Nat Commun. 2023;14(1):6257. 10.1038/s41467-023-41594-y. 37802985 10.1038/s41467-023-41594-y PMC10558537

[264] Zhao Y, Song S, Wang D, Liu H, Zhang J, Li Z, et al. Nanozyme-reinforced hydrogel as a h(2)o(2)-driven oxygenerator for enhancing prosthetic interface osseointegration in rheumatoid arthritis therapy. Nat Commun. 2022;13(1):6758. 10.1038/s41467-022-34481-5. 36351899 10.1038/s41467-022-34481-5 PMC9646710

[265] Seo BB, Kwon Y, Kim J, Hong KH, Kim SE, Song HR, et al. Injectable polymeric nanoparticle hydrogel system for long-term anti-inflammatory effect to treat osteoarthritis. Bioact Mater. 2022;7:14–25. 10.1016/j.bioactmat.2021.05.028. 34466714 10.1016/j.bioactmat.2021.05.028 PMC8377411

[266] Zhu J, Wei Y, Yang G, Zhang J, Liu J, Zhang X, et al. Peptide-based rigid nanorod-reinforced gelatin methacryloyl hydrogels for osteochondral regeneration and additive manufacturing. Nat Commun. 2025;16(1):7090. 10.1038/s41467-025-62540-0. 40750776 10.1038/s41467-025-62540-0 PMC12317061

[267] Li Q, Yu H, Zhao F, Cao C, Wu T, Fan Y, et al. 3d printing of microenvironment-specific bioinspired and exosome-reinforced hydrogel scaffolds for efficient cartilage and subchondral bone regeneration. Adv Sci (Weinh). 2023;10(26):e2303650. 10.1002/advs.202303650. 37424038 10.1002/advs.202303650 PMC10502685

[268] Shen C, Wang J, Li G, Hao S, Wu Y, Song P, et al. Boosting cartilage repair with silk fibroin-DNA hydrogel-based cartilage organoid precursor. Bioact Mater. 2024;35:429–44. 10.1016/j.bioactmat.2024.02.016. 38390528 10.1016/j.bioactmat.2024.02.016 PMC10881360

[269] Zhang Q, Yang Y, Suo D, Zhao S, Cheung JC, Leung PH, et al. A biomimetic adhesive and robust janus patch with anti-oxidative, anti-inflammatory, and anti-bacterial activities for tendon repair. ACS Nano. 2023;17(17):16798–816. 10.1021/acsnano.3c03556. 37622841 10.1021/acsnano.3c03556

[270] Zhang W, Rao Y, Wong SH, Wu Y, Zhang Y, Yang R, et al. Transcriptome-optimized hydrogel design of a stem cell niche for enhanced tendon regeneration. Adv Mater. 2025;37(2):e2313722. 10.1002/adma.202313722. 39417770 10.1002/adma.202313722 PMC11733723

[271] Timmer KB, Killian ML, Harley BAC. Mesenchymal stem cell activity across a graded scaffold-hydrogel composite biomaterial for tendon-to-bone enthesis repair. Bioact Mater. 2025;53:287–99. 10.1016/j.bioactmat.2025.07.017. 40703334 10.1016/j.bioactmat.2025.07.017 PMC12284061

[272] Zhao Z, Li G, Ruan H, Chen K, Cai Z, Lu G, et al. Capturing magnesium ions via microfluidic hydrogel microspheres for promoting cancellous bone regeneration. ACS Nano. 2021;15(8):13041–54. 10.1021/acsnano.1c02147. 34342981 10.1021/acsnano.1c02147

[273] Li Y, Xiao L, Wei D, Liu S, Zhang Z, Lian R, et al. Injectable biomimetic hydrogel guided functional bone regeneration by adapting material degradation to tissue healing. Adv Funct Mater. 2023;33(19):2213047. 10.1002/adfm.202213047.

[274] Qin L, Yang S, Zhao C, Yang J, Li F, Xu Z, et al. Prospects and challenges for the application of tissue engineering technologies in the treatment of bone infections. Bone Res. 2024;12(1):28. 10.1038/s41413-024-00332-w. 38744863 10.1038/s41413-024-00332-w PMC11094017

[275] Shi X, Xu C, Chen Z, Li M, Yin Z, Wang B, et al. Biomaterial-mediated macrophage polarization remodeling and sequential regulation: A potential strategy in bone infections treatment. Bone Res. 2025;13(1):96. 10.1038/s41413-025-00471-8. 41290567 10.1038/s41413-025-00471-8 PMC12647917

[276] Guo X, Dong X, Zou G, Zhang H, Zeng K, Gao H, et al. Multiscale toughening mechanisms in biomimetic tendon-like hydrogels. Proc Natl Acad Sci U S A. 2025;122(9):e2424124122. 10.1073/pnas.2424124122. 40014567 10.1073/pnas.2424124122 PMC11892624

[277] Xu Z, Chen Y, Cao Y, Xue B. Tough hydrogels with different toughening mechanisms and applications. Int J Mol Sci. 2024. 10.3390/ijms25052675. 38473922 10.3390/ijms25052675 PMC10932079

[278] Li S, Sheng Y, You Y, Wang Y, Zhang Y, Tao J, et al. Study on vegfa mrna delivery via gelma hydrogel-encapsulated extracellular vesicles for enhanced bone regeneration. Mater Today Bio. 2025;34:102144. 10.1016/j.mtbio.2025.102144. 40791800 10.1016/j.mtbio.2025.102144 PMC12337882

[279] Wang L, Wei X, He X, Xiao S, Shi Q, Chen P, et al. Osteoinductive dental pulp stem cell-derived extracellular vesicle-loaded multifunctional hydrogel for bone regeneration. ACS Nano. 2024;18(12):8777–97. 10.1021/acsnano.3c11542. 38488479 10.1021/acsnano.3c11542

[280] Xi Y, Huang C, Qiu B, Chen T, Wang W, Xu G, et al. Multifunctional hydrogel platform encapsulating lotus root-derived extracellular vesicles stimulate osteogenesis-angiogenesis coupling for accelerated bone regeneration. Chem Eng J. 2026;528:171906. 10.1016/j.cej.2025.171906.

[281] Lin Y, Jiang S, Yao Y, Li H, Jin H, Yang G, et al. Posttranslational modification in bone homeostasis and osteoporosis. MedComm. 2020;6(4):e70159. 10.1002/mco2.70159. 10.1002/mco2.70159 PMC11959162 40170748

[282] Liu X, Hu H, Ma J, Wang B. Mineralized cellulose nanofibers reinforced bioactive hydrogel remodels the osteogenic and angiogenic microenvironment for enhancing bone regeneration. Carbohydr Polym. 2025;357:123480. 10.1016/j.carbpol.2025.123480. 40159001 10.1016/j.carbpol.2025.123480

[283] Zhang Z, Hao Z, Xian C, Zhang J, Wu J. Triple functional magnesium ascorbyl phosphate encapsulated hydrogel: A cosmetic ingredient promotes bone repair via anti-oxidation, calcium uptake and blood vessel remodeling. Chem Eng J. 2023;472:145061. 10.1016/j.cej.2023.145061.

[284] Xi Y, Zhang Z, Zhao Z, Qiu B, Wang W, Xu G, et al. Injectable thymosin β4-modified hyaluronic acid hydrogel with exosomes for stem cell homing and neuronic-angiogenic-osteogenic coupled cranial repair. ACS Nano. 2025;19(25):22710–24. 10.1021/acsnano.4c10386. 40528381 10.1021/acsnano.4c10386

[285] Li G, Luo Y, Hu Z, Shi Z, Cao X, Xu R, et al. Recent advances in peptide-functionalized hydrogels for bone tissue engineering. ACS Biomater Sci Eng. 2025;11(4):1970–89. 10.1021/acsbiomaterials.4c02198. 40178194 10.1021/acsbiomaterials.4c02198 PMC12002065

[286] Sophia Fox AJ, Bedi A, Rodeo SA. The basic science of articular cartilage: Structure, composition, and function. Sports Health. 2009;1(6):461–8. 10.1177/1941738109350438. 23015907 10.1177/1941738109350438 PMC3445147

[287] Kwon H, Brown WE, Lee CA, Wang D, Paschos N, Hu JC, et al. Surgical and tissue engineering strategies for articular cartilage and meniscus repair. Nat Rev Rheumatol. 2019;15(9):550–70. 10.1038/s41584-019-0255-1. 31296933 10.1038/s41584-019-0255-1 PMC7192556

[288] Makris EA, Gomoll AH, Malizos KN, Hu JC, Athanasiou KA. Repair and tissue engineering techniques for articular cartilage. Nat Rev Rheumatol. 2015;11(1):21–34. 10.1038/nrrheum.2014.157. 25247412 10.1038/nrrheum.2014.157 PMC4629810

[289] Liu W, Guo NY, Wang JQ, Xu BB. Osteoarthritis: Mechanisms and therapeutic advances. MedComm (2020). 2025;6(8):e70290. 10.1002/mco2.70290. 10.1002/mco2.70290 PMC12314552 40757100

[290] Wang X, He W, Huang H, Han J, Wang R, Li H, et al. Recent advances in hydrogel technology in delivering mesenchymal stem cell for osteoarthritis therapy. Biomolecules. 2024;14(7). 10.3390/biom14070858. 10.3390/biom14070858 PMC11274544 39062572

[291] Mow VC, Guo XE. Mechano-electrochemical properties of articular cartilage: their inhomogeneities and anisotropies. Annu Rev Biomed Eng. 2002;4:175–209. 10.1146/annurev.bioeng.4.110701.120309. 12117756 10.1146/annurev.bioeng.4.110701.120309

[292] Lee HP, Gu L, Mooney DJ, Levenston ME, Chaudhuri O. Mechanical confinement regulates cartilage matrix formation by chondrocytes. Nat Mater. 2017;16(12):1243–51. 10.1038/nmat4993. 28967913 10.1038/nmat4993 PMC5701824

[293] Levett PA, Melchels FP, Schrobback K, Hutmacher DW, Malda J, Klein TJ. A biomimetic extracellular matrix for cartilage tissue engineering centered on photocurable gelatin, hyaluronic acid and chondroitin sulfate. Acta Biomater. 2014;10(1):214–23. 10.1016/j.actbio.2013.10.005. 24140603 10.1016/j.actbio.2013.10.005

[294] Richardson BM, Wilcox DG, Randolph MA, Anseth KS. Hydrazone covalent adaptable networks modulate extracellular matrix deposition for cartilage tissue engineering. Acta Biomater. 2019;83:71–82. 10.1016/j.actbio.2018.11.014. 30419278 10.1016/j.actbio.2018.11.014 PMC6291351

[295] Zhang A, Zhu S, Sun B, Nan C, Cong L, Zhao Z, et al. 3d cell-laden scaffold printed with brain acellular matrix bioink. J Nanobiotechnology. 2025;23(1):564. 10.1186/s12951-025-03644-z. 40796885 10.1186/s12951-025-03644-z PMC12344831

[296] Liu J, Du C, Liu S, Liu J, Luo X, Zhan J, et al. Emerging trends in injectable stimuli-responsive hydrogel microspheres: Design strategies and therapeutic innovations. MedComm - Biomater appl. 2025;4(2):e70017. 10.1002/mba2.70017.

[297] Zhang S, Chuah SJ, Lai RC, Hui JHP, Lim SK, Toh WS. Msc exosomes mediate cartilage repair by enhancing proliferation, attenuating apoptosis and modulating immune reactivity. Biomaterials. 2018;156:16–27. 10.1016/j.biomaterials.2017.11.028. 29182933 10.1016/j.biomaterials.2017.11.028

[298] Schneider MC, Chu S, Randolph MA, Bryant SJ. An in vitro and in vivo comparison of cartilage growth in chondrocyte-laden matrix metalloproteinase-sensitive poly(ethylene glycol) hydrogels with localized transforming growth factor β3. Acta Biomater. 2019;93:97–110. 10.1016/j.actbio.2019.03.046. 30914256 10.1016/j.actbio.2019.03.046 PMC6980342

[299] Gao Y, Wang J, Dai W, Li S, Liu Q, Zhao X, et al. Collagen-based hydrogels induce hyaline cartilage regeneration by immunomodulation and homeostasis maintenance. Acta Biomater. 2024;186:108–24. 10.1016/j.actbio.2024.07.018. 39067644 10.1016/j.actbio.2024.07.018

[300] Pang Q, Chen Z, Li X, Zhan J, Huang W, Lei Y, et al. Cytokine-activated mesenchymal-stem-cell-derived extracellular matrix facilitates cartilage repair by enhancing chondrocyte homeostasis and chondrogenesis of recruited stem cells. Research (Wash D C). 2025;8:0700. 10.34133/research.0700. 10.34133/research.0700 PMC12494090 41049612

[301] Li F, Yang X, Chen Y, Gong M, Li L, Chen A, et al. Bionic gradient scaffolds for osteochondral tissue engineering: Construction strategies, interface optimization, gradient characterization, and controllability research. Biomater Sci. 2026;14(2):305–39. 10.1039/d5bm01230a. 41284309 10.1039/d5bm01230a

[302] Wang Y, Qin X, Feng Y, Zhang T, Wang X, Li J, et al. Dual-gradient silk-based hydrogel for spatially targeted delivery and osteochondral regeneration. Adv Mater. 2025;37(13):e2420394. 10.1002/adma.202420394. 39967369 10.1002/adma.202420394

[303] Daly AC, Cunniffe GM, Sathy BN, Jeon O, Alsberg E, Kelly DJ. 3d bioprinting of developmentally inspired templates for whole bone organ engineering. Adv Healthc Mater. 2016;5(18):2353–62. 10.1002/adhm.201600182. 27281607 10.1002/adhm.201600182

[304] Daly AC, Freeman FE, Gonzalez-Fernandez T, Critchley SE, Nulty J, Kelly DJ. 3d bioprinting for cartilage and osteochondral tissue engineering. Adv Healthc Mater. 2017. 10.1002/adhm.201700298. 28804984 10.1002/adhm.201700298

[305] Ji Z, Ren X, Jin J, Ye X, Yu H, Fang W, et al. Injectable hydrogel encapsulating simmp13 with anti-ros and anti-apoptotic functions for osteoarthritis treatment. J Nanobiotechnology. 2024;22(1):466. 10.1186/s12951-024-02740-w. 39095867 10.1186/s12951-024-02740-w PMC11297633

[306] Li A, Huang J, Chen J, Wu L, Zeng H, Deng Z, et al. Evolving functional hydrogel strategies for cartilage engineering: From fundamentals to functional regeneration. Burns Trauma. 2025;13:tkaf041. 10.1093/burnst/tkaf041. 10.1093/burnst/tkaf041 PMC12450350 40984979

[307] Jin Y, Koh RH, Kim SH, Kim KM, Park GK, Hwang NS. Injectable anti-inflammatory hyaluronic acid hydrogel for osteoarthritic cartilage repair. Mater Sci Eng C Mater Biol Appl. 2020;115:111096. 10.1016/j.msec.2020.111096. 32600700 10.1016/j.msec.2020.111096

[308] Wu P, Tong Z, Luo L, Zhao Y, Chen F, Li Y, et al. Comprehensive strategy of conduit guidance combined with vegf producing schwann cells accelerates peripheral nerve repair. Bioact Mater. 2021;6(10):3515–27. 10.1016/j.bioactmat.2021.03.020. 33842738 10.1016/j.bioactmat.2021.03.020 PMC8008177

[309] Qiu S, Shi Y, Zang H, Sun X, Wang Q, Fu X, et al. Multifunctional injectable microspheres for osteoarthritis therapy via spatiotemporally modulating macrophage polarization and inflammation. NPJ Regen Med. 2024;9(1):22. 10.1038/s41536-024-00368-w. 39289387 10.1038/s41536-024-00368-w PMC11408510

[310] Yu H, Gao R, Liu Y, Fu L, Zhou J, Li L. Stimulus-responsive hydrogels as drug delivery systems for inflammation targeted therapy. Adv Sci (Weinh). 2024;11(1):e2306152. 10.1002/advs.202306152. 37985923 10.1002/advs.202306152 PMC10767459

[311] Yu H, Huang C, Kong X, Ma J, Ren P, Chen J, et al. Nanoarchitectonics of cartilage-targeting hydrogel microspheres with reactive oxygen species responsiveness for the repair of osteoarthritis. ACS Appl Mater Interfaces. 2022;14(36):40711–23. 10.1021/acsami.2c12703. 36063108 10.1021/acsami.2c12703

[312] Han Y, Yang J, Zhao W, Wang H, Sun Y, Chen Y, et al. Biomimetic injectable hydrogel microspheres with enhanced lubrication and controllable drug release for the treatment of osteoarthritis. Bioact Mater. 2021;6(10):3596–607. 10.1016/j.bioactmat.2021.03.022. 33869900 10.1016/j.bioactmat.2021.03.022 PMC8022850

[313] Li G, Liu S, Chen Y, Zhao J, Xu H, Weng J, et al. An injectable liposome-anchored teriparatide incorporated gallic acid-grafted gelatin hydrogel for osteoarthritis treatment. Nat Commun. 2023;14(1):3159. 10.1038/s41467-023-38597-0. 37258510 10.1038/s41467-023-38597-0 PMC10232438

[314] Qin W, Ma Z, Bai G, Qin W, Li L, Hao D, et al. Neurovascularization inhibiting dual responsive hydrogel for alleviating the progression of osteoarthritis. Nat Commun. 2025;16(1):1390. 10.1038/s41467-025-56727-8. 39910066 10.1038/s41467-025-56727-8 PMC11799281

[315] Tao SC, Huang JY, Gao Y, Li ZX, Wei ZY, Dawes H, et al. Small extracellular vesicles in combination with sleep-related circrna3503: A targeted therapeutic agent with injectable thermosensitive hydrogel to prevent osteoarthritis. Bioact Mater. 2021;6(12):4455–69. 10.1016/j.bioactmat.2021.04.031. 34027234 10.1016/j.bioactmat.2021.04.031 PMC8120802

[316] Nourissat G, Berenbaum F, Duprez D. Tendon injury: From biology to tendon repair. Nat Rev Rheumatol. 2015;11(4):223–33. 10.1038/nrrheum.2015.26. 25734975 10.1038/nrrheum.2015.26

[317] Thomopoulos S, Parks WC, Rifkin DB, Derwin KA. Mechanisms of tendon injury and repair. J Orthop Res. 2015;33(6):832–9. 10.1002/jor.22806. 25641114 10.1002/jor.22806 PMC4418182

[318] Snedeker JG, Foolen J. Tendon injury and repair - a perspective on the basic mechanisms of tendon disease and future clinical therapy. Acta Biomater. 2017;63:18–36. 10.1016/j.actbio.2017.08.032. 28867648 10.1016/j.actbio.2017.08.032

[319] Liu Y, Ramanath HS, Wang DA. Tendon tissue engineering using scaffold enhancing strategies. Trends Biotechnol. 2008;26(4):201–9. 10.1016/j.tibtech.2008.01.003. 18295915 10.1016/j.tibtech.2008.01.003

[320] Matsumoto K, Shimizu N. Activation of the phospholipase c signaling pathway in nerve growth factor-treated neurons by carbon nanotubes. Biomaterials. 2013;34(24):5988–94. 10.1016/j.biomaterials.2013.04.038. 23669261 10.1016/j.biomaterials.2013.04.038

[321] Klotz BJ, Gawlitta D, Rosenberg A, Malda J, Melchels FPW. Gelatin-methacryloyl hydrogels: towards biofabrication-based tissue repair. Trends Biotechnol. 2016;34(5):394–407. 10.1016/j.tibtech.2016.01.002. 26867787 10.1016/j.tibtech.2016.01.002 PMC5937681

[322] Yin Z, Guo J, Wu TY, Chen X, Xu LL, Lin SE, et al. Stepwise differentiation of mesenchymal stem cells augments tendon-like tissue formation and defect repair in vivo. Stem Cells Transl Med. 2016;5(8):1106–16. 10.5966/sctm.2015-0215. 27280798 10.5966/sctm.2015-0215 PMC4954446

[323] Lu HH, Thomopoulos S. Functional attachment of soft tissues to bone: Development, healing, and tissue engineering. Annu Rev Biomed Eng. 2013;15:201–26. 10.1146/annurev-bioeng-071910-124656. 23642244 10.1146/annurev-bioeng-071910-124656 PMC3925419

[324] Sung YK, Kim SW. Recent advances in polymeric drug delivery systems. Biomater Res. 2020;24:12. 10.1186/s40824-020-00190-7. 32537239 10.1186/s40824-020-00190-7 PMC7285724

[325] Dimatteo R, Darling NJ, Segura T. In situ forming injectable hydrogels for drug delivery and wound repair. Adv Drug Deliv Rev. 2018;127:167–84. 10.1016/j.addr.2018.03.007. 29567395 10.1016/j.addr.2018.03.007 PMC6003852

[326] Wang X, Liu Y, Jiang Y, Li Q. Tumor-derived exosomes as promising tools for cancer diagnosis and therapy. Front Pharmacol. 2025;16:1596217. 10.3389/fphar.2025.1596217. 40444049 10.3389/fphar.2025.1596217 PMC12119533

[327] Shen W, Pei P, Zhang C, Li J, Han X, Liu T, et al. A polymeric hydrogel to eliminate programmed death-ligand 1 for enhanced tumor radio-immunotherapy. ACS Nano. 2023;17(23):23998–4011. 10.1021/acsnano.3c08875. 37988029 10.1021/acsnano.3c08875

[328] Sun S, Gu W, Wu H, Zhao Q, Qian S, Xiao H, et al. Immunostimulant in situ hydrogel improves synergetic radioimmunotherapy of malignant glioblastoma relapse post-resection. Adv Funct Mater. 2022;32(43):2205038. 10.1002/adfm.202205038.

[329] Shen H, Wang H, Mo J, Zhang J, Xu C, Sun F, et al. Unrestricted molecular motions enable mild photothermy for recurrence-resistant flash antitumor radiotherapy. Bioact Mater. 2024;37:299–312. 10.1016/j.bioactmat.2024.03.024. 38694765 10.1016/j.bioactmat.2024.03.024 PMC11061705

[330] Zhang Y, Feng Z, Liu J, Li H, Su Q, Zhang J, et al. Polarization of tumor-associated macrophages by tlr7/8 conjugated radiosensitive peptide hydrogel for overcoming tumor radioresistance. Bioact Mater. 2022;16:359–71. 10.1016/j.bioactmat.2021.12.033. 35386314 10.1016/j.bioactmat.2021.12.033 PMC8965723

[331] Wang X, Yuan Z, Tao A, Wang P, Xie W, Yang S, et al. Hydrogel-based patient-friendly photodynamic therapy of oral potentially malignant disorders. Biomaterials. 2022;281:121377. 10.1016/j.biomaterials.2022.121377. 35065330 10.1016/j.biomaterials.2022.121377

[332] Lyu M, Zhang T, Bao Z, Li P, Chen M, Quan H, et al. In situ forming aiegen-alginate hydrogel for remodeling tumor microenvironment to boost flash immunoradiotherapy. Biomaterials. 2025;320:123281. 10.1016/j.biomaterials.2025.123281. 40138965 10.1016/j.biomaterials.2025.123281

[333] Abalos RN, Aziz IA, Caverzan M, Lochedino AS, Ibarra LE, Gallastegui A, et al. Poly(3-hexylthiophene) nanoparticles as visible-light photoinitiators and photosensitizers in 3d printable acrylic hydrogels for photodynamic therapies. Mater Horiz. 2025;12(8):2524–34. 10.1039/d4mh01802h. 40052897 10.1039/d4mh01802h

[334] Hu P, Feng N, Zhao S, Shi J, Yang G, Guo W, et al. Microenvironment-responsive injectable thermosensitive hydrogel incorporating nanozymes for synergistic breast cancer therapy and postsurgical adjuvant treatment. Adv Funct Mater. 2025;35(28):2421176. 10.1002/adfm.202421176.

[335] Sheng S, Yu X, Xing G, Jin L, Zhang Y, Zhu D, et al. An apoptotic body-based vehicle with navigation for photothermal-immunotherapy by precise delivery and tumor microenvironment regulation. Adv Funct Mater. 2023;33(5):2212118. 10.1002/adfm.202212118.

[336] Zhang T, Ping W, Suo M, Pan Y, Huang R, Chen J, et al. Stimuli-responsive hydrogels potentiating photothermal therapy against cancer stem cell-induced breast cancer metastasis. ACS Nano. 2024. 10.1021/acsnano.4c04067. 39046933 10.1021/acsnano.4c04067

[337] Zhang Z, Cao Q, Xia Y, Cui C, Qi Y, Zhang Q, et al. Combination of biodegradable hydrogel and antioxidant bioadhesive for treatment of breast cancer recurrence and radiation skin injury. Bioact Mater. 2024;31:408–21. 10.1016/j.bioactmat.2023.08.021. 37692912 10.1016/j.bioactmat.2023.08.021 PMC10482898

[338] Zhang P, Zhang L, Wang J, Zhu L, Li Z, Chen H, et al. An intelligent hypoxia-relieving chitosan-based nanoplatform for enhanced targeted chemo-sonodynamic combination therapy on lung cancer. Carbohydr Polym. 2021;274:118655. 10.1016/j.carbpol.2021.118655. 34702474 10.1016/j.carbpol.2021.118655

[339] Li C, Zhu P, Xiang H, Jin Y, Lu B, Shen Y, et al. 3d-ceus tracking of injectable chemo-sonodynamic therapy-enabled mop-up of residual renal cell carcinoma after thermal ablation. Mater Today Bio. 2023;18:100513. 10.1016/j.mtbio.2022.100513. 36569591 10.1016/j.mtbio.2022.100513 PMC9771734

[340] Dai Z, Li X, Chen Q, Zhu Y, Shi Z, Deng X, et al. Injectable responsive hydrogel delivery platform: Enabling high tissue penetration and sonogenetic-like potentiating anti-tumor immunotherapy. Adv Funct Mater. 2024;34(19):2313723. 10.1002/adfm.202313723.

[341] Zhang H, Wang Y, Jiang M, Wang K, Yan J, Li G, et al. Inherently anti-metastatic peptide hydrogels for sonodynamic-amplified ferroptosis in cancer therapy. Mater Today Bio. 2025;32:101688. 10.1016/j.mtbio.2025.101688. 40206142 10.1016/j.mtbio.2025.101688 PMC11980000

[342] Yan X, Chen Q. Self-oxygenating hydrogel: Regulation of postsurgical tumor recurrence/metastasis and wound healing. MedComm - Oncol. 2024;3(2):e81. 10.1002/mog2.81.

[343] Hu Z, Zhang H, Li Z, Zhao T, Gu Z, Yuan Q, et al. Multifunctional photothermal hydrogels: Design principles, various functions, and promising biological applications. Chin Chem Lett. 2024;35(10):109527. 10.1016/j.cclet.2024.109527.

[344] Cieślak A, Krakos A, Kulbacka J, Detyna J. Overview of research on additive manufacturing of hydrogel-assisted lab-on-chip platforms for cell engineering applications in photodynamic therapy research. Mikrochim Acta. 2024;191(10):608. 10.1007/s00604-024-06683-9. 39292358 10.1007/s00604-024-06683-9 PMC11410904

[345] Zhao H, Wang Z, Yang S, Zhang R, Guo J, Yang D. Energy-storing DNA-based hydrogel remodels tumor microenvironments for laser-free photodynamic immunotherapy. Biomaterials. 2024;309:122620. 10.1016/j.biomaterials.2024.122620. 38788456 10.1016/j.biomaterials.2024.122620

[346] Pettinelli N, Martinez-Rubi Y, Rodríguez-Llamazares S, González-Chavarría I, Jakubek ZJ, Bouza R, et al. Self-healing chitosan/starch hydrogel as carrier of photosensitizers based on polythiophene/boron nitride nanotubes for photodynamic therapy of cancer cells. Biomater Adv. 2025;177:214398. 10.1016/j.bioadv.2025.214398. 40628172 10.1016/j.bioadv.2025.214398

[347] Lv Z, Xia X, Cao P, Han Q, Zhao H, Zhou R, et al. Overcoming the drug retention barrier with photosensitive hydrogel for sustained photodynamic therapy of oral leukoplakia. Chin Chem Lett. 2026;37(5):111282. 10.1016/j.cclet.2025.111282.

[348] Shi Y, Wu Q, Yang T, Lin J, Yu Q, Yao C, et al. Temperature-activated in situ hydrogel augments tumor treatment. Biomaterials. 2026;326:123633. 10.1016/j.biomaterials.2025.123633. 40850309 10.1016/j.biomaterials.2025.123633

[349] Zhang Z, Cui H, Wang X, Liu J, Liu G, Meng X, et al. Oxidized cellulose-filled double thermo/ph-sensitive hydrogel for local chemo-photothermal therapy in breast cancer. Carbohydr Polym. 2024;332:121931. 10.1016/j.carbpol.2024.121931. 38431421 10.1016/j.carbpol.2024.121931

[350] Quail DF, Joyce JA. Microenvironmental regulation of tumor progression and metastasis. Nat Med. 2013;19(11):1423–37. 10.1038/nm.3394. 24202395 10.1038/nm.3394 PMC3954707

[351] Wu J, Chan YT, Lu Y, Wang N, Feng Y. The tumor microenvironment in the postsurgical liver: Mechanisms and potential targets of postoperative recurrence in human hepatocellular carcinoma. Med Res Rev. 2023;43(6):1946–73. 10.1002/med.21967. 37102365 10.1002/med.21967

[352] Xu Y, Yang L, Wang C, Sun W, Zheng Y, Ou B, et al. Ferroptosis boosted oral cancer photodynamic therapy by carrier-free sorafenib-ce6 self-assembly nanoparticles. J Control Release. 2024;366:798–811. 10.1016/j.jconrel.2023.12.056. 38184236 10.1016/j.jconrel.2023.12.056

[353] Zuo J, Yan H, Qin S, Yan Y, Wang S, Yang L, et al. Extracellular vesicles in cancer drug resistance: Mechanistic insights and therapeutic implications. MedComm - Oncol. 2024;3(4):e94. 10.1002/mog2.94.

[354] Zou Q, Chang R, Xing R, Yuan C, Yan X. Injectable self-assembled bola-dipeptide hydrogels for sustained photodynamic prodrug delivery and enhanced tumor therapy. J Control Release. 2020;319:344–51. 10.1016/j.jconrel.2020.01.002. 31917297 10.1016/j.jconrel.2020.01.002

[355] Zhang Y, Li H, Zhao Y, Liu L, Zhou Y, Pan X, et al. Macrocarpal i induces immunogenic cell death and synergizes with immune checkpoint inhibition by targeting tubulin and parp1 in colorectal cancer. Cell Death Discov. 2025;11(1):73. 10.1038/s41420-025-02360-9. 39987121 10.1038/s41420-025-02360-9 PMC11846858

[356] Xiang Y, Zheng J, Zhao X, Zhou L, Yan Q, Ma Y, et al. Sodium butyrate enhances sorafenib-induced ferroptosis and immunogenic cell death by modulating irf2-oasl2-cgas pathway in colorectal cancer. Mater Today Bio. 2025;35:102498. 10.1016/j.mtbio.2025.102498. 41281652 10.1016/j.mtbio.2025.102498 PMC12639863

[357] Zhang W, Kang M, Li X, Pan Y, Li Z, Zhang Y, et al. Fiber optic-mediated type I photodynamic therapy of brain glioblastoma based on an aggregation-induced emission photosensitizer. Adv Mater. 2024;36(47):e2410142. 10.1002/adma.202410142. 39344926 10.1002/adma.202410142

[358] Cressey P, Abd Shukor SB, Thanou M. Sonodynamic therapy: Transforming sound into light for hard-to-treat tumours. Adv Drug Deliv Rev. 2025;226:115696. 10.1016/j.addr.2025.115696. 40947061 10.1016/j.addr.2025.115696

[359] Huang X, He T, Liang X, Xiang Z, Liu C, Zhou S, et al. Advances and applications of nanoparticles in cancer therapy. MedComm-Oncol. 2024;3(1):e67. 10.1002/mog2.67.

[360] Li D, Zhu Y, Yin W, Lin X, Kim G, Liu Z, et al. Small molecule sonosensitizers in cancer therapy: recent advances and clinical prospects. Chem Soc Rev. 2025;54(16):7610–53. 10.1039/d5cs00088b. 40621820 10.1039/d5cs00088b

[361] Chen L, Chen WD, Xu YX, Ren YY, Zheng C, Lin YY, et al. Strategies for enhancing non-small cell lung cancer treatment: integrating Chinese herbal medicines with epidermal growth factor receptor-tyrosine kinase inhibitors therapy. Eur J Pharmacol. 2024;980:176871. 10.1016/j.ejphar.2024.176871. 39117263 10.1016/j.ejphar.2024.176871

[362] Leighl NB, Akamatsu H, Lim SM, Cheng Y, Minchom AR, Marmarelis ME, et al. Subcutaneous versus intravenous amivantamab, both in combination with lazertinib, in refractory epidermal growth factor receptor-mutated non-small cell lung cancer: primary results from the phase III paloma-3 study. J Clin Oncol. 2024;42(30):3593–605. 10.1200/jco.24.01001. 38857463 10.1200/JCO.24.01001 PMC11469630

[363] Huang RS, Chow R, Benour A, Chen D, Boldt G, Wallis CJD, et al. Comparative efficacy and safety of ablative therapies in the management of primary localised renal cell carcinoma: A systematic review and meta-analysis. Lancet Oncol. 2025;26(3):387–98. 10.1016/s1470-2045(24)00731-9. 39922208 10.1016/S1470-2045(24)00731-9

[364] Xuan L, Bai C, Ju Z, Luo J, Guan H, Zhou PK, et al. Radiation-targeted immunotherapy: a new perspective in cancer radiotherapy. Cytokine Growth Factor Rev. 2024;75:1–11. 10.1016/j.cytogfr.2023.11.003. 38061920 10.1016/j.cytogfr.2023.11.003

[365] Suo M, Shen H, Lyu M, Jiang Y, Liao X, Tang W, et al. Biomimetic nano-cancer stem cell scavenger for inhibition of breast cancer recurrence and metastasis after flash-radiotherapy. Small. 2024;20(29):e2400666. 10.1002/smll.202400666. 38368259 10.1002/smll.202400666

[366] Hu JJ, Urbanic JJ, Case LD, Takita C, Wright JL, Brown DR, et al. Association between inflammatory biomarker c-reactive protein and radiotherapy-induced early adverse skin reactions in a multiracial/ethnic breast cancer population. J Clin Oncol. 2018;36(24):2473–82. 10.1200/jco.2017.77.1790. 29989859 10.1200/JCO.2017.77.1790 PMC6097833

[367] Zhang Z, Xia Y, Li X, Zhang Q, Wu Y, Cui C, et al. Arginine-solubilized lipoic acid-induced β-sheets of silk fibroin-strengthened hydrogel for postoperative rehabilitation of breast cancer. Bioact Mater. 2024;40:667–82. 10.1016/j.bioactmat.2024.08.014. 39257958 10.1016/j.bioactmat.2024.08.014 PMC11386050

[368] Klapka N, Müller HW. Collagen matrix in spinal cord injury. J Neurotrauma. 2006;23(3–4):422–35. 10.1089/neu.2006.23.422. 16629627 10.1089/neu.2006.23.422

[369] Goswami AK, Giduturi VK, Yerramilli SN, Chauhan VS, Yadav N. Tissue engineering: Hydrogel scaffolds and mechanical properties as key design parameters. Adv Colloid Interface Sci. 2026;347:103691. 10.1016/j.cis.2025.103691. 41125016 10.1016/j.cis.2025.103691

[370] Hsu RS, Li SJ, Fang JH, Lee IC, Chu LA, Lo YC, et al. Wireless charging-mediated angiogenesis and nerve repair by adaptable microporous hydrogels from conductive building blocks. Nat Commun. 2022;13(1):5172. 10.1038/s41467-022-32912-x. 36056007 10.1038/s41467-022-32912-x PMC9440098

[371] Yang B, Liang C, Chen D, Cheng F, Zhang Y, Wang S, et al. A conductive supramolecular hydrogel creates ideal endogenous niches to promote spinal cord injury repair. Bioact Mater. 2022;15:103–19. 10.1016/j.bioactmat.2021.11.032. 35386356 10.1016/j.bioactmat.2021.11.032 PMC8941182

[372] Liu W, Luo Y, Ning C, Zhang W, Zhang Q, Zou H, et al. Thermo-sensitive electroactive hydrogel combined with electrical stimulation for repair of spinal cord injury. J Nanobiotechnology. 2021;19(1):286. 10.1186/s12951-021-01031-y. 34556136 10.1186/s12951-021-01031-y PMC8461877

[373] Tan Z, Xiao L, Ma J, Shi K, Liu J, Feng F, et al. Integrating hydrogels manipulate ecm deposition after spinal cord injury for specific neural reconnections via neuronal relays. Sci Adv. 2024;10(27):eado9120. 10.1126/sciadv.ado9120. 38959311 10.1126/sciadv.ado9120 PMC11221524

[374] Yao Z, Yuan W, Xu J, Tong W, Mi J, Ho PC, et al. Magnesium-encapsulated injectable hydrogel and 3d-engineered polycaprolactone conduit facilitate peripheral nerve regeneration. Adv Sci (Weinh). 2022;9(21):e2202102. 10.1002/advs.202202102. 35652188 10.1002/advs.202202102 PMC9313484

[375] Gao Y, Wang Y, Zhang J, Zhang M, Dai C, Zhang Y, et al. Advancing neural regeneration via adaptable hydrogels: Enriched with mg(2+) and silk fibroin to facilitate endogenous cell infiltration and macrophage polarization. Bioact Mater. 2024;33:100–13. 10.1016/j.bioactmat.2023.10.026. 38024231 10.1016/j.bioactmat.2023.10.026 PMC10658209

[376] Lim J, Kim S, Oh SJ, Han SM, Moon SY, Kang B, et al. Mirna sensing hydrogels capable of self-signal amplification for early diagnosis of alzheimer’s disease. Biosens Bioelectron. 2022;209:114279. 10.1016/j.bios.2022.114279. 35447599 10.1016/j.bios.2022.114279

[377] Jiang L, Zhang X, Wang S, Zhang J, Chen J, Lu J, et al. Functional monomers equipped microgel system for managing parkinson’s disease by intervening chemokine axis-mediated nerve cell communications. Adv Sci (Weinh). 2025;12(7):e2410070. 10.1002/advs.202410070. 39721010 10.1002/advs.202410070 PMC11831437

[378] Xu J, Dai P, Zhang C, Dong N, Li C, Tang C, et al. Injectable hierarchical bioactive hydrogels with fibroblast growth factor 21/edaravone/caffeic acid asynchronous delivery for treating parkinson’s disease. Adv Sci (Weinh). 2025;12(4):e2412020. 10.1002/advs.202412020. 39630931 10.1002/advs.202412020 PMC11775539

[379] Xu J, Guo C, Zhou G, Chen H, Dai P, Tang C, et al. An antioxidative injectable chitosan hydrogel based on tannic acid modified dialdehyde polyurethane nano-crosslinker targeting ripk1 to regulate neuroinflammation for amelioration of parkinson’s disease. Biomaterials. 2026;325:123614. 10.1016/j.biomaterials.2025.123614. 40815895 10.1016/j.biomaterials.2025.123614

[380] Yan L, Zhao B, Liu X, Li X, Zeng C, Shi H, et al. Aligned nanofibers from polypyrrole/graphene as electrodes for regeneration of optic nerve via electrical stimulation. ACS Appl Mater Interfaces. 2016;8(11):6834–40. 10.1021/acsami.5b12843. 26926578 10.1021/acsami.5b12843

[381] Zhou L, Fan L, Yi X, Zhou Z, Liu C, Fu R, et al. Soft conducting polymer hydrogels cross-linked and doped by tannic acid for spinal cord injury repair. ACS Nano. 2018;12(11):10957–67. 10.1021/acsnano.8b04609. 30285411 10.1021/acsnano.8b04609

[382] Ausó E, Gómez-Vicente V, Esquiva G. Biomarkers for Alzheimer’s disease early diagnosis. J Pers Med. 2020. 10.3390/jpm10030114. 32899797 10.3390/jpm10030114 PMC7563965

[383] Dalal S, Ramirez-Gomez J, Sharma B, Devara D, Kumar S. Micrornas and synapse turnover in alzheimer’s disease. Ageing Res Rev. 2024;99:102377. 10.1016/j.arr.2024.102377. 38871301 10.1016/j.arr.2024.102377

[384] Li YB, Fu Q, Guo M, Du Y, Chen Y, Cheng Y. Micrornas: Pioneering regulators in alzheimer’s disease pathogenesis, diagnosis, and therapy. Transl Psychiatry. 2024;14(1):367. 10.1038/s41398-024-03075-8. 39256358 10.1038/s41398-024-03075-8 PMC11387755

[385] Yalçin M, Grande V, Outeiro TF, Relógio A. Circadian clock dysfunction in parkinson’s disease: Mechanisms, consequences, and therapeutic strategy. NPJ Parkinsons Dis. 2025;11(1):213. 10.1038/s41531-025-01009-9. 40659664 10.1038/s41531-025-01009-9 PMC12259915

[386] Yoo HS, Lee YG, Sohn YH, Yun M, Cha J, Lee PH. Association of relative brain hyperperfusion independent of dopamine depletion with motor dysfunction in patients with parkinson disease. Neurology. 2024;103(12):e210077. 10.1212/wnl.0000000000210077. 39602666 10.1212/WNL.0000000000210077

[387] Toulouse A, Sullivan AM. Progress in parkinson’s disease-where do we stand? Prog Neurobiol. 2008;85(4):376–92. 10.1016/j.pneurobio.2008.05.003. 18582530 10.1016/j.pneurobio.2008.05.003

[388] Badhiwala JH, Wilson JR, Fehlings MG. Global burden of traumatic brain and spinal cord injury. Lancet Neurol. 2019;18(1):24–5. 10.1016/s1474-4422(18)30444-7. 30497967 10.1016/S1474-4422(18)30444-7

[389] Mukherjee N, Adak A, Ghosh S. Recent trends in the development of peptide and protein-based hydrogel therapeutics for the healing of cns injury. Soft Matter. 2020;16(44):10046–64. 10.1039/d0sm00885k. 32724981 10.1039/d0sm00885k

[390] Zhang Q, Li S, Chen H, Yin J, Chen Y, Liu L, et al. Reduction of oxidative stress and excitotoxicity by mesenchymal stem cell biomimetic co-delivery system for cerebral ischemia-reperfusion injury treatment. Small. 2024;20(43):e2401045. 10.1002/smll.202401045. 38948959 10.1002/smll.202401045

[391] Zhang H, Feng Y, Si Y, Lu C, Wang J, Wang S, et al. Shank3 ameliorates neuronal injury after cerebral ischemia/reperfusion via inhibiting oxidative stress and inflammation. Redox Biol. 2024;69:102983. 10.1016/j.redox.2023.102983. 38064762 10.1016/j.redox.2023.102983 PMC10755590

[392] Jullienne A, Hamer M, Haddad E, Morita A, Gifford P, Hartman R, et al. Acute intranasal osteopontin treatment in male rats following tbi increases the number of activated microglia but does not alter lesion characteristics. J Neurosci Res. 2020;98(1):141–54. 10.1002/jnr.24405. 30892744 10.1002/jnr.24405 PMC6754315

[393] Wang Y, Tan L, Yang Y, Duan H, Liu C, Zhao J, et al. Targeting the ros-ferroptosis-inflammation cycle with a nanozyme-functionalized hydrogel for intervertebral disc repair. Nat Commun. 2025;16(1):11253. 10.1038/s41467-025-66116-w. 41266366 10.1038/s41467-025-66116-w PMC12717100

[394] Lindsay SL, McCanney GA, Willison AG, Barnett SC. Multi-target approaches to cns repair: Olfactory mucosa-derived cells and heparan sulfates. Nat Rev Neurol. 2020;16(4):229–40. 10.1038/s41582-020-0311-0. 32099190 10.1038/s41582-020-0311-0

[395] Lee D, Tran HQ, Dudley AT, Yang K, Yan Z, Xie J. Advancing nerve regeneration: Peripheral nerve injury (pni) chip empowering high-speed biomaterial and drug screening. Chem Eng J. 2024;486:150210. 10.1016/j.cej.2024.150210.

[396] Yang S, Chen L, Bai C, Zhao S, Wu H, Dong X, et al. Polymer scaffolds for peripheral nerve injury repair. Prog Mater Sci. 2025;153:101497. 10.1016/j.pmatsci.2025.101497.

[397] Yang S, Zhu J, Lu C, Chai Y, Cao Z, Lu J, et al. Aligned fibrin/functionalized self-assembling peptide interpenetrating nanofiber hydrogel presenting multi-cues promotes peripheral nerve functional recovery. Bioact Mater. 2022;8:529–44. 10.1016/j.bioactmat.2021.05.056. 34541418 10.1016/j.bioactmat.2021.05.056 PMC8435993

[398] Xuan H, Wu S, Jin Y, Wei S, Xiong F, Xue Y, et al. A bioinspired self-healing conductive hydrogel promoting peripheral nerve regeneration. Adv Sci (Weinh). 2023;10(28):e2302519. 10.1002/advs.202302519. 37612810 10.1002/advs.202302519 PMC10558694

[399] Yang M, Wang L, Liu W, Li W, Huang Y, Jin Q, et al. Highly-stable, injectable, conductive hydrogel for chronic neuromodulation. Nat Commun. 2024;15(1):7993. 10.1038/s41467-024-52418-y. 39266583 10.1038/s41467-024-52418-y PMC11393409

[400] Roth GA, Mensah GA, Johnson CO, Addolorato G, Ammirati E, Baddour LM, et al. Global burden of cardiovascular diseases and risk factors, 1990–2019: Update from the gbd 2019 study. J Am Coll Cardiol. 2020;76(25):2982–3021. 10.1016/j.jacc.2020.11.010. 33309175 10.1016/j.jacc.2020.11.010 PMC7755038

[401] Virani SS, Alonso A, Aparicio HJ, Benjamin EJ, Bittencourt MS, Callaway CW, et al. Heart disease and stroke statistics-2021 update: A report from the american heart association. Circulation. 2021;143(8):e254–743. 10.1161/cir.0000000000000950. 33501848 10.1161/CIR.0000000000000950 PMC13036842

[402] Prabhu SD, Frangogiannis NG. The biological basis for cardiac repair after myocardial infarction: From inflammation to fibrosis. Circ Res. 2016;119(1):91–112. 10.1161/circresaha.116.303577. 27340270 10.1161/CIRCRESAHA.116.303577 PMC4922528

[403] Havakuk O, King KS, Grazette L, Yoon AJ, Fong M, Bregman N, et al. Heart failure-induced brain injury. J Am Coll Cardiol. 2017;69(12):1609–16. 10.1016/j.jacc.2017.01.022. 28335844 10.1016/j.jacc.2017.01.022

[404] Libby P. Inflammation in atherosclerosis-no longer a theory. Clin Chem. 2021;67(1):131–42. 10.1093/clinchem/hvaa275. 33393629 10.1093/clinchem/hvaa275

[405] Feiner R, Dvir T. Tissue–electronics interfaces: From implantable devices to engineered tissues. Nat Rev Mater. 2017;3(1):17076. 10.1038/natrevmats.2017.76.

[406] Travers JG, Kamal FA, Robbins J, Yutzey KE, Blaxall BC. Cardiac fibrosis: The fibroblast awakens. Circ Res. 2016;118(6):1021–40. 10.1161/circresaha.115.306565. 26987915 10.1161/CIRCRESAHA.115.306565 PMC4800485

[407] Shen S, Zhang J, Han Y, Pu C, Duan Q, Huang J, et al. A core-shell nanoreinforced ion-conductive implantable hydrogel bioelectronic patch with high sensitivity and bioactivity for real-time synchronous heart monitoring and repairing. Adv Healthc Mater. 2023;12(29):e2301990. 10.1002/adhm.202301990. 37467758 10.1002/adhm.202301990

[408] Yang K, Dong Q, Yao Z, Liang J, Zhao J, Wu L, et al. A wearable dual-modal patch for rapid pre-hospital diagnosis of acute myocardial infarction. ACS Nano. 2025;19(26):23969–81. 10.1021/acsnano.5c05461. 40552765 10.1021/acsnano.5c05461

[409] Zhang Y, Xu Y, Wu C, Chen S, Chen Z, Qin F, et al. Injectable conductive hydrogel patch with spatiotemporally tailored asymmetric adhesion for myocardial infarction repair. Adv Mater. 2026;38(4):e10824. 10.1002/adma.202510824. 41117075 10.1002/adma.202510824

[410] Lee M, Park J, Choe G, Lee S, Kang BG, Jun JH, et al. A conductive and adhesive hydrogel composed of MXene nanoflakes as a paintable cardiac patch for infarcted heart repair. ACS Nano. 2023;17(13):12290–304. 10.1021/acsnano.3c00933. 37339066 10.1021/acsnano.3c00933

[411] Zhang L, Li T, Yu Y, Shi K, Bei Z, Qian Y, et al. An injectable conductive hydrogel restores electrical transmission at myocardial infarct site to preserve cardiac function and enhance repair. Bioact Mater. 2023;20:339–54. 10.1016/j.bioactmat.2022.06.001. 35784639 10.1016/j.bioactmat.2022.06.001 PMC9210214

[412] Li Q, Feng Q, Zhou H, Lin C, Sun X, Ma C, et al. Mechanisms and therapeutic strategies of extracellular vesicles in cardiovascular diseases. MedComm. 2023;4(6):e454. 10.1002/mco2.454. 38124785 10.1002/mco2.454 PMC10732331

[413] Zhang D, He R, Qu Y, He C, Chu B. Application of biomaterials in cardiac tissue engineering: Current status and prospects. MedComm - Biomater appl. 2024;3(4):e103. 10.1002/mba2.103.

[414] Wu J, Zeng F, Huang XP, Chung JC, Konecny F, Weisel RD, et al. Infarct stabilization and cardiac repair with a vegf-conjugated, injectable hydrogel. Biomaterials. 2011;32(2):579–86. 10.1016/j.biomaterials.2010.08.098. 20932570 10.1016/j.biomaterials.2010.08.098

[415] Windecker S, Stortecky S, Stefanini GG, da Costa BR, Rutjes AW, Di Nisio M, et al. Revascularisation versus medical treatment in patients with stable coronary artery disease: Network meta-analysis. BMJ. 2014;348:g3859. 10.1136/bmj.g3859. 24958153 10.1136/bmj.g3859 PMC4066935

[416] Gao H, Liu S, Qin S, Yang J, Yue T, Ye B, et al. Injectable hydrogel-based combination therapy for myocardial infarction: A systematic review and meta-analysis of preclinical trials. BMC Cardiovasc Disord. 2024;24(1):119. 10.1186/s12872-024-03742-0. 38383333 10.1186/s12872-024-03742-0 PMC10882925

[417] Liang J, Lv R, Li M, Chai J, Wang S, Yan W, et al. Hydrogels for the treatment of myocardial infarction: Design and therapeutic strategies. Macromol Biosci. 2024;24(2):e2300302. 10.1002/mabi.202300302. 37815522 10.1002/mabi.202300302

[418] Hasan A, Khattab A, Islam MA, Hweij KA, Zeitouny J, Waters R, et al. Injectable hydrogels for cardiac tissue repair after myocardial infarction. Adv Sci (Weinh). 2015;2(11):1500122. 10.1002/advs.201500122. 27668147 10.1002/advs.201500122 PMC5033116

[419] Wang LL, Liu Y, Chung JJ, Wang T, Gaffey AC, Lu M, et al. Local and sustained mirna delivery from an injectable hydrogel promotes cardiomyocyte proliferation and functional regeneration after ischemic injury. Nat Biomed Eng. 2017;1:983–92. 10.1038/s41551-017-0157-y. 29354322 10.1038/s41551-017-0157-y PMC5773070

[420] Joner M, Finn AV, Farb A, Mont EK, Kolodgie FD, Ladich E, et al. Pathology of drug-eluting stents in humans: Delayed healing and late thrombotic risk. J Am Coll Cardiol. 2006;48(1):193–202. 10.1016/j.jacc.2006.03.042. 16814667 10.1016/j.jacc.2006.03.042

[421] Yang Z, Tu Q, Zhu Y, Luo R, Li X, Xie Y, et al. Mussel-inspired coating of polydopamine directs endothelial and smooth muscle cell fate for re-endothelialization of vascular devices. Adv Healthc Mater. 2012;1(5):548–59. 10.1002/adhm.201200073. 23184789 10.1002/adhm.201200073

[422] Peng P, Ding S, Liang M, Zheng W, Kang Y, Liu W, et al. A self-sacrificing anti-inflammatory coating promotes simultaneous cardiovascular repair and reendothelialization of implanted devices. Bioact Mater. 2025;47:502–12. 10.1016/j.bioactmat.2025.01.037. 40026826 10.1016/j.bioactmat.2025.01.037 PMC11872464

[423] Liang H, Du Y, Liu L, Liu J, Chen Y. A tailored artificial DNase blocks sensor activation and prevents autoimmune and autoinflammatory diseases. Adv Funct Mater. 2023;33(19):2213465. 10.1002/adfm.202213465.

[424] Chien S, Li S, Shyy YJ. Effects of mechanical forces on signal transduction and gene expression in endothelial cells. Hypertension. 1998;31(1 Pt 2):162–9. 10.1161/01.hyp.31.1.162. 9453297 10.1161/01.hyp.31.1.162

[425] Virani SS, Alonso A, Benjamin EJ, Bittencourt MS, Callaway CW, Carson AP, et al. Heart disease and stroke statistics-2020 update: a report from the American Heart Association. Circulation. 2020;141(9):e139–596. 10.1161/cir.0000000000000757. 31992061 10.1161/CIR.0000000000000757

[426] Kim DH, Lu N, Ma R, Kim YS, Kim RH, Wang S, et al. Epidermal electronics. Science. 2011;333(6044):838–43. 10.1126/science.1206157. 21836009 10.1126/science.1206157

[427] Deng J, Yuk H, Wu J, Varela CE, Chen X, Roche ET, et al. Electrical bioadhesive interface for bioelectronics. Nat Mater. 2021;20(2):229–36. 10.1038/s41563-020-00814-2. 32989277 10.1038/s41563-020-00814-2

[428] Tran PD, Tran TV, Orio M, Torelli S, Truong QD, Nayuki K, et al. Coordination polymer structure and revisited hydrogen evolution catalytic mechanism for amorphous molybdenum sulfide. Nat Mater. 2016;15(6):640–6. 10.1038/nmat4588. 26974410 10.1038/nmat4588 PMC5495159

[429] Ibanez B, James S, Agewall S, Antunes MJ, Bucciarelli-Ducci C, Bueno H, et al. 2017 esc guidelines for the management of acute myocardial infarction in patients presenting with st-segment elevation: The task force for the management of acute myocardial infarction in patients presenting with st-segment elevation of the european society of cardiology (esc). Eur Heart J. 2018;39(2):119–77. 10.1093/eurheartj/ehx393. 28886621 10.1093/eurheartj/ehx393

[430] Guan S, Li J, Zhang K, Li J. Environmentally responsive hydrogels for repair of cardiovascular tissue. Heart Fail Rev. 2021;26(5):1273–85. 10.1007/s10741-020-09934-y. 32076909 10.1007/s10741-020-09934-y

[431] Yu C, Shi M, He S, Yao M, Sun H, Yue Z, et al. Chronological adhesive cardiac patch for synchronous mechanophysiological monitoring and electrocoupling therapy. Nat Commun. 2023;14(1):6226. 10.1038/s41467-023-42008-9. 37803005 10.1038/s41467-023-42008-9 PMC10558550

[432] Han M, Chen L, Aras K, Liang C, Chen X, Zhao H, et al. Catheter-integrated soft multilayer electronic arrays for multiplexed sensing and actuation during cardiac surgery. Nat Biomed Eng. 2020;4(10):997–1009. 10.1038/s41551-020-00604-w. 32895515 10.1038/s41551-020-00604-w PMC8021456

[433] Rogers JA, Huang Y. A curvy, stretchy future for electronics. Proc Natl Acad Sci U S A. 2009;106(27):10875–6. 10.1073/pnas.0905723106. 19567838 10.1073/pnas.0905723106 PMC2708772

[434] Beyaert R, Beaugerie L, Van Assche G, Brochez L, Renauld JC, Viguier M, et al. Cancer risk in immune-mediated inflammatory diseases (imid). Mol Cancer. 2013;12(1):98. 10.1186/1476-4598-12-98. 23987103 10.1186/1476-4598-12-98 PMC3765952

[435] McInnes IB, Gravallese EM. Immune-mediated inflammatory disease therapeutics: Past, present and future. Nat Rev Immunol. 2021;21(10):680–6. 10.1038/s41577-021-00603-1. 34518662 10.1038/s41577-021-00603-1 PMC8436867

[436] Scherlinger M, Richez C, Tsokos GC, Boilard E, Blanco P. The role of platelets in immune-mediated inflammatory diseases. Nat Rev Immunol. 2023;23(8):495–510. 10.1038/s41577-023-00834-4. 36707719 10.1038/s41577-023-00834-4 PMC9882748

[437] Tang X, Wang K, Liu Z, Luo X, Wu M, Ding H, et al. Functional chitosan/hp-β-cd hydrogel for targeted co-delivery of rhubarb-derived nanovesicles and kaempferol for alleviating ulcerative colitis. Carbohydr Polym. 2025;352:123206. 10.1016/j.carbpol.2024.123206. 39843107 10.1016/j.carbpol.2024.123206

[438] Bai Y, Chen T, Zhang Y, Zhao J, Ren C, Zhao Y, et al. Ultrafast in situ formed robust and adhesive photo-crosslinked hydrogel encapsulating curcumin for ulcerative colitis alleviation via regulating inflammation and gut microbiota. Mater Today Bio. 2025;35:102312. 10.1016/j.mtbio.2025.102312. 41080726 10.1016/j.mtbio.2025.102312 PMC12513075

[439] Zhang C, Wang X, Xiao M, Ma J, Qu Y, Zou L, et al. Nano-in-micro alginate/chitosan hydrogel via electrospray technology for orally curcumin delivery to effectively alleviate ulcerative colitis. Mater Design. 2022;221:110894. 10.1016/j.matdes.2022.110894.

[440] Gao M, Yang C, Wu C, Chen Y, Zhuang H, Wang J, et al. Hydrogel-metal-organic-framework hybrids mediated efficient oral delivery of sirna for the treatment of ulcerative colitis. J Nanobiotechnology. 2022;20(1):404. 10.1186/s12951-022-01603-6. 36064365 10.1186/s12951-022-01603-6 PMC9446571

[441] Fang Y, Yu H, Feng X, Xia H, Xu Y, Kankala RK, et al. Microfluidics-enabled polydopamine-coated selenium nanoparticles in hyaluronic hydrogel microspheres for targeted antioxidant and immunomodulatory therapy of ulcerative colitis. Mater Today Bio. 2025;34:102182. 10.1016/j.mtbio.2025.102182. 40838215 10.1016/j.mtbio.2025.102182 PMC12362089

[442] Wang J, Lv X, Li Y, Wu H, Chen M, Yu H, et al. A ros-responsive hydrogel that targets inflamed mucosa to relieve ulcerative colitis by reversing intestinal mucosal barrier loss. J Control Release. 2025;377:606–18. 10.1016/j.jconrel.2024.11.065. 39608456 10.1016/j.jconrel.2024.11.065

[443] Wu Z, Song X, Zhang Y, Lin R. Injectable hydrogel loaded with electrostatic self-assembled structure nanoparticles for the treatment of inflammatory bowel disease. Adv Compos Hybrid Mater. 2025;8(5):367. 10.1007/s42114-025-01423-w.

[444] Zhang S, Kang L, Hu S, Hu J, Fu Y, Hu Y, et al. Carboxymethyl chitosan microspheres loaded hyaluronic acid/gelatin hydrogels for controlled drug delivery and the treatment of inflammatory bowel disease. Int J Biol Macromol. 2021;167:1598–612. 10.1016/j.ijbiomac.2020.11.117. 33220374 10.1016/j.ijbiomac.2020.11.117

[445] Zhang G, Song D, Ma R, Li M, Liu B, He Z, et al. Self-crosslinking hyaluronic acid hydrogel as an enteroprotective agent for the treatment of inflammatory bowel disease. Int J Biol Macromol. 2024;273(Pt 2):132909. 10.1016/j.ijbiomac.2024.132909. 38848832 10.1016/j.ijbiomac.2024.132909

[446] Zhang Y, Liu K, Zheng Y, He J, Huang Y, Gao J, et al. A dual-polysaccharide (carboxylated chitosan-carboxymethyl cellulose sodium) gastrointestinal dynamic response in situ hydrogel for optimized oral targeted inflammatory bowel disease therapy. Carbohydr Polym. 2025;363:123704. 10.1016/j.carbpol.2025.123704. 40441821 10.1016/j.carbpol.2025.123704

[447] Li M, Cui H, Cao Y, Lin Y, Yang Y, Gao M, et al. Deep eutectic solvents-hydrogels for the topical management of rheumatoid arthritis. J Control Release. 2023;354:664–79. 10.1016/j.jconrel.2023.01.050. 36682725 10.1016/j.jconrel.2023.01.050

[448] Wang K, Yin C, Ye X, Chen Q, Wu J, Chen Y, et al. A metabolic driven bio-responsive hydrogel loading psoralen for therapy of rheumatoid arthritis. Small. 2023;19(21):e2207319. 10.1002/smll.202207319. 36869654 10.1002/smll.202207319

[449] Kim T, Suh J, Kim WJ. Polymeric aggregate-embodied hybrid nitric-oxide-scavenging and sequential drug-releasing hydrogel for combinatorial treatment of rheumatoid arthritis. Adv Mater. 2021;33(34):e2008793. 10.1002/adma.202008793. 34235789 10.1002/adma.202008793

[450] Geng W, Zhao J, Tao B, Yang Y, Duan Q, Gao P, et al. Regulation of rheumatoid arthritis microenvironment via a self-healing injectable hydrogel for improved inflammation elimination and bone repair. Bioact Mater. 2024;36:287–300. 10.1016/j.bioactmat.2024.03.002. 38496033 10.1016/j.bioactmat.2024.03.002 PMC10940865

[451] Kumar Podder A, Mohamed M A, Seidman R A, Tseropoulos G, Polanco J J, Lei P, et al. Injectable shear-thinning hydrogels promote oligodendrocyte progenitor cell survival and remyelination in the central nervous system. Sci Adv. 2024;10(28):eadk9918. 10.1126/sciadv.adk9918. 10.1126/sciadv.adk9918 PMC11244542 38996029

[452] Hadidi N, Pazuki G. Preparation, characterization and in-vivo efficacy study of glatiramer acetate (ga)-hydrogel-microparticles as novel drug delivery system for ga in rrms. Sci Rep. 2022;12(1):22042. 10.1038/s41598-022-26640-x. 36543898 10.1038/s41598-022-26640-x PMC9772231

[453] Shobeirean A, Attar H, Varshochian R, Rezvanfar MA. Glatiramer acetate in situ forming gel, a new approach for multiple sclerosis treatment. Daru. 2024;32(2):649–64. 10.1007/s40199-024-00532-z. 39225953 10.1007/s40199-024-00532-z PMC11554603

[454] Matías-Guiu J, Matías-Guiu JA, Montero-Escribano P, Barcia JA, Canales-Aguirre AA, Mateos-Diaz JC, et al. Particles containing cells as a strategy to promote remyelination in patients with multiple sclerosis. Front Neurol. 2020;11:638. 10.3389/fneur.2020.00638. 32733364 10.3389/fneur.2020.00638 PMC7358567

[455] Martin-Saldaña S, Al-Waeel M, Bagnoli E, Chevalier MT, Bu Y, Lally C, et al. Hyaluronic acid-based hydrogels modulate neuroinflammation and extracellular matrix remodelling in multiple sclerosis: Insights from a primary cortical cell model. Mater Horiz. 2025;12(11):3841–54. 10.1039/d4mh01598c. 40067190 10.1039/d4mh01598c

[456] Zhu S, Huang D, Luan Q, Li Y, Gan J, Zhao Y, et al. Mcp-1 and il-4 encapsulated hydrogel particles with macrophages enrichment and polarization capabilities for systemic lupus erythematosus treatment. Nano Res. 2024;17(9):8316–24. 10.1007/s12274-024-6794-z.

[457] Zhu H, Huang D, Nie M, Zhao Y, Sun L. Dexamethasone loaded DNA scavenger nanogel for systemic lupus erythematosus treatment. Bioact Mater. 2025;43:330–9. 10.1016/j.bioactmat.2024.08.030. 40115883 10.1016/j.bioactmat.2024.08.030 PMC11923376

[458] Zhu H, Kong B, Nie M, Zhao C, Liu R, Xie Y, et al. Ecm-inspired peptide dendrimer microgels with human mscs encapsulation for systemic lupus erythematosus treatment. Nano Today. 2022;43:101454. 10.1016/j.nantod.2022.101454.

[459] Nie M, Chen G, Zhao C, Gan J, Alip M, Zhao Y, et al. Bio-inspired adhesive porous particles with human mscs encapsulation for systemic lupus erythematosus treatment. Bioact Mater. 2021;6(1):84–90. 10.1016/j.bioactmat.2020.07.018. 32817916 10.1016/j.bioactmat.2020.07.018 PMC7419256

[460] Finckh A, Gilbert B, Hodkinson B, Bae SC, Thomas R, Deane KD, et al. Global epidemiology of rheumatoid arthritis. Nat Rev Rheumatol. 2022;18(10):591–602. 10.1038/s41584-022-00827-y. 36068354 10.1038/s41584-022-00827-y

[461] Weyand CM, Goronzy JJ. The immunology of rheumatoid arthritis. Nat Immunol. 2021;22(1):10–8. 10.1038/s41590-020-00816-x. 33257900 10.1038/s41590-020-00816-x PMC8557973

[462] Siddiqui B, Ur Rehman A, Gul R, Chaudhery I, Shah KU, Ahmed N. Folate decorated chitosan-chondroitin sulfate nanoparticles loaded hydrogel for targeting macrophages against rheumatoid arthritis. Carbohydr Polym. 2024;327:121683. 10.1016/j.carbpol.2023.121683. 38171692 10.1016/j.carbpol.2023.121683

[463] Li C, Du Y, Lv H, Zhang J, Zhuang P, Yang W, et al. Injectable amphipathic artesunate prodrug-hydrogel microsphere as gene/drug nano-microplex for rheumatoid arthritis therapy. Adv Funct Mater. 2022;32(44):2206261. 10.1002/adfm.202206261.

[464] Wu Y, Ge Y, Wang Z, Zhu Y, Tian T, Wei J, et al. Synovium microenvironment-responsive injectable hydrogel inducing modulation of macrophages and elimination of synovial fibroblasts for enhanced treatment of rheumatoid arthritis. J Nanobiotechnology. 2024;22(1):188. 10.1186/s12951-024-02465-w. 38632657 10.1186/s12951-024-02465-w PMC11025172

[465] Wu Y, Wang Z, Ge Y, Zhu Y, Tian T, Wei J, et al. Microenvironment responsive hydrogel exerting inhibition of cascade immune activation and elimination of synovial fibroblasts for rheumatoid arthritis therapy. J Control Release. 2024;370:747–62. 10.1016/j.jconrel.2024.05.021. 38740094 10.1016/j.jconrel.2024.05.021

[466] Song Y, Milichko VA, Ding Z, Li W, Kang B, Dou Y, et al. Double cross-linked hydrogel for intra-articular injection as modality for macrophages metabolic reprogramming and therapy of rheumatoid arthritis. Adv Funct Mater. 2025;35(32):2502880. 10.1002/adfm.202502880.

[467] Yuan H, Cui S, Yang L, Cui J, Wang X, Ding M, et al. Efficacy of non-conventional synthetic dmards for patients with rheumatoid arthritis-associated interstitial lung disease: a systematic review and meta-analysis. RMD Open. 2023. 10.1136/rmdopen-2023-003487. 37899093 10.1136/rmdopen-2023-003487 PMC10619071

[468] Lee YM, Lee S, Kim WJ. Nitric oxide scavengers based on o-phenylenediamine for the treatment of rheumatoid arthritis. Biomater Sci. 2023;11(7):2395–404. 10.1039/d2bm01994a. 36786425 10.1039/d2bm01994a

[469] Arnaud L, Chasset F, Martin T. Immunopathogenesis of systemic lupus erythematosus: An update. Autoimmun Rev. 2024;23(10):103648. 10.1016/j.autrev.2024.103648. 39343084 10.1016/j.autrev.2024.103648

[470] Patiño-Martinez E, Kaplan MJ. Immunometabolism in systemic lupus erythematosus. Nat Rev Rheumatol. 2025;21(7):377–95. 10.1038/s41584-025-01267-0. 40524030 10.1038/s41584-025-01267-0

[471] Wang PF, Jiang F, Zeng QM, Yin WF, Hu YZ, Li Q, et al. Mitochondrial and metabolic dysfunction of peripheral immune cells in multiple sclerosis. J Neuroinflammation. 2024;21(1):28. 10.1186/s12974-024-03016-8. 38243312 10.1186/s12974-024-03016-8 PMC10799425

[472] Woo MS, Engler JB, Friese MA. The neuropathobiology of multiple sclerosis. Nat Rev Neurosci. 2024;25(7):493–513. 10.1038/s41583-024-00823-z. 38789516 10.1038/s41583-024-00823-z

[473] Giannopapas V, Smyrni V, Kitsos DK, Stefanou MI, Theodorou A, Tzartos JS, et al. Cancer in multiple sclerosis patients following prolonged exposure to disease-modifying therapies (dmts): A systematic review and meta-analysis. J Neurol. 2025;272(2):162. 10.1007/s00415-024-12882-4. 39849174 10.1007/s00415-024-12882-4 PMC11757934

[474] Androdias G, Lünemann JD, Maillart E, Amato MP, Audoin B, Bruijstens AL, et al. De-escalating and discontinuing disease-modifying therapies in multiple sclerosis. Brain. 2025;148(5):1459–78. 10.1093/brain/awae409. 39707906 10.1093/brain/awae409 PMC12073975

[475] Eigel D, Zoupi L, Sekizar S, Welzel PB, Werner C, Williams A, et al. Cryogel scaffolds for regionally constrained delivery of lysophosphatidylcholine to central nervous system slice cultures: A model of focal demyelination for multiple sclerosis research. Acta Biomater. 2019;97:216–29. 10.1016/j.actbio.2019.08.030. 31425890 10.1016/j.actbio.2019.08.030

[476] Napolitano F, Giudice V, D’Esposito V, Prevete N, Scala P, de Paulis A, et al. Cell-free regenerative medicine: Identifying the best source of mesenchymal stem cells for skin therapy in systemic sclerosis. Front Cell Dev Biol. 2025;13:1518412. 10.3389/fcell.2025.1518412. 40046231 10.3389/fcell.2025.1518412 PMC11880212

[477] Stober CB, Ellis L, Goodall JC, Veldhoen M, Gaston JSH. Metabolic stress expands polyfunctional, proinflammatory th(17) cells in patients with psoriatic arthritis for whom there is interleukin-23-independent interleukin-17 production. Arthritis Rheumatol. 2025;77(7):842–53. 10.1002/art.43095. 39711043 10.1002/art.43095 PMC12209755

[478] Nie M, Kong B, Chen G, Xie Y, Zhao Y, Sun L. Mscs-laden injectable self-healing hydrogel for systemic sclerosis treatment. Bioact Mater. 2022;17:369–78. 10.1016/j.bioactmat.2022.01.006. 35386467 10.1016/j.bioactmat.2022.01.006 PMC8964965

[479] Kalarikkal SP, Kumar MN, Rajendran S, Bethi CMS, Ravilla J, Narayanan J, et al. Natural plant-derived nanovesicles for effective psoriasis therapy via dual modulation of il-17 and nrf2 pathway. iScience. 2025;28(6):112556. 10.1016/j.isci.2025.112556. 40487455 10.1016/j.isci.2025.112556 PMC12144462

[480] Su D, Li M, Xie Y, Xu Z, Lv G, Jiu Y, et al. Gut commensal bacteria parabacteroides goldsteinii-derived outer membrane vesicles suppress skin inflammation in psoriasis. J Control Release. 2025;377:127–45. 10.1016/j.jconrel.2024.11.014. 39532207 10.1016/j.jconrel.2024.11.014

[481] Tang H, Yang J, Wu Z, Jiang B, Sun W, Zhang C, et al. Trends in global and national infertility and factors associated with primary infertile couples in recent middle-aged chinese. PLoS ONE. 2025;20(11):e0335926. 10.1371/journal.pone.0335926. 41218083 10.1371/journal.pone.0335926 PMC12604783

[482] Zeng G, Liu L, Wang Y, Yu J, Wang H, Li F. Global, regional, and national burden and trends of reproductive-aged male and female infertility from 1990–2021. Front Endocrinol (Lausanne). 2025;16:1506229. 10.3389/fendo.2025.1506229. 40979725 10.3389/fendo.2025.1506229 PMC12443578

[483] Zhang J, Shi C, Sun J, Niu J. Analysis of factors affecting the prognosis of patients with intrauterine adhesions after transcervical resection of adhesions. Fertil Steril. 2024;122(2):365–72. 10.1016/j.fertnstert.2024.03.016. 38518992 10.1016/j.fertnstert.2024.03.016

[484] Magnus MC, Skåra KH, Carlsen E, Gjerdevik M, Ramlau-Hansen CH, Myrskylä M, et al. Use of assisted reproductive technologies for male and female infertility and perinatal outcomes. Fertil Steril. 2025;124(2):270–80. 10.1016/j.fertnstert.2025.02.013. 39954979 10.1016/j.fertnstert.2025.02.013

[485] Shi H, Li QY, Li H, Wang HY, Fan CX, Dong QY, et al. Ros-induced oxidative stress is a major contributor to sperm cryoinjury. Hum Reprod. 2024;39(2):310–25. 10.1093/humrep/dead250. 38011909 10.1093/humrep/dead250

[486] Wang L, Cheng LC, Chen Y, Zhai H, Chen Z, Ren T, et al. Dual-crosslinked bioactive hydrogel scaffold for accelerated repair of genital tract defect. Adv Funct Mater. 2024;34(41):2405966. 10.1002/adfm.202405966.

[487] Zhang T, Li D, Wang Y, Zhang C, Yang W, Gao G. Delivering umbilical cord mesenchymal stem cell exosomes through hydrogel ameliorates vaginal atrophy in ovariectomized rats. Aging (Albany N Y). 2023;15(23):14292–305. 10.18632/aging.205302. 10.18632/aging.205302 PMC10756086 38059876

[488] Qi J, Li X, Cao Y, Long Y, Lai J, Yao Y, et al. Locationally activated prp via an injectable dual-network hydrogel for endometrial regeneration. Biomaterials. 2024;309:122615. 10.1016/j.biomaterials.2024.122615. 38759486 10.1016/j.biomaterials.2024.122615

[489] Fan G, Lu Y, Li Y, Zhang J, Wang Y, Lee P, et al. Lactobacillus-loaded easily injectable hydrogel promotes endometrial repair via long-term retention and microenvironment modulation. ACS Nano. 2025;19(4):4440–51. 10.1021/acsnano.4c13593. 39823410 10.1021/acsnano.4c13593

[490] Yang C, Chung N, Song C, Youm HW, Lee K, Lee JR. Promotion of angiogenesis toward transplanted ovaries using nitric oxide releasing nanoparticles in fibrin hydrogel. Biofabrication. 2021. 10.1088/1758-5090/ac3f28. 34852328 10.1088/1758-5090/ac3f28

[491] Nason-Tomaszewski CE, Thomas EE, Matera DL, Baker BM, Shikanov A. Extracellular matrix-templating fibrous hydrogels promote ovarian tissue remodeling and oocyte growth. Bioact Mater. 2024;32:292–303. 10.1016/j.bioactmat.2023.10.001. 37876554 10.1016/j.bioactmat.2023.10.001 PMC10590725

[492] Wang Z, Liu X, Ye T, Zhai Z, Wu K, Kuang Y, et al. 3d-printed perfused models of the penis for the study of penile physiology and for restoring erectile function in rabbits and pigs. Nat Biomed Eng. 2025;9(8):1276–89. 10.1038/s41551-025-01367-y. 40038440 10.1038/s41551-025-01367-y

[493] Ji M, Chen D, Shu Y, Dong S, Zhang Z, Zheng H, et al. The role of mechano-regulated yap/taz in erectile dysfunction. Nat Commun. 2023;14(1):3758. 10.1038/s41467-023-39009-z. 37353497 10.1038/s41467-023-39009-z PMC10290143

[494] Ru F, Velmurugan R, Li C, Mu Y, Tian H, Zhou L, et al. Bioengineered injectable HAMA/GELMA hydrogel encapsulating exosomes loaded lycopene mitigates deoxynivalenol-induced testicular injury via apoptotic pathway modulation. J Biol Eng. 2025;19(1):86. 10.1186/s13036-025-00555-3. 41035067 10.1186/s13036-025-00555-3 PMC12487079

[495] Ye Y, Zhong W, Luo R, Wen H, Ma Z, Qi S, et al. Thermosensitive hydrogel with emodin-loaded triple-targeted nanoparticles for a rectal drug delivery system in the treatment of chronic non-bacterial prostatitis. J Nanobiotechnology. 2024;22(1):33. 10.1186/s12951-023-02282-7. 38238760 10.1186/s12951-023-02282-7 PMC10795337

[496] Kim J, Shim JS, Han BH, Kim HJ, Park J, Cho IJ, et al. Hydrogel-based hybridization chain reaction (hcr) for detection of urinary exosomal mirnas as a diagnostic tool of prostate cancer. Biosens Bioelectron. 2021;192:113504. 10.1016/j.bios.2021.113504. 34298498 10.1016/j.bios.2021.113504

[497] Capogrosso P, Albersen M, Burnett AL, Cakir OO, Dehó F, Morgado LA, et al. Erectile dysfunction: Update on clinical management. Eur Urol. 2025;88(4):388–99. 10.1016/j.eururo.2025.05.004. 40393866 10.1016/j.eururo.2025.05.004

[498] Zhu D, Pham QM, Wang C, Colonnello E, Yannas D, Nguyen BH, et al. Erectile dysfunction and oxidative stress: a narrative review. Int J Mol Sci. 2025. 10.3390/ijms26073073. 40243750 10.3390/ijms26073073 PMC11988752

[499] Ismail EA, El-Sakka AI. An overview of conventional and investigational phosphodiesterase 5 inhibitors for treating erectile dysfunction and other conditions. Expert Opin Investig Drugs. 2024;33(9):925–38. 10.1080/13543784.2024.2388569. 39096237 10.1080/13543784.2024.2388569

[500] Porst H, Lewis R, Virag R, Goldstein I. A comprehensive history of injection therapy for erectile dysfunction, 1982-2023. Sex Med Rev. 2024;12(3):419–33. 10.1093/sxmrev/qeae020. 38644056 10.1093/sxmrev/qeae020

[501] Köhler TS, Kloner RA, Rosen RC, Burnett AL, Blaha MJ, Ganz P, et al. The princeton iv consensus recommendations for the management of erectile dysfunction and cardiovascular disease. Mayo Clin Proc. 2024;99(9):1500–17. 10.1016/j.mayocp.2024.06.002. 39115509 10.1016/j.mayocp.2024.06.002

[502] Jia M, Wang J, Lin C, Zhang Q, Xue Y, Huang X, et al. Hydrogel strategies for female reproduction dysfunction. ACS Nano. 2024;18(44):30132–52. 10.1021/acsnano.4c05634. 39437800 10.1021/acsnano.4c05634

[503] Luo X, Jia K, Xing J, Yi J. The utilization of nanotechnology in the female reproductive system and related disorders. Heliyon. 2024;10(3):e25477. 10.1016/j.heliyon.2024.e25477. 38333849 10.1016/j.heliyon.2024.e25477 PMC10850912

[504] Zhang D, Li H. Epidemiology, etiology and treatment of female vaginal injury. Reprod Health. 2025;22(1):65. 10.1186/s12978-025-02017-x. 40329296 10.1186/s12978-025-02017-x PMC12057040

[505] Hu X, Wu H, Yong X, Wang Y, Yang S, Fan D, et al. Cyclical endometrial repair and regeneration: Molecular mechanisms, diseases, and therapeutic interventions. MedComm (2020). 2023;4(6):e425. 10.1002/mco2.425. 10.1002/mco2.425 PMC10691302 38045828

[506] Yu H, Yu X, Huang Y, Yu T, Lan H, Zhang Q, et al. Engineering biocompatible carbon dots nano-enzymes hydrogel for efficient antioxidative and anti-inflammatory treatment of dry eye disease. J Control Release. 2025;381:113490. 10.1016/j.jconrel.2025.01.081. 39884436 10.1016/j.jconrel.2025.01.081

[507] Li J, Zhang X, Zheng Q, Zhu Y, Wang H, Ma H, et al. Comparative evaluation of silicone hydrogel contact lenses and autologous serum for management of Sjögren syndrome-associated dry eye. Cornea. 2015;34(9):1072–8. 10.1097/ico.0000000000000515. 26147835 10.1097/ICO.0000000000000515

[508] Hao T, Tang L, Xu Q, Wang W, Li Z, Shen Y, et al. Silk fibroin formed bioadhesive ophthalmic gel for dry eye syndrome treatment. AAPS PharmSciTech. 2024;25(5):92. 10.1208/s12249-024-02792-z. 38684590 10.1208/s12249-024-02792-z

[509] Gong B, Liu Y, Li H, Ju X, Li D, Zou Y, et al. A silk fibroin nanoparticle hydrogel loaded with NK1R antagonist has synergistic anti-inflammatory and reparative effects on dry eye disease. Adv Sci. 2025;12(15):e2404835. 10.1002/advs.202404835. 10.1002/advs.202404835 PMC12005769 39985258

[510] Yu Y, Chow DWY, Lau CML, Zhou G, Back W, Xu J, et al. A bioinspired synthetic soft hydrogel for the treatment of dry eye. Bioeng Transl Med. 2021;6(3):e10227. 10.1002/btm2.10227. 34589602 10.1002/btm2.10227 PMC8459603

[511] Zhao Y, Ding Q, He Q, Zu T, Rong Z, Wu Y, et al. Reno protective potential of taxifolin liposomes modified by chitosan in diabetic mice. Int J Biol Macromol. 2025;306(Pt 2):141464. 10.1016/j.ijbiomac.2025.141464. 40015419 10.1016/j.ijbiomac.2025.141464

[512] Yu M, Wang D, Zhong D, Xie W, Luo J. Adropin carried by reactive oxygen species-responsive nanocapsules ameliorates renal lipid toxicity in diabetic mice. ACS Appl Mater Interfaces. 2022;14(33):37330–44. 10.1021/acsami.2c06957. 35951354 10.1021/acsami.2c06957

[513] Jumelle C, Yung A, Sani ES, Taketani Y, Gantin F, Bourel L, et al. Development and characterization of a hydrogel-based adhesive patch for sealing open-globe injuries. Acta Biomater. 2022;137:53–63. 10.1016/j.actbio.2021.10.021. 34673229 10.1016/j.actbio.2021.10.021 PMC8678346

[514] Zhao L, Shi Z, Sun X, Yu Y, Wang X, Wang H, et al. Natural dual-crosslinking bioadhesive hydrogel for corneal regeneration in large-size defects. Adv Healthc Mater. 2022;11(21):e2201576. 10.1002/adhm.202201576. 36040708 10.1002/adhm.202201576

[515] Zhang S, Zhou H, Huang C, Sun J, Qu X, Lu Y. A novel corneal adhesive based on functionally coupled PEG-lysozyme hydrogel for wound closure after surgical eye surgery. Chin Chem Lett. 2022;33(9):4321–5. 10.1016/j.cclet.2022.01.024.

[516] Shen X, Li S, Zhao X, Han J, Chen J, Rao Z, et al. Dual-crosslinked regenerative hydrogel for sutureless long-term repair of corneal defect. Bioact Mater. 2023;20:434–48. 10.1016/j.bioactmat.2022.06.006. 35800407 10.1016/j.bioactmat.2022.06.006 PMC9234351

[517] Wang Y, Liu H, Yang X, Shi Z, Li J, Xue L, et al. A responsive hydrogel-based microneedle system for minimally invasive glucose monitoring. Smart Mater Med. 2023;4:69–77. 10.1016/j.smaim.2022.07.006.

[518] Darmau B, Sacchi M, Texier I, Gross AJ. Self-extracting dextran-based hydrogel microneedle arrays with an interpenetrating bioelectroenzymatic sensor for transdermal monitoring with matrix protection. Adv Healthc Mater. 2025;14(2):e2403209. 10.1002/adhm.202403209. 39580665 10.1002/adhm.202403209 PMC11729986

[519] GhavamiNejad P, GhavamiNejad A, Zheng H, Dhingra K, Samarikhalaj M, Poudineh M. A conductive hydrogel microneedle-based assay integrating pedot:Pss and ag-pt nanoparticles for real-time, enzyme-less, and electrochemical sensing of glucose. Adv Healthc Mater. 2023;12(1):e2202362. 10.1002/adhm.202202362. 36183355 10.1002/adhm.202202362

[520] Jiang L, Yao L, Xie H, Liu C, Jin W. An aggregation interpenetrating induced poly(dopamine) conductive hydrogel grid for efficient glucose detection. Biosens Bioelectron. 2025;290:117951. 10.1016/j.bios.2025.117951. 40914020 10.1016/j.bios.2025.117951

[521] Caffin F, Boccara D, Piérard C. The use of hydrogel dressings in sulfur mustard-induced skin and ocular wound management. Biomedicines. 2023. 10.3390/biomedicines11061626. 37371720 10.3390/biomedicines11061626 PMC10295658

[522] Thacker M, Singh V, Basu S, Singh S. Biomaterials for dry eye disease treatment: Current overview and future perspectives. Exp Eye Res. 2023;226:109339. 10.1016/j.exer.2022.109339. 36470431 10.1016/j.exer.2022.109339

[523] Lin D, Lei L, Shi S, Li X. Stimulus-responsive hydrogel for ophthalmic drug delivery. Macromol Biosci. 2019;19(6):e1900001. 10.1002/mabi.201900001. 31026123 10.1002/mabi.201900001

[524] Kong GY, Henderson RH, Sandhu SS, Essex RW, Allen PJ, Campbell WG. Wound-related complications and clinical outcomes following open globe injury repair. Clin Exp Ophthalmol. 2015;43(6):508–13. 10.1111/ceo.12511. 25688653 10.1111/ceo.12511

[525] Bourne RRA, Flaxman SR, Braithwaite T, Cicinelli MV, Das A, Jonas JB, et al. Magnitude, temporal trends, and projections of the global prevalence of blindness and distance and near vision impairment: A systematic review and meta-analysis. Lancet Glob Health. 2017;5(9):e888–97. 10.1016/S2214-109X(17)30293-0. 28779882 10.1016/S2214-109X(17)30293-0

[526] Baudouin C, Aragona P, Messmer E M, Tomlinson A, Calonge M, Boboridis K G, et al. Role of hyperosmolarity in the pathogenesis and management of dry eye disease: Proceedings of the ocean group meeting. Ocul Surf. 2013;11(4):246–58. 10.1016/j.jtos.2013.07.003. 10.1016/j.jtos.2013.07.003 24112228

[527] Choi SW, Cha BG, Kim J. Therapeutic contact lens for scavenging excessive reactive oxygen species on the ocular surface. ACS Nano. 2020;14(2):2483–96. 10.1021/acsnano.9b10145. 31935066 10.1021/acsnano.9b10145

[528] Ou L, Wu Z, Hu X, Huang J, Yi Z, Gong Z, et al. A tissue-adhesive f127 hydrogel delivers antioxidative copper-selenide nanoparticles for the treatment of dry eye disease. Acta Biomater. 2024;175:353–68. 10.1016/j.actbio.2023.12.021. 38110136 10.1016/j.actbio.2023.12.021

[529] Yang M, Chen X, Chen Z, Zhao N, Zeng Z, Huang X, et al. Thermoresponsive antioxidant metal-free carbon nanodot hydrogel: An effective therapeutic approach for ocular surface disease. Sci Adv. 2025;11(30):eadt8775. 10.1126/sciadv.adt8775. 10.1126/sciadv.adt8775 PMC12292845 40712015

[530] Zhang Y, Lin T, Jiang A, Zhao N, Gong L. Vision-related quality of life and psychological status in chinese women with sjogren’s syndrome dry eye: A case-control study. BMC Womens Health. 2016;16(1):75. 10.1186/s12905-016-0353-z. 27955668 10.1186/s12905-016-0353-z PMC5154065

[531] Chen T, Liu R, Chen Q, Feng X, Kong B, Zhao Y. Msc-encapsulated porous microparticle eye drops for autoimmune dry eye disease treatment in nod mice. Sci Adv. 2025;11(39):eadu9772. 10.1126/sciadv.adu9772. 10.1126/sciadv.adu9772 PMC12459467 40991713

[532] Olansky L, Kennedy L. Finger-stick glucose monitoring: Issues of accuracy and specificity. Diabetes Care. 2010;33(4):948–9. 10.2337/dc10-0077. 20351231 10.2337/dc10-0077 PMC2845057

[533] Jiang L, Ao Q, Tong X, Lv X, Song Y, Tang J. A biocatalytic cascade in enzyme/metal continuous-microflow microgel with stable intermediate channel for point-of-care biosensing. Biosens Bioelectron. 2024;248:115965. 10.1016/j.bios.2023.115965. 38176253 10.1016/j.bios.2023.115965

[534] Kim SK, Lee GH, Jeon C, Han HH, Kim SJ, Mok JW, et al. Bimetallic nanocatalysts immobilized in nanoporous hydrogels for long-term robust continuous glucose monitoring of smart contact lens. Adv Mater. 2022;34(18):e2110536. 10.1002/adma.202110536. 35194844 10.1002/adma.202110536 PMC10782562

[535] Kanokpaka P, Chang Y-H, Chang C-C, Rinawati M, Wang P-C, Chang L-Y, et al. Enabling glucose adaptive self-healing hydrogel based triboelectric biosensor for tracking a human perspiration. Nano Energy. 2023;112:108513. 10.1016/j.nanoen.2023.108513.

[536] Mandal SS, Choudhury AM, Gupta A, Maiti P. An injectable cyclodextrin extended polyurethane/carboxymethyl cellulose hydrogel for controlled release of insulin: In-vitro and in-vivo diabetic animal model study. Carbohydr Polym. 2025;356:123396. 10.1016/j.carbpol.2025.123396. 40049968 10.1016/j.carbpol.2025.123396

[537] Kirac CO, Ipekci SH, Kebapcilar L. A new experience for incorrect insulin administration. Acta Diabetol. 2018;55(9):985–6. 10.1007/s00592-018-1184-1. 29934655 10.1007/s00592-018-1184-1

[538] GhavamiNejad A, Flynn CD, Geraili A, Mirzaie S, Esmaeili F, Zargartalebi H, et al. Continuous insulin monitoring using an antibody-protecting zwitterionic microneedle patch. Nat Biomed Eng. 2026;10(3):445–57. 10.1038/s41551-025-01477-7. 40775522 10.1038/s41551-025-01477-7 PMC13071906

[539] Maity B, Moorthy H, Govindaraju T. Glucose-responsive self-regulated injectable silk fibroin hydrogel for controlled insulin delivery. ACS Appl Mater Interfaces. 2023;15(43):49953–63. 10.1021/acsami.3c07060. 37847862 10.1021/acsami.3c07060

[540] Zhou Y, Zhao C, Shi Z, Heger Z, Jing H, Shi Z, et al. A glucose-responsive hydrogel inhibits primary and secondary brb injury for retinal microenvironment remodeling in diabetic retinopathy. Adv Sci (Weinh). 2024;11(32):e2402368. 10.1002/advs.202402368. 39031576 10.1002/advs.202402368 PMC11348052

[541] Ding Q, Liu W, Liu X, Ding C, Zhao Y, Dong L, et al. Polyvinylpyrrolidone-modified taxifolin liposomes promote liver repair by modulating autophagy to inhibit activation of the tlr4/nf-κb signaling pathway. Front Bioeng Biotechnol. 2022;10:860515. 10.3389/fbioe.2022.860515. 35721857 10.3389/fbioe.2022.860515 PMC9199375

[542] Kumar KG, Trevaskis JL, Lam DD, Sutton GM, Koza RA, Chouljenko VN, et al. Identification of adropin as a secreted factor linking dietary macronutrient intake with energy homeostasis and lipid metabolism. Cell Metab. 2008;8(6):468–81. 10.1016/j.cmet.2008.10.011. 19041763 10.1016/j.cmet.2008.10.011 PMC2746325

[543] Zang H, Jiang F, Cheng X, Xu H, Hu X. Serum adropin levels are decreased in Chinese type 2 diabetic patients and negatively correlated with body mass index. Endocr J. 2018;65(7):685–91. 10.1507/endocrj.EJ18-0060. 29669965 10.1507/endocrj.EJ18-0060

